# Graphene oxide-catalyzed trifluoromethylation of alkynes with quinoxalinones and Langlois' reagent†

**DOI:** 10.1039/d1ra07014b

**Published:** 2021-12-01

**Authors:** Hong Li, Xiangjun Peng, Liang Nie, Lin Zhou, Ming Yang, Fan Li, Jian Hu, Zhiyang Yao, Liangxian Liu

**Affiliations:** Key Laboratory of Organo-Pharmaceutical Chemistry of Jiangxi Province, Gannan Normal University Ganzhou Jiangxi 341000 P. R. China; Key Laboratory of Prevention and Treatment of Cardiovascular and Cerebrovascular Diseases of Ministry of Education, Gannan Medical University Ganzhou 341000 P. R. China liuliangxian1962@163.com

## Abstract

The direct C–H trifluoromethylation of alkynes and quinoxalinones has been achieved using a graphene oxide/Langlois' reagent system. This multi-component tandem reaction using graphene oxide as the catalyst and Langlois' reagent as the robust CF_3_ radical source results in the formation of olefinic C–CF_3_ to access a series of 3-trifluoroalkylated quinoxalin-2(1*H*)-ones.

## Abstract 2

The direct C–H trifluoromethylation of alkynes and quinoxalinones using a graphene oxide/Langlois' reagent system.

## Graphical abstract

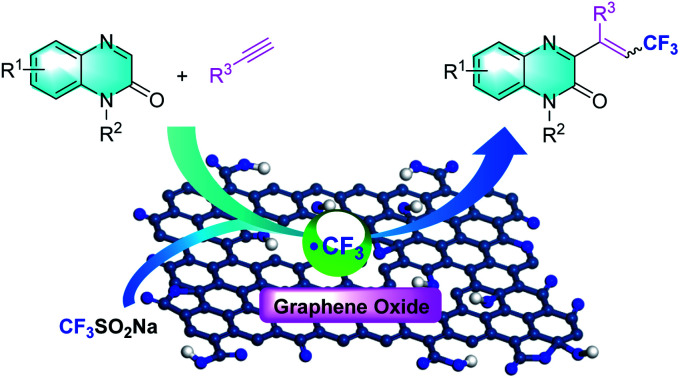

## Introduction

Graphene oxide (GO), exfoliated from oxidized graphite, has a large π-network and specific surface area, abundant defects and a wide range of oxygen functional groups involving alcohols, epoxides, carboxylic acids and bridging 1,3-ethers.^1^ This ultrathin 2D carbocatalyst, using its inherent reactivity of oxidation, acidity and catalytic activity, has been successfully applied to oxidation, acid- or base-catalyzed transformations, oxidative coupling of amines, hydration of alkynes, aza-Michael addition reactions of amines and α,β-unsaturated compounds, ring opening of epoxy compounds, and dehydrogenation–oxidation of nitrogen heterocycles.^2^ However, GO as a catalyst in organic transformation still faces serious challenges, compared with its applications in electronics, optical materials, solar cells and biosensors.^3^

**Fig. 1:**
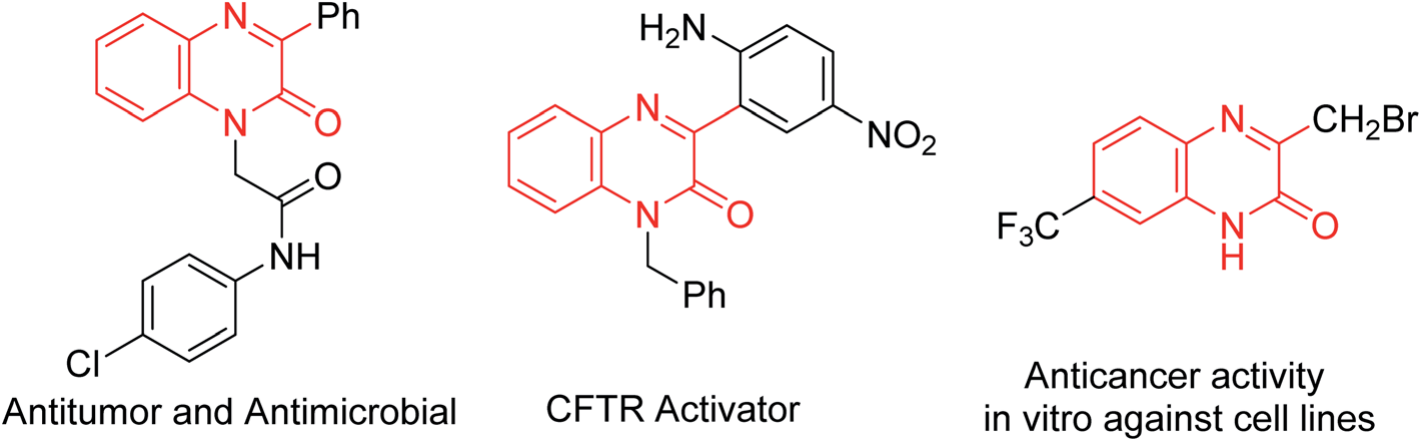
Representative biologically active quinoxalin-2(1*H*)-ones.

Quinoxalin-2(1*H*)-ones are important compounds that exist in various bioactive natural products and pharmaceutical compounds, especially CF_3_-containing quinoxalin-2(1*H*)-ones showing potent anticancer activities (Fig. 1).^4^

**Scheme 1:**
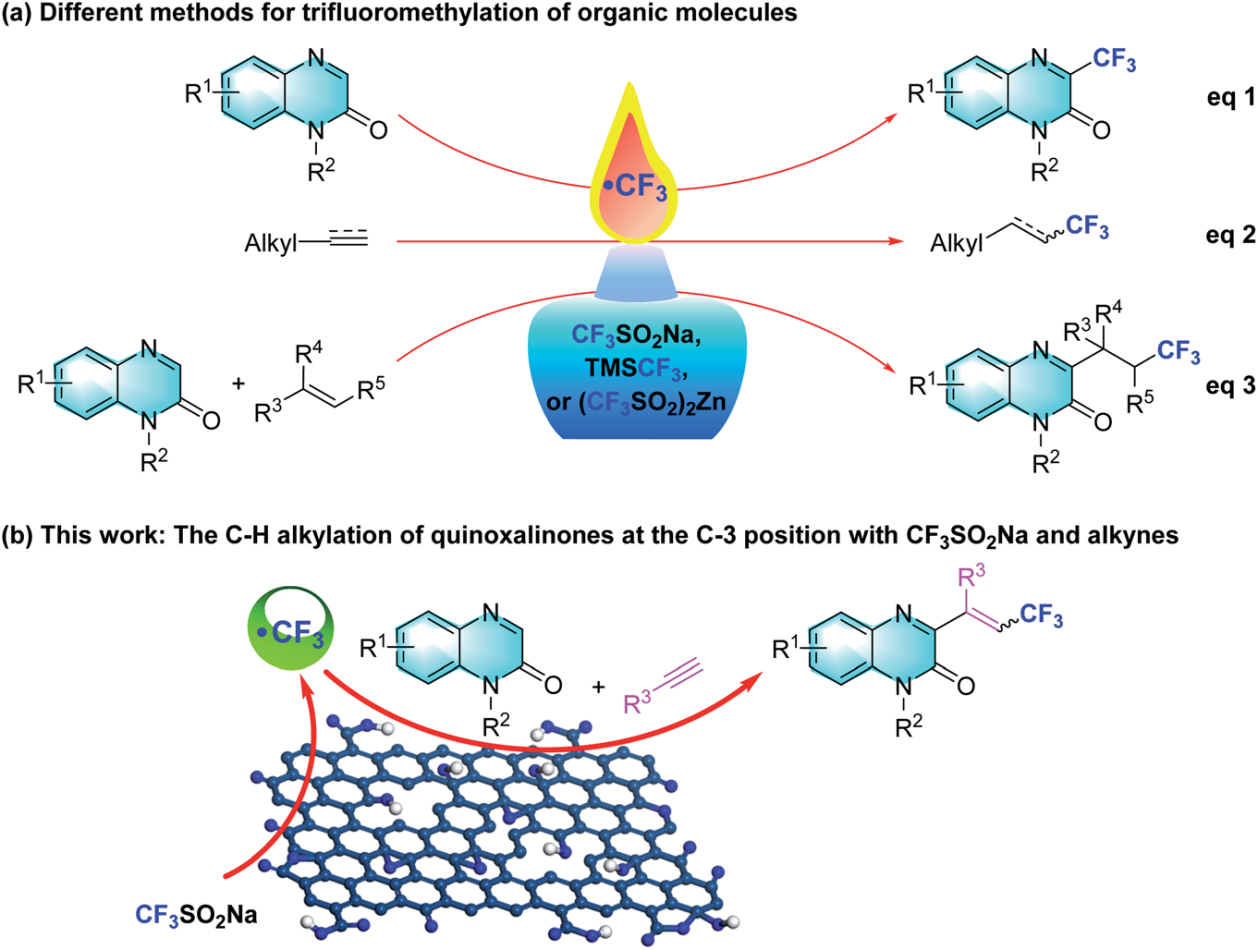
Various trifluoromethylation strategies of organic molecules.

A tremendous amount of trifluoromethylation strategies have been developed and are widespread in the pharmaceutical, agrochemical and material science fields ever since the incorporation of the CF_3_ group was introduced into different organic molecules.^5^ Various trifluoroalkylated regents, such as Langlois (sodium trifluoromethanesulfinate, NaSO_2_CF_3_),^6^ Togni,^7^ Umemoto,^8^ and Ruppert-Prakash,^9^ have been developed and successfully employed in trifluoromethylation reactions. In this regard, many research groups have explored diverse synthetic approaches involving inorganic/organic oxidants, photocatalysis, or electrocatalysis, which synthesize 3-trifluoromethylquinoxalin-2(1*H*)-ones using quinoxalin-2(1*H*)-ones and CF_3_SO_2_Na, TMSCF_3_, or Zn(SO_2_CF_3_)_2_ as the trifluoromethylated agent (Scheme 1, eqn (1)).^10^ Within this arena, Langlois' reagent, as an electrophilic trifluoromethyl radical, has been frequently used in the addition of electron-rich olefins and alkynes (Scheme 1, eqn (2)).^11^ In particular, a few three-component protocols of “CF_3_”-radical addition to terminal or unactivated alkenes with Langlois' reagent have delivered various 3-trifluoroalkylated quinoxalin-2(1*H*)-ones under metal-free conditions (Scheme 1, eqn (3)).^12^ These trifluoroalkylated reactions exhibit mild reaction conditions, low catalyst loading, ubiquity and versatile functionality of directing C–CF_3_ activation. Inspired by these successful discoveries, we turned our attention to the multi-component trifluoromethylation of alkynes with quinoxalinones and Langlois' reagent. We envision that GO will facilitate CF_3_SO_2_Na to generate the electrophilic CF_3_ radical intermediate, which enables an intermolecular addition with alkynes to provide nucleophilic alkyl radicals that combine with the C3 position of the quinoxalin-2(1*H*)-ones.

## Results and discussion

The GO material used in this investigation was prepared by Hummers oxidation of graphite and subsequent exfoliation, as reported.^13^ The obtained GO material has been characterized by X-ray photoelectron spectroscopy (XPS), scanning electron microscopy (SEM), and infrared spectrum (IR) analysis (see the ESI†).

Based on our hypothesis, our investigation commenced with the reaction of *N*-methylquinoxalin-2(1*H*)-one (1a), phenylacetylene (2a) and the CF_3_-transfer regent (Langlois' reagent) in the presence of GO (80 wt%) and a mixture of acetonitrile and EA (1 : 1 v/v) at 120 °C. It was observed that the desired product, trifluoroalkylated quinoxalinone (3a) was obtained as an *E*/*Z* isomeric mixture (2.8 : 1) with an isolated yield of 85% (Table 1, entry 1; additional details in Tables S1 and S2 in ESI†). The GO loading-screening results showed that reducing the amount of GO from 80 wt% to 50 wt% dramatically decreased the yield from 85% to 22%, but increasing it further to 100 wt% did not show any negative effect on the yield, suggesting that GO was required only in an 80 wt% loading (entries 2 and 3). Screening of other temperatures including 60 °C (yield 10%) and 140 °C (yield 79%) did not afford any advantage over 120 °C (entries 4 and 5). The use of a single solvent, such as MeCN or EA, led to a very low yield (entries 6 and 7). While changing to other solvents mixed in MeCN, the trifluoromethylation proceeded adversely and the yields diminished (entries 8 and 9). When Ar was used instead of air, the reaction was significantly inhibited (entry 10). The absence of GO failed to afford 3a (entry 11), indicating that this carbocatalyst was essential in the promotion of trifluoromethylation. Ultimately, we found that 1a, 2a and CF_3_SO_2_Na in the presence of 80 wt% GO at 120 °C for 6 hours afforded the desired product 3a with 85% yield (*E*/*Z* = 2.8 : 1, which was determined by the proton NMR of the mixture of *E*/*Z* isomers).

**Table 1:** Optimization of the reaction conditions^a^

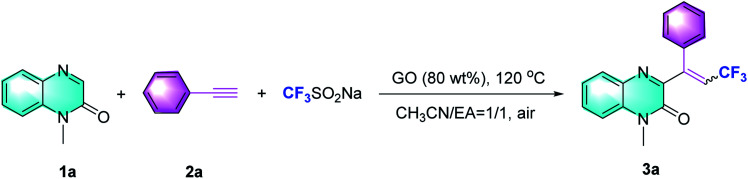		
Entry	Variation of the conditions	Yield^b^ (%)
1	80 wt% GO, 120 °C, MeCN/EA = 1 : 1	85 (*E*/*Z* = 2.8 : 1)
2	50 wt% GO	22 (*E*/*Z* = 2.8 : 1)
3	100 wt% GO	85 (*E*/*Z* = 2.8 : 1)
4	60 °C	10 (*E*/*Z* = 3.5 : 1)
5	140 °C	79 (*E*/*Z* = 2.6 : 1)
6	MeCN	58 (*E*/*Z* = 2.7 : 1)
7	EA	56 (*E*/*Z* = 2.8 : 1)
8	MeCN/THF = 1/1	75 (*E*/*Z* = 2.8 : 1)
9	MeCN/1,4-dioxane = 1/1	68 (*E*/*Z* = 2.6 : 1)
10	Ar	20 (*E*/*Z* = 2.7 : 1)
11	Without GO	0

a Reaction conditions: 1a (0.2 mmol, 1 equiv.), 2a (0.4 mmol, 2 equiv.), Langlois' reagent (0.4 mmol, 2 equiv.), GO (80 wt%), air, 120 °C, and 6 h.

b Isolated yield. EA = ethyl acetate.

Subsequently, the scope of the Csp–CF_3_ bond addition/trifluoromethylation reaction was studied. The influence of substituents on the quinoxalin-2(1*H*)-one moiety is illustrated in Table 2. For the *N*-methylquinoxalinones with substituents at the 5 or 6 position of the benzene ring, the corresponding trifluoromethylated quinoxalin-2(1*H*)-ones 3a–3h were obtained with moderate to good yields (43–85%). Various *N*-substituted quinoxalin-2(1*H*)-ones could react smoothly. Overall, the transformation of *para*-substituted benzylquinoxalinones bearing methyl, *t*-Bu, halogen, ester, CF_3_ and OCF_3_ groups delivered the trifluoromethylated products (3i–3o) with 65–86% yield. Moreover, *N*-alkylquinoxalinones were also compatible with the reaction and provided the corresponding products (3p–3r). Different types of *N*-substitutes, including alkenyl (3s), alkynyl (3t), ester (3u), acetylbenzene (3v) and naphthyl (3w), revealed obvious activities, and the trifluoromethylated products were isolated with satisfactory yields. It is notable that 1-(prop-2-yn-1-yl) quinoxalin-2(1*H*)-one could also be used in the reaction, and the corresponding product (3t) was provided with 65% yield. In the case of the substrate quinoxalin-2(1*H*)-one, it underwent trifluoromethylative addition smoothly, leading to product 3x with 81% yield. Furthermore, the structure of 3p was unambiguously confirmed by X-ray crystallography (CCDC 2088404, see the ESI†).

**Table 2:** Conversion of the prepared quinoxalin-2(1*H*)-ones, phenylacetylene and Langlois' reagent into 3-trifluoromethylquinoxalin-2(1*H*)-ones^a^^,^^b^

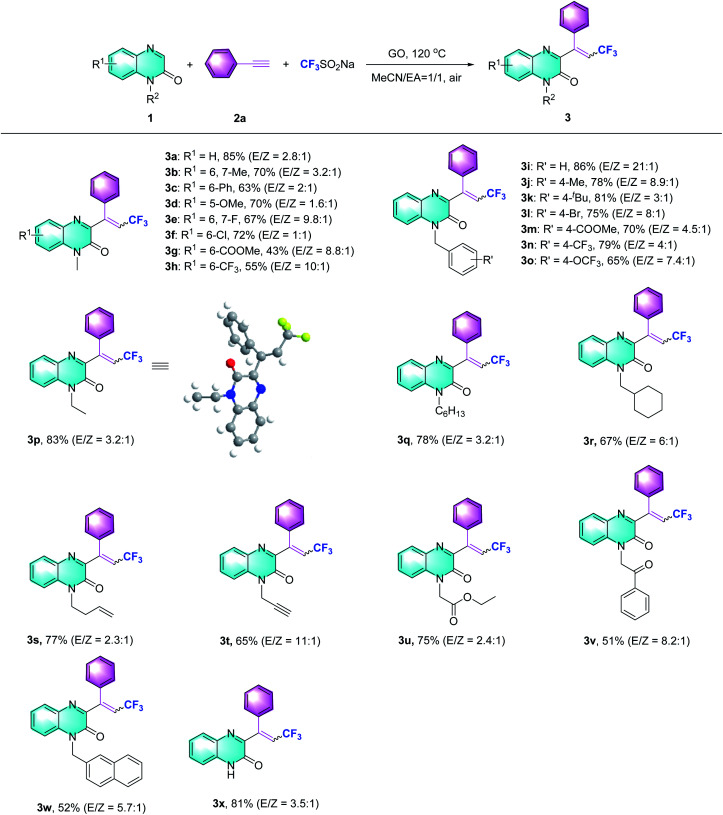

a Reaction conditions: 1 (0.2 mmol, 1 equiv.), 2a (0.4 mmol, 2 equiv.), Langlois' reagent (0.4 mmol, 2 equiv.), GO (80 wt%), air, 120 °C, and 6 h.

b Isolated yield.

After exploring the scope of the quinoxalin-2(1*H*)-one derivatives, we turned our attention to the variation of the system of different alkynes under the optimized conditions (Table 3). Phenylacetylene substrates bearing methyl, methoxy and fluoro groups successfully carried out the Csp–CF_3_ bond addition and afforded the desired products (3y–3aa). When aliphatic alkynes with cyclohexane or cyclopropane substituents were assessed using the present method, moderate to excellent yields were obtained (3ab–3ac). The reaction of other alkyl alkynes could work equally well to produce the desired products with good yields (3ad and 3ae). Regarding alkyl alkynes, such as those with cyano, chloro, hydroxyl, and protected hydroxyl groups, lower reaction efficiencies were generally observed, supplying the trifluoromethylquinoxalin-2(1*H*)-ones with yields ranging from 32% to 63% (3af to 3ai). Besides terminal alkynes, to our delight, this strategy also obeyed the trifluoromethylation of propargylic Csp–CF_3_ bond addition to provide 3aj, ak with 53% and 51% yields, respectively.

**Table 3:** Substrate scope of the Csp–CF_3_ bond addition/trifluoromethylation^a^^,^^b^

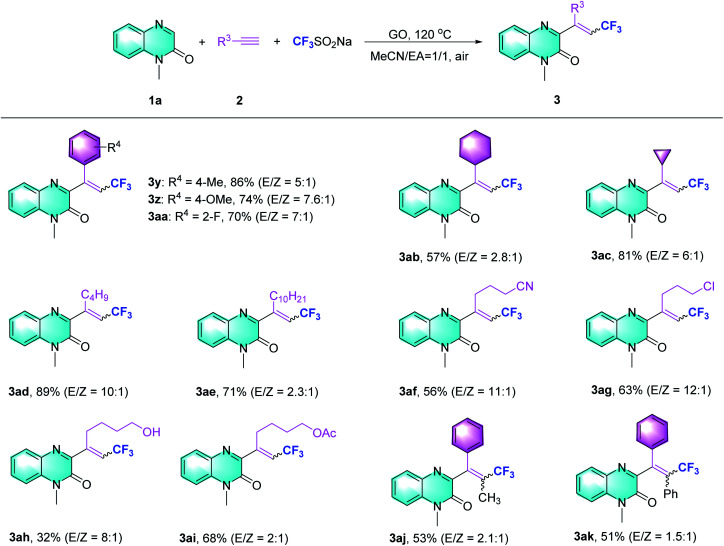

a Reaction conditions: 1a (0.2 mmol, 1 equiv.), 2a (0.4 mmol, 2 equiv.), Langlois' reagent (0.4 mmol, 2 equiv.), GO (80 wt%), air, 120 °C, and 6 h.

b Isolated yield.

**Scheme 2:**
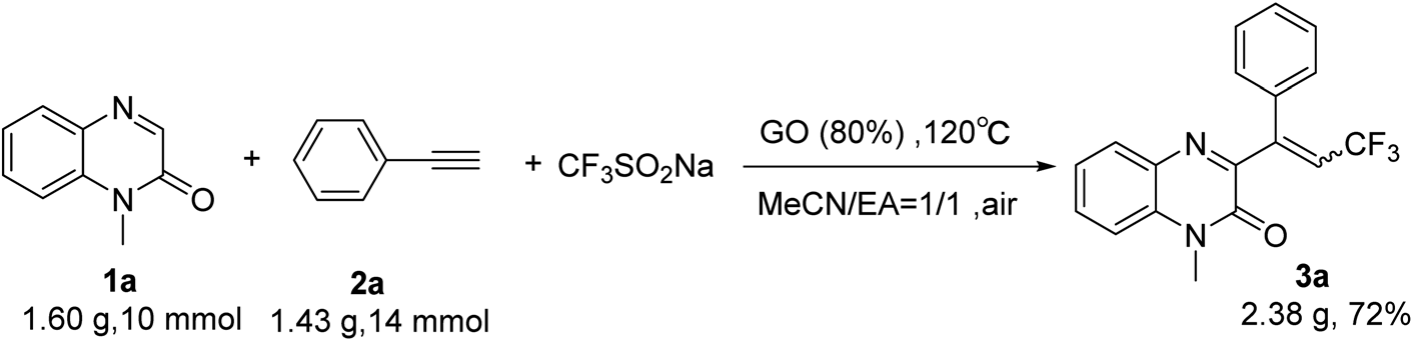
Gram-scale preparation of 3a.

In order to demonstrate the effectiveness of this new strategy, a gram scale reaction was performed under the standard conditions. *N*-Methylquinoxalin-2(1*H*)-one (1a) (10 mmol), phenylacetylene (2a) (20 mmol) and Langlois' reagent (4 mmol) were subjected to the reaction in the presence of GO (1.28 g, 80 wt%) in a mixture of acetonitrile and EA (1 : 1 v/v) (60 mL) at 120 °C. After 6 h, the desired product 3a was obtained with 72% yield, which demonstrated the practical application of this protocol to prepare 3-trifluoroalkylated quinoxalin-2(1*H*)-ones on a gram-scale (Scheme 2).

**Scheme 3:**
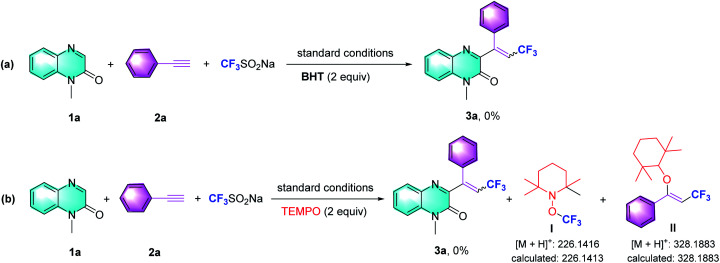
Mechanistic studies.

To gain more insight into these multi-component tandem reactions of nucleophilic addition, we evaluated the effect of the radical scavengers butylated hydroxytoluene (BHT) and 2,2,6,6-tetramethyl-1-piperidinyloxy (TEMPO). As shown in Scheme 3, the reactions were significantly inhibited, indicating that radicals might be involved. In accordance with the literature, we conceived the generation of the trifluoromethyl radical intermediate from Langlois' reagent.^5*b*,*c*,14^ TEMPO was introduced to the optimized reaction conditions and we observed the formation of TEMPO adducts (I and II), which were confirmed by MS analysis (Scheme 3b).

GO with high-oxygen content is usually labile at higher temperatures. X-ray photoelectron spectroscopy (XPS) and Fourier transform infrared (FT-IR) analysis showed that a large number of epoxide groups and carbonyl functional groups are lost when GO participates in the reaction (Fig. S2–S4†). GO and recovered-GO (GO-R) were analyzed by XPS in order to study their surface chemical state and chemical composition. The full-scale XPS spectrum (Fig. S3†) proved that GO has C, O and S elements. For GO-R, the presence of C, O, F and S were confirmed by the survey XPS spectra, indicating that NaSO_2_CF_3_ was successfully doped in the carbon catalyst.

**Scheme 4:**
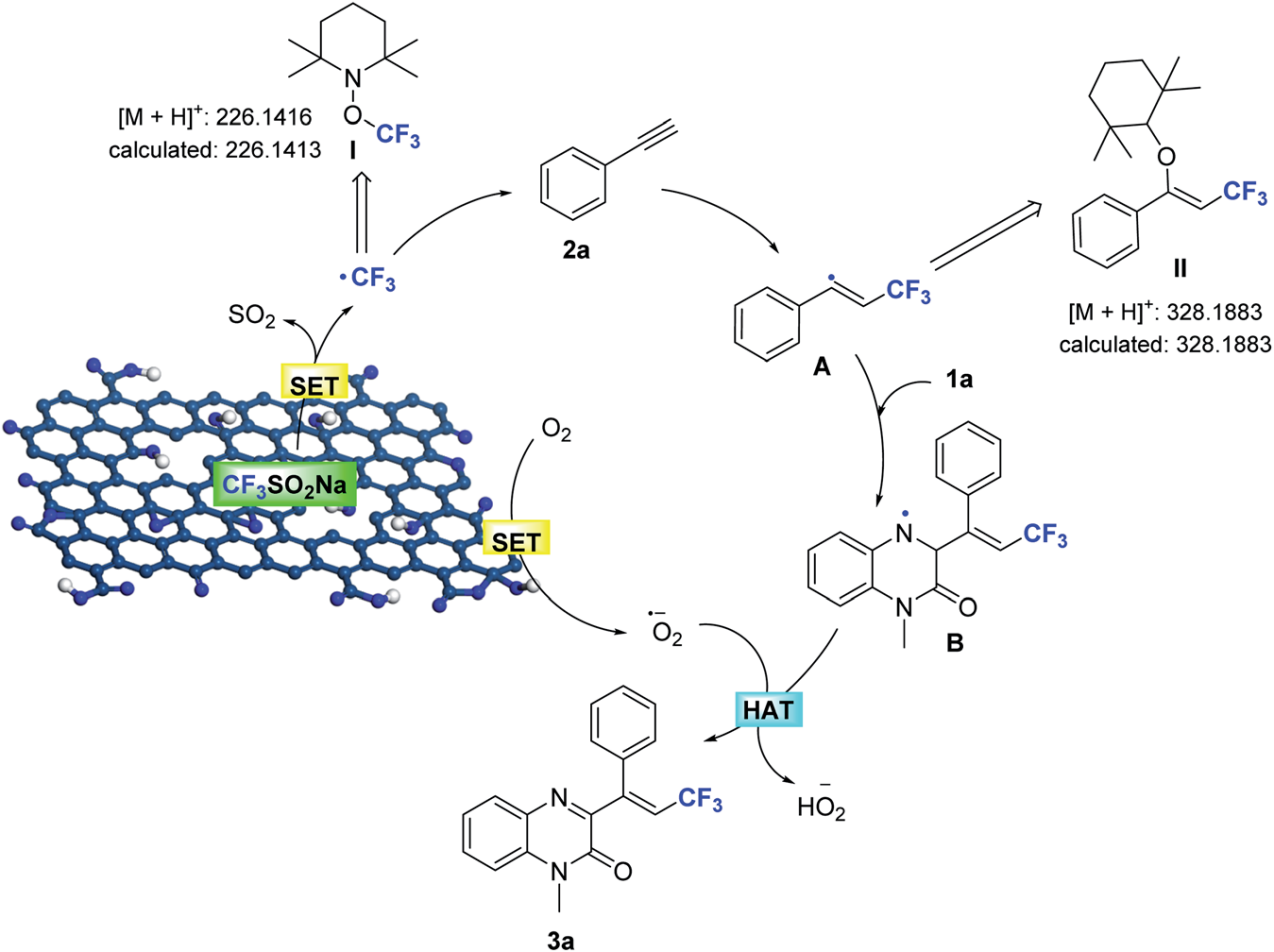
Proposed catalytic cycle for the trifluoromethylation of alkynes.

On the basis of these observations, and the established catalytic activity of graphene oxide,^2,15,16^ a plausible pathway for Csp–CF_3_ bond addition has been outlined in Scheme 4. The edge sites with unpaired electrons and oxygen functional groups of the porous GO shell constitute the active sites, which enhance the formation of the trifluoromethyl radical and the superoxide radical by a tandem single-electron transfer (SET). The initial reaction of NaSO_2_CF_3_ generates the trifluoromethyl radical (detected by MS) in the GO catalytic system by a SET process,^17^ and is subsequently added to the alkyne to afford the *trans*-alkenyl complex A (detected by MS). The *N*-methylquinoxalin-2(1*H*)-one (1a) is immediately trapped into the generated alkenyl radical, resulting in the intermediate *N*-alkylquinoxalinone radical B. The unpaired electrons of GO reduce O_2_ to form O_2_˙^−^,^2*g*,15*b*^ which can abstract hydrogen atoms from radical B to furnish the adduct product 3a. At the same time, the active sites of porous GO are released to maintain the catalytic cycle.

## Conclusions

We have developed a tandem radical addition strategy of an O_2_-assisted one-pot process to synthesize 3-trifluoroalkylated quinoxalin-2(1*H*)-ones under ambient atmosphere and 80 wt% GO content. Mechanistic studies reveal that GO affected the formation of trifluoromethyl and superoxide radicals and was subsequently capable of performing an auto-tandem radical addition. This unique multi-component tandem reaction facilitated access to CF_3_-substituted quinoxalinones by the catalysis of a readily available carbon material in the absence of metal catalysts. In a broader perspective, these transformations also emphasize the potential of GO in synthetic chemistry, particularly in metal-free synthesis, rather than simply using a precursor to graphene-based materials.

## Conflicts of interest

There are no conflicts to declare.

## Supplementary Material

RA-011-D1RA07014B-s001

RA-011-D1RA07014B-s002
