# Supplementary material for: Graphene oxide-catalyzed trifluoromethylation of alkynes with quinoxalinones and Langlois' reagent
Source: RSC Adv. 2021 Dec 1;11(61):38667–73. doi: 10.1039/d1ra07014b (PMC9044184; doi:10.1039/d1ra07014b)

Electronic Supplementary Material (ESI) for RSC Advances.
This journal is © The Royal Society of Chemistry 2021

Supporting Information for

Graphene Oxide-Catalyzed Trifluoromethylation of Alkynes with Quinoxalinones and Langlois' Reagent

Hong Li,[a] Xiangjun Peng,*[b] Liang Nie,[a] Lin Zhou,[a] Ming Yang[a], Fan Li,[a] Jian Hu,[b] Zhiyang Yao,[b] Liangxian Liu*[a]

[a] *Key Laboratory of Organo-Pharmaceutical Chemistry of Jiangxi Province, Gannan Normal University, Ganzhou, Jiangxi 341000, P. R. China*

[b] *Key Laboratory of Prevention and Treatment of Cardiovascular and Cerebrovascular Diseases of Ministry of Education, Gannan Medical University, Ganzhou 341000, P. R. China*

E-mail: liuliangxian1962@163.com

CONTENTS

1. General Methods……………...……………………………………………… …………2
2. Experimental Procedures..3
3. Characterization of graphene oxide (GO)..……………………….……………..................5
4. Control Experiments..8
5. The X-ray Crystal Structure of **3p**...9
6. ^{1}H, ^{19}F, ^{13}C NMR, MP and MS Data of All products...10
7. References...23
8. Copies of ^{1}H NMR, ^{19}F NMR and ^{13}C NMR Spectra for All Compounds.................................24

1. General Methods

Unless otherwise specified, commercial reagents and solvents were used without further purification. Commercially available chemicals were purchased from Shanghai Haohong Scientific Co., Ltd. (Leyan) and used without any further purification. ^{1}H and ^{13}C NMR spectra were recorded on a Bruker spectrometers at 400 and 100 MHz, respectively. LC-electrospray ionization mass spectra were were recorded with Bruker Dalton Esquire 3000 plus LC-MS apparatus. Elemental analysis were carried out on a Perkin-Elmer 240B instrument. HRFABMS spectra were recorded on a FTMS apparatus. Silica gel (300-400 mesh) was used for flash column chromatography, eluting (unless otherwise stated) with an ethyl acetate/petroleum ether (PE) (60-90 ℃) mixture.

The graphene oxide sample was recorded with Zeiss Sigma 300. Fourier transform infrared spectroscopy of graphene oxide recorded on a Jasco ATR MIRacle spectrophotometer. Samples were scanned in the 400-4000cm^{-1} region with KBr pellet. X-ray photoelectron spectroscopy recorded with Thermo Fisher Scientific K-Alpha using Al K_α radiation (ħω =1253.6 eV). The X-ray power was 125 W. The spectra were recorded in the constant analyzer energy (CAE) mode with analyzer pass energies of 50 eV for the high resolution spectra. Charging effects were corrected by energy calibration on Al level relative to 284.80 eV. Compound **3p** were collected by a diffractometer Rigaku Oxford Diffraction Supernova Dual Source, Cu at Zero equipped with an AtlasS2 CCD using Cu Kα radiation (1.54178 Å) by using a w scan mode.

2. Experimental Procedures

2.1 General Procedure of the Products 3

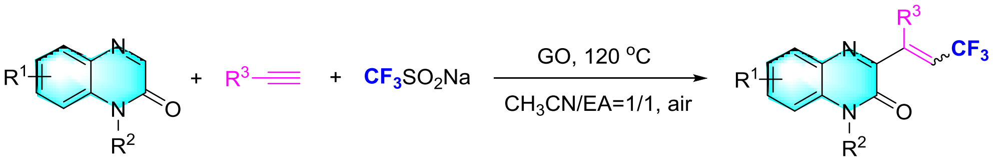

1 2 3

To a mixed solvent of MeCN and EA (1:1 v/v) of quinoxalone **1** (0.2 mmol), CF_3SO_2Na (0.4 mmol), and GO (80 wt%) was added alkyne **2** (0.4 mmol) under an air atmosphere and the mixture was stirred at 120 ℃ for 6 h. The reaction mixture was concentrated under reduced pressure. The residue was purified by flash chromatography on silica gel (eluent: EA/PE = 1:4) to yield the corresponding product **3**.

2.2 Table S1. Optimization Study of CF_3-substituted alkoxyquinoxalones[a]

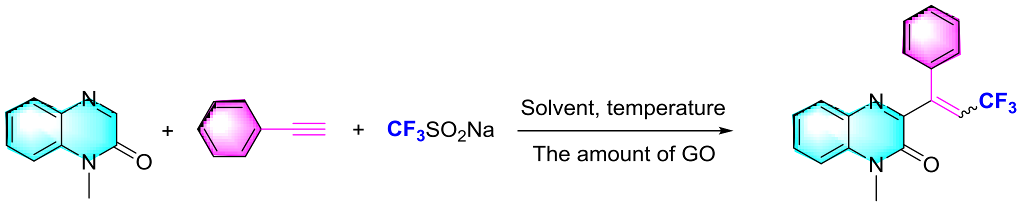

1a 2a 3a

Entry	solvent	Temperature	The amount of GO	Yield(%)[b]
1	EA	100 ℃	100 wt%	56
2	Acetone	100 ℃	100 wt%	25
3	DCE	100 ℃	100 wt%	48
4	DCM	100 ℃	100 wt%	45
5	MeCN	100 ℃	100 wt%	58
6	H_2O	100 ℃	100 wt%	N.R.
7	DMF	100 ℃	100 wt%	12
8	DMSO	100 ℃	100 wt%	10
9	MeOH	100 ℃	100 wt%	26
10	THF	100 ℃	100 wt%	56
11	1,4-dioxane	100 ℃	100 wt%	58
12	MeCN/THF=1:1	100 ℃	100 wt%	75
13	MeCN/1,4-dioxane =1:1	100 ℃	100 wt%	68
14	MeCN/EA=1:1	100 ℃	100 wt%	78
15	MeCN/EA=1:1	60 ℃	100 wt%	10
17	MeCN/EA=1:1	120 ℃	100 wt%	85
18	MeCN/EA=1:1	140 ℃	100 wt%	79
19	MeCN/EA=1:1	120 ℃	50 wt%	22
**20**	**MeCN/EA=1:1**	**120 ℃**	**80 wt%**	**85**
21	MeCN/EA=1:1	120 ℃	150 wt%	82

22	MeCN/EA=1:1	120 ℃	200 wt%	85
23[c]	MeCN/EA=1:1	120 ℃	80 wt%	20
24[d]	MeCN/EA=1:1	120 ℃	80 wt%	85
25	MeCN/EA=1:1	120 ℃	–	NR

[a]Reaction conditions: **1a** (0.2 mmol, 1 equiv), **2a** (0.4 mmol, 2 equiv), Langlois' reagent (0.4 mmol, 2 equiv), GO, air, temperature, and 6 h. [b]Isolated yield. EA = ethyl acetate. [c]Ar. [d]O_2.

3. Characterization of graphene oxide (GO)

3.1 The scanning electron microscope of GO and GO recovered (GO-R)

GO was prepared by graphite oxidation using the Hummers and Offeman method and subsequent exfoliation.[1] Considering the π-π interactions and oxygen-containing functional groups could facilitate the mass transfer and then increase catalytic performance, scanning electron microscope (SEM), Fourier Transform infrared spectroscopy (FT-IR) and X-ray photoelectron spectroscopy (XPS) were used to analyze and characterize this inorganic-organic hybrid material. The SEM image of the sample, a black flaky and blocky structure, was clearly observed that the GO has 1 μm in Figure S1. The surface of GO showed a classic folded morphology,[2] and its specific surface area was large, providing an ideal catalytic surface for the introduction of active centers (Figure S1 A). After the reaction completed and GO recovered (GO-R), the fold surface was decreased and damaged (Figure S1 B).

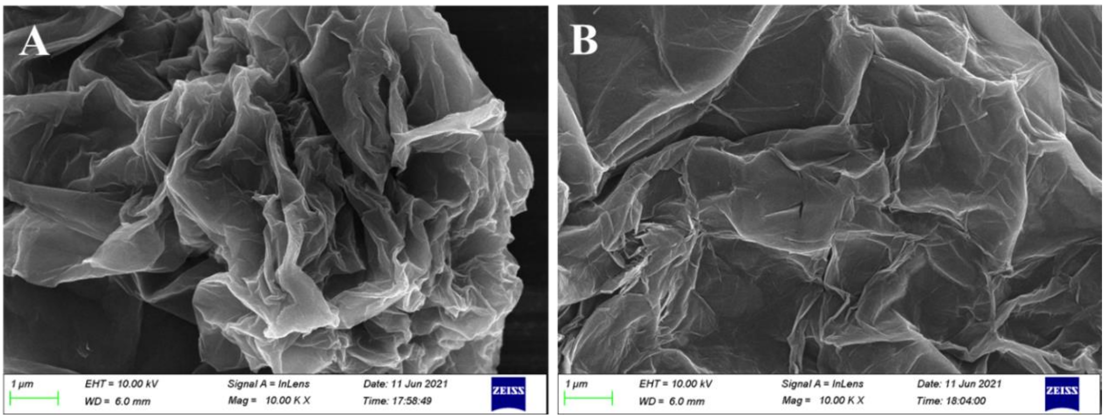

Figure S1. SEM image of (A) freshly prepared GO and (B) after recycling of GO-R from the trifluoromethylation reaction.

3.2 Fourier transform infrared (FT-IR) spectroscopy of GO and GO-R

The FT-IR spectroscopy analysis was further carried out. As shown in Figure S1(1), for the GO, the wide peak at 3600-1900 cm^{-1} could be ascribed to the vibration deformation and vibration stretching of hydroxyl (O-H). The peak of 1721 cm^{-1} was vibration stretching absorption of carbonyl group (C=O), and the wide peak at 1483-890 cm^{-1} was the vibration stretching of epoxy group (C-O) and vibration stretching of alkoxy group (C-O) at the surface of GO.[3] The peak at 1618 cm^{-1} was the vibration deformation peak of water. The above FT-IR data indicated that GO surface was rich in hydroxyl, carbonyl, epoxide, ether and other oxygen-containing functional groups. After recycling of GO-R from the trifluoromethylation reaction, a new peak at 1562 cm^{-1} emerged in the spectrum of GO-R, which could be attributed to the characteristic bands of amide (νCN + δNH).[4] Compared with fresh GO, the range of recovered heterogeneous GO-R became narrow at the wide peaks of hydroxyl (O-H) and epoxy group (C-O), indicating that the cooperative action of the oxygen-containing moieties, Brønsted acidity and π-surface of the GO was crucial in the reaction course of the trifluoromethylation protocol.

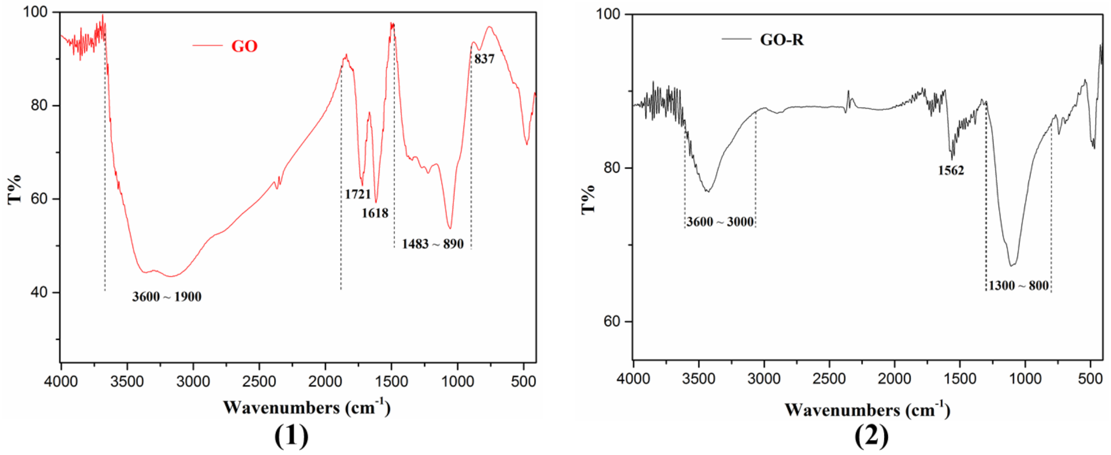

Figure S2. The FT-IR spectrum of (1) GO and (2) GO-R.

3.3 The XPS spectra of GO and GO-R

The samples were analyzed by XPS in order to study the surface chemical state and chemical composition of GO and GO-R. The full-scale XPS spectrum (Figure S3) proved that GO have C, O and S elements. For GO-R, the presence of C, O, F and S were confirmed by survey XPS spectra, indicating that $NaSO_2CF_3$ was successfully doped in carbon catalyst.

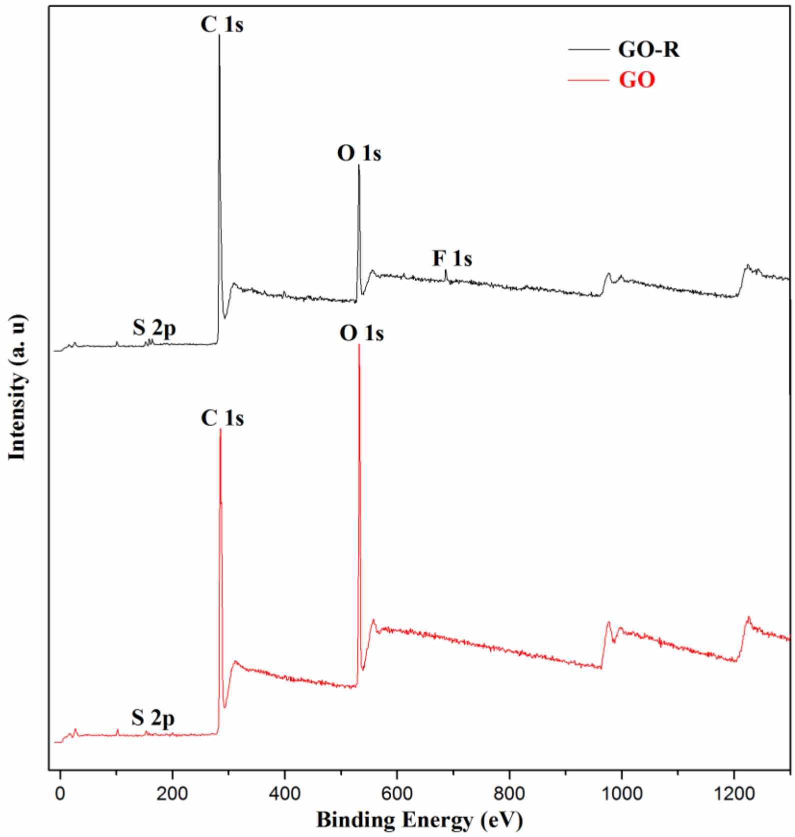

Figure S3. XPS Survey spectra of GO and GO-R.

The high-resolution spectra were given in Figure S4 to clarify the bond configurations of GO and GO-R. For both GO and GO-R, four characteristic peaks at about 284, 530, 680 and 160 eV appeared, which belonged to C 1s, O 1s, F 1s, and S 2p, respectively.

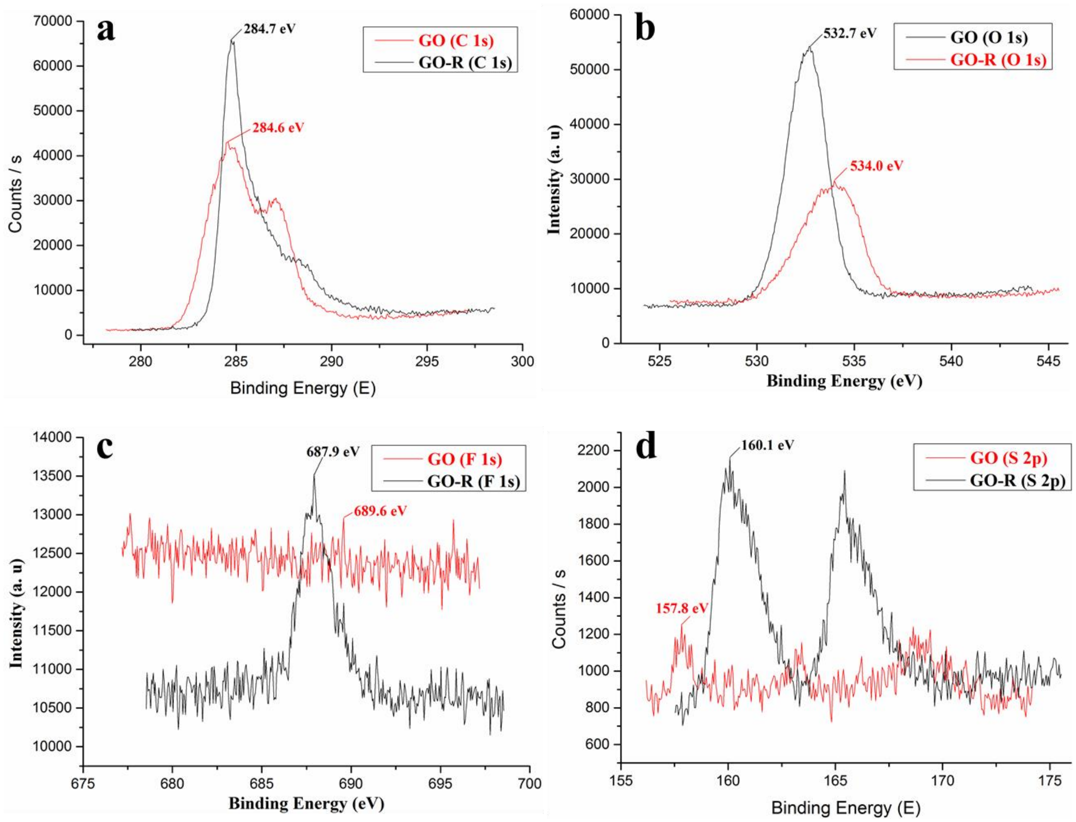

Figure S4. GO and GO-R XPS spectra of (a) C 1s, (b) O 1s, (c) F 1s and (d) S 2p.

XPS data also supported the conclusion that oxygen containing functional groups were slowly draining away in the recovered GO. As shown in Table S2, the percentage of C was increased substantially to 49.45% from 72.06%, while the O content was decreased to 20.22% from 47.72% suggesting that the oxygen containing functional groups of GO were consumed concomitantly to the reaction course, and aromatic carbons were not affected by the reaction. In addition, the S and F elements increased from 0.91% to 3.03% and 1.92% to 4.68%, respectively.

Table S2. Summary of elemental analysis of GO and recovered GO catalyst by XPS measurement.

Catalyst	C%	O%	S%	F%
GO	49.45	47.72	0.91	1.92
Recovered GO	72.06	20.22	3.03	4.68

4. Control Experiments

4.1 Control Experiments from the trifluoromethylation reaction

Some control experiments were performed to further investigate the mechanism of the trifluoromethylation reaction (Figure S5). 2,6-Di-tert-butyl-4-methylphenol (BHT) or 2,2,6,6-tetramethylpiperidinooxy (TEMPO) was added to the reaction system, and the yield of **3a** obviously decreased, which suggested the reaction could undergo a radical process.

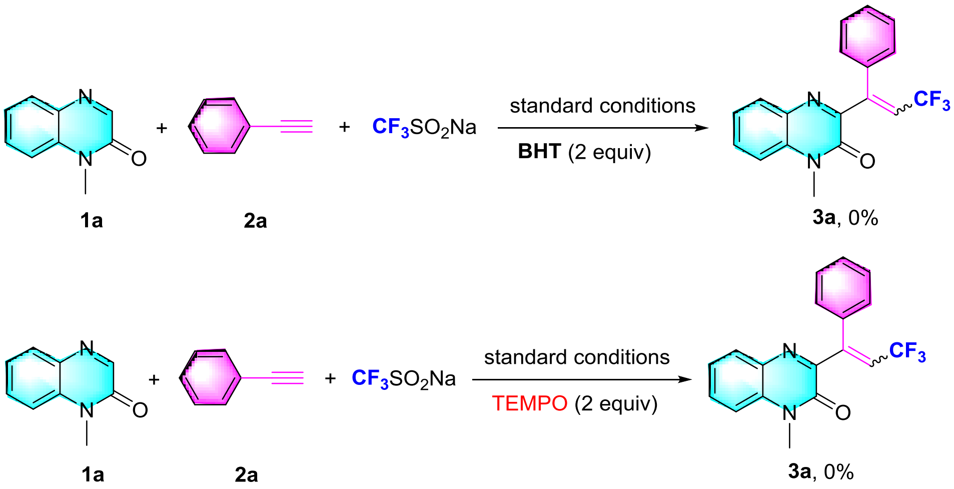

Figure S5. Control experiments.

4.2 Detection of intermediates from the reaction by ESI-HRMS

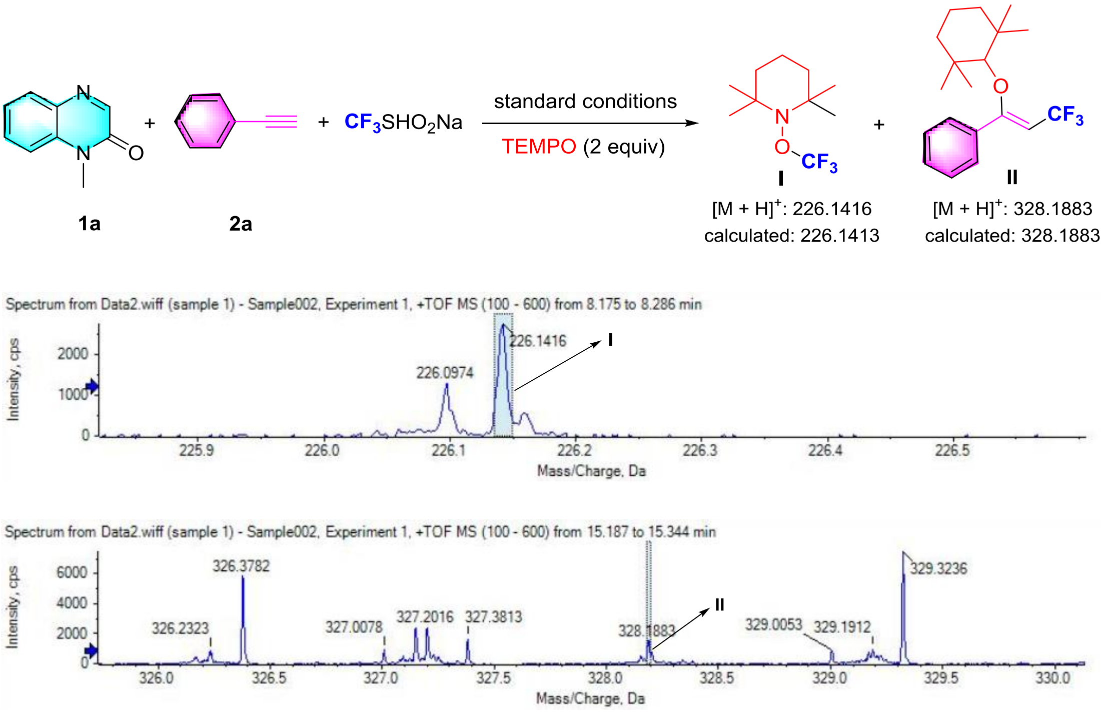

Figure S6. [a]Reaction conditions: **1a** (0.2 mmol, 1 equiv), **2a** (0.4 mmol, 2 equiv), Langlois' reagent (0.4 mmol, 2 equiv), GO (80 wt%), air, 120 ℃, and 30 min. [b]Isolated yield.

5. The X-ray Crystal Structure of 3p

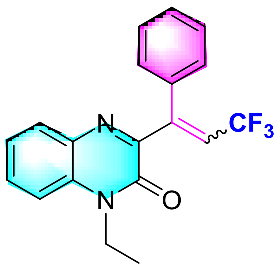

3p
CCDC 2088404

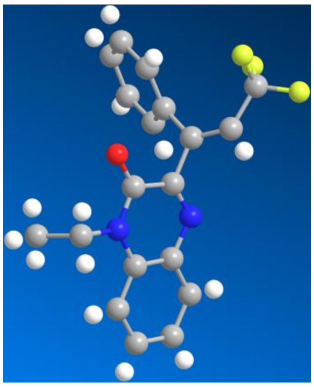

Table S3. Crystal Data and Structure Refinement for Complex 3p.

```
Bond precision:   C-C = 0.0043 A          Wavelength=0.71076

Cell:            a=12.2095(6)    b=12.2629(6)          c=12.3037(6)
                 alpha=90        beta=118.7457(17)     gamma=90
Temperature:     293 K

                 Calculated                  Reported
Volume           1615.14(14)                 1615.13(14)
Space group      P 21/n                      P 1 21/n 1
Hall group       -P 2yn                      -P 2yn
Moiety formula   C19 H15 F3 N2 O             C19 H15 F3 N2 O
Sum formula      C19 H15 F3 N2 O             C19 H15 F3 N2 O
Mr               344.33                      344.33
Dx,g cm-3        1.416                       1.416
Z                4                           4
Mu (mm-1)        0.112                       0.112
F000             712.0                       712.0
F000'            712.43
h,k,lmax         15,15,15                    15,15,15
Nref             3719                        3703
Tmin,Tmax        0.972,0.975                 0.972,0.975
Tmin'            0.972

Correction method= # Reported T Limits: Tmin=0.972 Tmax=0.975
AbsCorr = MULTI-SCAN

Data completeness= 0.996          Theta(max)= 27.512

R(reflections)= 0.0713( 2475)     wR2(reflections)= 0.1474( 3703)

S = 1.097                    Npar= 227
```

6. ^{1}H, ^{19}F, ^{13}C NMR, MP and MS Data of All products

1-Methyl-3-(3,3,3-trifluoro-1-phenylprop-1-en-1-yl)quinoxalin-2(1*H*)-one (**3a**)

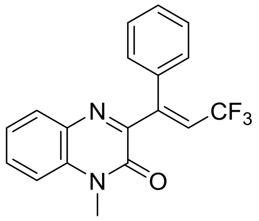

Yellow solid (56 mg, 85%). Mp: 82-83 ℃. ^{1}H NMR (400 MHz, CDCl$_3$): δ 7.89 (dd, J = 8.0, 1.4 Hz, 1H), 7.63 (dt, J = 1.4, 7.4 Hz, 1H), 7.42-7.37 (m, 6H), 7.34 (d, J = 8.4 Hz, 1H), 6.84 (q, J = 8.3 Hz, 1H), 3.69 (s, 3H). ^{13}C NMR (100 MHz, CDCl$_3$): δ 154.5, 153.8, 148.7 (q, J = 5.6 Hz), 135.1, 133.6, 132.5, 131.5, 131.0, 128.6 (q, J = 1.8 Hz), 128.5, 127.8, 124.0, 122.7 (q, J = 34.3 Hz), 122.6 (q, J = 271.7 Hz), 113.7, 29.2. ^{19}F NMR (376 MHz, CDCl$_3$) δ -56.25; HRESIMS calcd for $[C_{18}H_{13}F_3N_2O + H]^+$ 331.1058, found 331.1068.

(*E*)-1,6,7-Trimethyl-3-(3,3,3-trifluoro-1-phenylprop-1-en-1-yl)quinoxalin-2(1*H*)-one (**3b**)

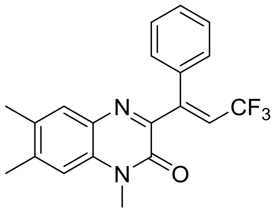

Yellow oil (50 mg, 70%). ^{1}H NMR (400 MHz, CDCl$_3$): δ 7.75 (s, 1H), 7.46 (d, J = 7.1 Hz, 1H), 7.45 (d, J = 7.8 Hz, 1H), 7.40-7.35 (m, 3H), 7.16 (s, 1H), 6.29 (q, J = 8.1 Hz, 1H), 3.70 (s, 3H), 2.48 (s, 3H), 2.40 (s, 3H). ^{13}C NMR (100 MHz, CDCl$_3$): δ 153.7, 153.6, 147.4, 141.4 (q, J = 5.3 Hz), 136.4, 133.1, 131.7, 131.0, 130.8, 129.7, 128.8, 126.9, 122.8 (q, J = 270.8 Hz), 118.2 (q, J = 34.0 Hz), 114.4, 29.1, 20.7, 19.2. ^{19}F NMR (376 MHz, CDCl$_3$) δ -58.28; HRESIMS calcd for $[C_{20}H_{17}F_3N_2O + H]^+$ 359.1371, found 359.1384.

(*E*)-1-Methyl-6-phenyl-3-(3,3,3-trifluoro-1-phenylprop-1-en-1-yl)quinoxalin-2(1*H*)-one (**3c**)

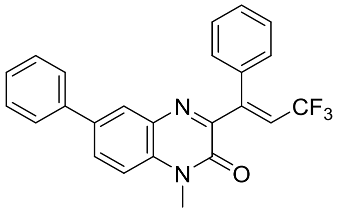

Yellow solid (51 mg, 63%). Mp: 120-121 ℃. ^{1}H NMR (400 MHz, CDCl$_3$): δ 7.94 (d, J = 8.3 Hz, 1H), 7.69 (dd, J = 8.5, 1.4 Hz, 2H), 7.62 (dd, J = 8.3, 1.7 Hz, 1H), 7.54 (t, J = 7.4 Hz, 2H), 7.48 (dd, J = 7.6, 1.5 Hz, 2H), 7.45-7.39 (m, 5H), 6.89 (q, J = 8.3 Hz, 1H), 3.75 (s, 3H). ^{13}C NMR (100 MHz, CDCl$_3$): δ 154.0, 153.9, 148.7 (q, J = 5.5 Hz), 144.8, 139.8, 135.2, 133.9, 131.8, 131.3, 129.2, 128.7 (q, J = 1.7 Hz), 128.6, 128.5, 127.8, 127.5, 123.3, 122.8 (q, J = 271.7 Hz), 122.5 (q, J = 34.2 Hz), 112.0, 29.2. ^{19}F NMR (376 MHz, CDCl$_3$) δ -56.19; HRESIMS calcd for $[C_{24}H_{17}F_3N_2O + H]^+$ 407.1371, found 407.1372.

(*E*)-5-Methoxy-1-methyl-3-(3,3,3-trifluoro-1-phenylprop-1-en-1-yl)quinoxalin-2(1*H*)-one (**3d**)

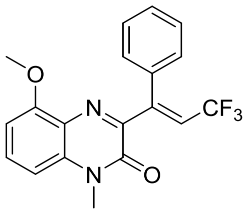

Yellow oil (50 mg, 70%). ^{1}H NMR (400 MHz, $CDCl_3$): δ 7.60 (dd, *J* = 8.1, 1.4 Hz, 1H), 7.46 (dd, *J* = 5.1, 1.8 Hz, 2H), 7.43-7.37 (m, 3H), 7.33 (t, *J* = 8.1 Hz, 1H), 7.15 (dd, *J* = 8.1 Hz, 1H), 6.29 (q, *J* = 8.1 Hz, 1H), 4.01 (s, 3H), 3.96 (s, 3H). ^{13}C NMR (100 MHz, $CDCl_3$): δ 154.9, 154.8, 148.3, 147.1 (q, *J* = 5.5 Hz), 136.2, 134.4, 129.8, 128.9, 126.9, 124.7, 123.9, 123.6, 122.8 (q, *J* = 270.8 Hz), 118.3 (q, *J* = 34.2 Hz), 113.8, 56.7, 34.8. ^{19}F NMR (376 MHz, $CDCl_3$) δ -58.31; HRESIMS calcd for $[C_{19}H_{15}F_3N_2O_2 + H]^+$ 361.1164, found 361.1177.

(*E*)-6,7-Difluoro-1-methyl-3-(3,3,3-trifluoro-1-phenylprop-1-en-1-yl)quinoxalin-2(1*H*)-one (**3e**)

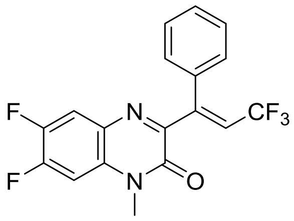

Yellow oil (48 mg, 67%). ^{1}H NMR (400 MHz, $CDCl_3$): δ 7.68 (dd, *J* = 10.0, 8.2 Hz, 1H), 7.42-7.34 (m, 5H), 7.13 (dd, *J* = 11.2, 7.0 Hz, 1H), 6.92 (q, *J* = 8.2 Hz, 1H), 3.64 (s, 3H). ^{13}C NMR (100 MHz, $CDCl_3$): δ 154.5 (d, *J* = 3.8 Hz), 153.4, 152.4 (d, *J* = 256.0 Hz), 152.3 (d, *J* = 256.0 Hz), 148.0 (q, *J* = 5.6 Hz), 145.7 (d, *J* = 14.1 Hz), 134.8, 128.7, 128.6, 128.5, 127.9, 123.2 (q, *J* = 34.3 Hz), 122.6 (q, *J* = 271.7 Hz), 118.5 (dd, *J* = 18.0, 2.3 Hz), 102.4 (d, *J* = 23.2 Hz), 29.8. ^{19}F NMR (376 MHz, $CDCl_3$) δ -56.34, -127.92 (d, *J* = 22.3 Hz), -141.00 (d, *J* = 22.3 Hz); HRESIMS calcd for $[C_{18}H_{11}F_5N_2O + H]^+$ 367.0870, found 367.0883.

(*Z*/*E*)-6-Chloro-1-methyl-3-(3,3,3-trifluoro-1-phenylprop-1-en-1-yl)quinoxalin-2(1*H*)-one (**3f**)

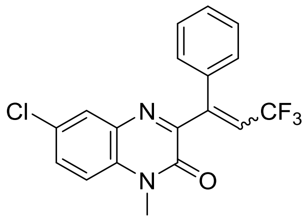

Inseparable yellow oil (52 mg, combined yield: 72%). ^{1}H NMR (400 MHz, $CDCl_3$): δ Z + E 7.99 (d, *J* = 2.4 Hz, 1H), 7.85 (d, *J* = 2.3 Hz, 1H), 7.60 (dd, *J* = 9.0, 2.4 Hz, 1H), 7.55 (dd, *J* = 9.0, 2.4 Hz, 1H), 7.45 (dd, *J* = 7.1, 1.7 Hz, 1H), 7.44 (d, *J* = 2.4 Hz, 1H), 7.40-7.37 (m, 8H), 7.32 (d, *J* = 9.0 Hz, 1H), 7.26 (d, *J* = 9.0 Hz, 1H), 6.94 (q, *J* = 8.2 Hz, 1H), 6.30 (q, *J* = 8.1 Hz, 1H), 3.69 (s, 3H), 3.66 (s, 3H). ^{13}C NMR (100 MHz, $CDCl_3$): δ Z + E 156.4, 155.4, 153.5, 153.3, 148.2 (q, *J* = 5.7 Hz), 146.7 (q, *J* = 5.5 Hz), 135.9, 134.9, 133.0, 132.9, 132.3, 132.2, 131.5, 131.3, 130.2, 130.1, 129.9, 129.4, 129.3, 128.9, 128.7 (q, *J* = 1.7 Hz), 128.6, 127.9, 126.9, 124.0, 123.1 (q, *J* = 34.2 Hz), 121.3, 118.5 (q, *J* = 34.3 Hz), 115.2, 114.9, 29.5, 29.4. ^{19}F NMR (376 MHz, $CDCl_3$) δ Z + E

-56.29, -58.31; HRESIMS calcd for $[C_{18}H_{12}ClF_3N_2O + H]^+$ 365.0669, found 365.0682.

(*E*)-Methyl 1-methyl-2-oxo-3-(3,3,3-trifluoro-1-phenylprop-1-en-1-yl)-1,2-dihydro quinoxaline-6-carboxylate (**3g**)

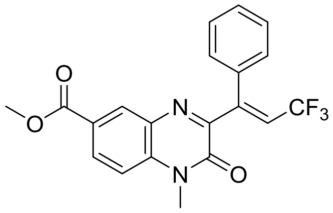

Yellow oil (33 mg, 43%). ^{1}H NMR (400 MHz, $CDCl_3$): δ 8.53 (d, J = 1.9 Hz, 1H), 8.26 (dd, J = 8.8, 2.0 Hz, 1H), 7.42-7.35 (m, 6H), 6.93 (q, J = 8.2 Hz, 1H), 3.97 (s, 3H), 3.71 (s, 3H). ^{13}C NMR (100 MHz, $CDCl_3$): δ 165.8, 155.0, 153.7, 148.1 (q, J = 5.7 Hz), 136.7, 134.8, 132.8, 132.1, 131.8, 128.7 (q, J = 1.7 Hz), 128.6, 127.9, 125.9, 123.1 (q, J = 34.3 Hz), 122.6 (q, J = 271.8 Hz), 113.8, 52.5, 29.5. ^{19}F NMR (376 MHz, $CDCl_3$) δ -58.41; HRESIMS calcd for $[C_{20}H_{15}F_3N_2O_3 + H]^+$ 389.1113, found 389.1118.

(*E*)-1-Methyl-3-(3,3,3-trifluoro-1-phenylprop-1-en-1-yl)-6-(trifluoromethyl)quinoxalin-2(1*H*)-one (**3h**)

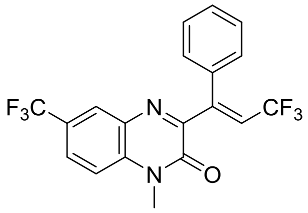

Yellow oil (44 mg, 55%). ^{1}H NMR (400 MHz, $CDCl_3$): δ 8.16 (d, J = 1.3 Hz, 1H), 7.84 (dd, J = 8.8, 1.8 Hz, 1H), 7.45 (d, J = 8.8 Hz, 1H), 7.43-7.38 (m, 5H), 6.95 (q, J = 8.2 Hz, 1H), 3.72 (s, 3H). ^{13}C NMR (100 MHz, $CDCl_3$): δ 155.7, 153.7, 148.0 (q, J = 5.6 Hz), 135.8, 134.7, 131.7, 128.7, 128.5 (q, J = 3.9 Hz), 128.0, 127.9, 127.7 (q, J = 3.6 Hz), 126.3 (q, J = 33.8 Hz), 123.6 (q, J = 272.0 Hz), 123.4 (q, J = 34.3 Hz), 122.6 (q, J = 271.7 Hz), 114.5, 29.5. ^{19}F NMR (376 MHz, $CDCl_3$) δ -56.43, -62.09; HRESIMS calcd for $[C_{19}H_{12}F_6N_2O + H]^+$ 399.0932, found 399.0938.

(*E*)-1-Benzyl-3-(3,3,3-trifluoro-1-phenylprop-1-en-1-yl)quinoxalin-2(1*H*)-one (**3i**)

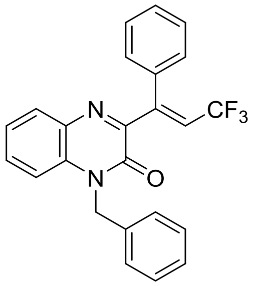

Yellow oil (70 mg, 86%). ^{1}H NMR (400 MHz, $CDCl_3$): δ 7.90 (dd, J = 8.0, 1.4 Hz, 1H), 7.51 (dt, J = 1.5, 8.6 Hz, 1H), 7.50-7.41 (m, 5H), 7.38-7.29 (m, 5H), 7.26 (d, J = 1.3 Hz, 1H), 7.25 (d, J = 8.2 Hz, 1H), 7.07 (q, J = 8.4 Hz, 1H), 5.53 (d, J = 15.9 Hz, 2H). ^{13}C NMR (100 MHz, $CDCl_3$): δ 154.3, 154.0, 148.6 (q, J = 5.6 Hz), 135.2, 134.9, 132.9, 132.7, 131.6, 131.2, 129.0, 128.8 (q, J =

1.2 Hz), 128.6, 127.9, 127.8, 126.9, 124.1, 122.9 (q, *J* = 34.2 Hz), 121.5, 114.5, 46.1. ^{19}F NMR (376 MHz, $CDCl_3$) *δ* -56.05; HRESIMS calcd for $[C_{24}H_{17}F_3N_2O + H]^+$ 407.1371, found 407.1387.

(*E*)-1-(4-Methylbenzyl)-3-(3,3,3-trifluoro-1-phenylprop-1-en-1-yl)quinoxalin-2(1*H*)-one (**3j**)

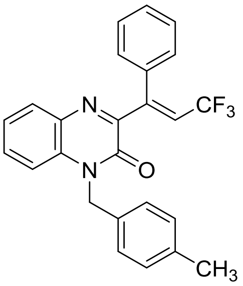

Yellow solid (65 mg, 78%). Mp: 118-119 ℃. ^{1}H NMR (400 MHz, $CDCl_3$): *δ* 7.87 (dd, *J* = 8.6, 1.6 Hz, 1H), 7.50 (dt, *J* = 1.5, 8.4 Hz, 1H), 7.46-7.38 (m, 5H), 7.33 (dt, *J* = 1.1, 8.6 Hz, 2H), 7.13 (s, 4H), 7.02 (q, *J* = 8.3 Hz, 1H), 5.47 (d, *J* = 16.3 Hz, 2H), 2.33 (s, 3H). ^{13}C NMR (100 MHz, $CDCl_3$): *δ* 154.3, 154.0, 148.5 (q, *J* = 5.6 Hz), 137.6, 135.2, 133.0, 132.7, 131.9, 131.5, 131.1, 129.7, 128.7 (q, *J* = 1.2 Hz), 128.5, 127.9, 126.9, 124.0, 122.8 (q, *J* = 34.2 Hz), 122.7 (q, *J* = 271.7 Hz), 114.5, 45.9, 21.1. ^{19}F NMR (376 MHz, $CDCl_3$) *δ* -56.14; HRESIMS calcd for $[C_{25}H_{19}F_3N_2O + H]^+$ 421.1528, found 421.1540 .

(*E*)-1-(4-(*tert*-Butyl)benzyl)-3-(3,3,3-trifluoro-1-phenylprop-1-en-1-yl)quinoxalin-2(1*H*)-one (**3k**)

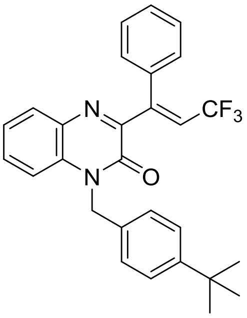

Yellow oil (75 mg, 81%). ^{1}H NMR (400 MHz, $CDCl_3$): *δ* 7.87 (dd, *J* = 7.0, 1.4 Hz, 1H), 7.53 (dt, *J* = 1.5, 8.6 Hz, 1H), 7.46-7.40 (m, 5H), 7.37 (s, 2H), 7.34 (s, 2H), 7.18 (d, *J* = 8.7 Hz, 2H), 7.03 (q, *J* = 8.3 Hz, 1H), 5.48 (d, *J* = 16.2 Hz, 2H), 1.31 (s, 9H). ^{13}C NMR (100 MHz, $CDCl_3$): *δ* 154.3, 154.0, 150.8, 148.5 (q, *J* = 5.7 Hz), 135.2, 133.0, 132.7, 131.8, 131.5, 131.1, 132.8 (q, *J* = 1.7 Hz), 128.5, 127.8, 126.7, 125.9, 124.0, 122.8 (q, *J* = 271.6 Hz), 122.6 (q, *J* = 34.1 Hz), 114.6, 45.8, 34.5, 31.3. ^{19}F NMR (376 MHz, $CDCl_3$) *δ* -56.18; HRESIMS calcd for $[C_{28}H_{25}F_3N_2O + H]^+$ 463.1997, found 463.2018.

1-(4-Bromobenzyl)-3-(3,3,3-trifluoro-1-phenylprop-1-en-1-yl)quinoxalin-2(1*H*)-one (**3l**)

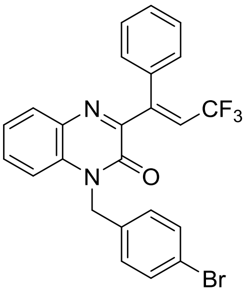

Yellow solid (73 mg, 75%). Mp: 88-89 ℃. ^{1}H NMR (400 MHz, $CDCl_3$): δ 7.89 (dd, J = 8.0, 1.4 Hz, 1H), 7.52 (dt, J = 1.4, 7.4 Hz, 1H), 7.45 (d, J = 8.5 Hz, 2H), 7.42 (m, 5H), 7.36 (dt, J = 1.1, 8.2 Hz, 1H), 7.24 (d, J = 8.3 Hz, 1H), 7.11 (d, J = 8.5 Hz, 2H), 6.99 (q, J = 8.3 Hz, 1H), 5.42 (s, 2H). ^{13}C NMR (100 MHz, $CDCl_3$): δ 154.3, 153.9, 148.4 (q, J = 5.6 Hz), 135.1, 134.0, 132.7 (q, J = 1.2 Hz), 132.2, 132.1, 132.0, 131.6, 131.3, 128.7, 128.6, 127.9, 124.2, 123.0 (q, J = 34.2 Hz), 122.7 (q, J = 271.8 Hz), 121.8, 114.2, 45.5. ^{19}F NMR (376 MHz, $CDCl_3$) δ -56.11; HRESIMS calcd for $[C_{24}H_{16}BrF_3N_2O + H]^+$ 485.0476, found 485.0496.

Methyl (*E*)-4-((2-oxo-3-(3,3,3-trifluoro-1-phenylprop-1-en-1-yl)quinoxalin-1(2*H*)-yl) methyl)benzoate (**3m**)

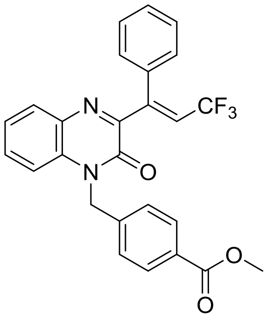

Yellow solid (65 mg, 70%). Mp: 161-163 ℃. ^{1}H NMR (400 MHz, $CDCl_3$): δ 8.00 (d, J = 8.3 Hz, 2H), 7.90 (dd, J = 1.1, 8.0 Hz, 1H), 7.49 (dt, J = 1.1, 8.4 Hz, 1H), 7.46-7.39 (m, 5H), 7.35 (t, J = 7.7 Hz, 1H), 7.27 (d, J = 7.4 Hz, 2H), 7.20 (d, J = 8.4 Hz, 1H), 7.03 (dq, J = 1.3, 8.2 Hz, 1H), 5.54 (d, J = 18.9 Hz, 2H), 3.90 (s, 3H). ^{13}C NMR (100 MHz, $CDCl_3$): δ 166.5, 154.2, 153.9, 148.4 (q, J = 5.6 Hz), 140.0, 135.1, 132.8, 132.7, 131.7, 131.3, 130.3, 129.8, 128.7 (q, J = 1.6 Hz), 128.6, 127.9, 126.8, 124.3, 123.0 (q, J = 34.3 Hz), 122.8 (q, J = 271.6 Hz), 114.2, 52.2, 45.8. ^{19}F NMR (376 MHz, $CDCl_3$) δ -56.16; HRESIMS calcd for $[C_{26}H_{19}F_3N_2O_3 + H]^+$ 465.1426, found 465.1445.

(*E*)-3-(3,3,3-trifluoro-1-phenylprop-1-en-1-yl)-1-(4-(trifluoromethyl)benzyl)quinoxalin-2(1*H*)-one (**3n**)

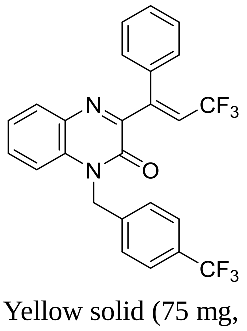

Yellow solid (75 mg, 79%). Mp: 101-102 ℃. ^{1}H NMR (400 MHz, $CDCl_3$): δ 7.91 (dd, J = 8.0, 1.4 Hz, 1H), 7.60 (d, J = 8.2 Hz, 2H), 7.53 (dt, J = 1.4, 8.6 Hz, 1H), 7.47-7.40 (m, 5H), 7.38 (dt, J = 1.0, 8.2 Hz, 1H), 7.34 (d, J = 8.2 Hz, 2H), 7.21 (d, J = 8.4 Hz, 1H), 7.00 (q, J = 8.2 Hz, 1H), 5.55 (d, J = 16.4 Hz, 2H). ^{13}C NMR (100 MHz, $CDCl_3$): δ 154.3, 153.9, 148.3 (q, J = 5.7 Hz), 139.0, 135.0, 132.8, 132.7, 131.7, 131.4, 130.3 (q, J = 32.7 Hz), 128.7 (q, J = 1.7 Hz), 128.6, 127.9, 127.2, 126.0 (q, J = 3.7 Hz), 124.3, 123.9 (q, J = 272.3 Hz), 123.0 (q, J = 34.2 Hz), 122.7 (q, J = 271.7 Hz), 114.1, 45.6. ^{19}F NMR (376 MHz, $CDCl_3$) δ -56.25, -62.67; HRESIMS calcd for $[C_{25}H_{16}F_6N_2O + H]^+$ 475.1245, found 475.1256.

(*E*)-3-(3,3,3-trifluoro-1-phenylprop-1-en-1-yl)-1-(4-(trifluoromethoxy)benzyl)quinoxalin-2(1*H*)-one (**3o**)

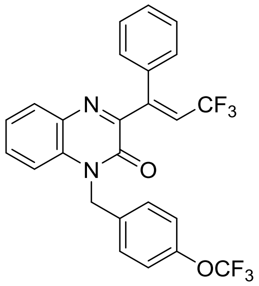

Yellow solid (64 mg, 65%). Mp: 124-126 ℃. ^{1}H NMR (400 MHz, $CDCl_3$): δ 7.92 (dd, *J* = 8.0, 1.3 Hz, 1H), 7.55 (dt, *J* = 1.4, 8.6 Hz, 1H), 7.48-7.40 (m, 5H), 7.38 (dt, *J* = 1.4, 8.1 Hz, 1H), 7.32-7.25 (m, 3H), 7.20 (d, *J* = 8.3 Hz, 2H), 7.03 (q, *J* = 8.3 Hz, 1H), 5.50 (d, *J* = 16.8 Hz, 2H). ^{13}C NMR (100 MHz, $CDCl_3$): δ 154.3, 153.9, 148.7 (q, *J* = 1.7 Hz), 148.5 (q, *J* = 5.5 Hz), 135.1, 133.6, 132.8, 132.7, 131.7, 131.4, 128.7 (q, *J* = 1.7 Hz), 128.6, 128.5, 127.9, 124.3, 123.0 (q, *J* = 34.3 Hz), 122.8 (q, *J* = 271.7 Hz), 121.5, 120.4 (q, *J* = 257.4 Hz), 114.2, 45.4. ^{19}F NMR (376 MHz, $CDCl_3$) δ -56.25, -62.67; HRESIMS calcd for $[C_{25}H_{16}F_6N_2O_2 + H]^+$ 491.1194, found 491.1216.

(*E*)-1-Ethyl-3-(3,3,3-trifluoro-1-phenylprop-1-en-1-yl)quinoxalin-2(1*H*)-one (**3p**)

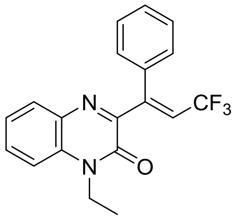

Yellow solid (57 mg, 83%). Mp: 130-132 ℃. ^{1}H NMR (400 MHz, $CDCl_3$): δ 7.90 (dd, *J* = 8.6, 1.5 Hz, 1H), 7.63 (dt, *J* = 1.5, 8.6 Hz, 1H), 7.45-7.40 (m, 5H), 7.36 (d, *J* = 7.9 Hz, 2H), 6.94 (dq, *J* = 1.0, 7.3 Hz, 1H), 4.31 (q, *J* = 7.2 Hz, 2H), 1.38 (t, *J* = 7.2 Hz, 3H). ^{13}C NMR (100 MHz, $CDCl_3$): δ 154.2, 153.4, 148.7 (q, *J* = 5.6 Hz), 135.2, 132.8, 132.5, 131.6, 131.3, 128.7 (q, *J* = 1.3 Hz), 128.5, 127.8, 123.8, 122.8 (q, *J* = 271.6 Hz), 122.5 (q, *J* = 34.2 Hz), 113.6, 37.6, 12.4. ^{19}F NMR (376 MHz, $CDCl_3$) δ -56.14; HRESIMS calcd for $[C_{19}H_{15}F_3N_2O + H]^+$ 345.1215, found 345.1226.

(*E*)-1-Hexyl-3-(3,3,3-trifluoro-1-phenylprop-1-en-1-yl)quinoxalin-2(1*H*)-one (**3q**)

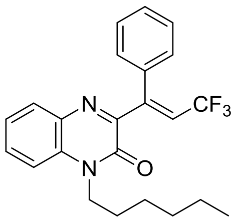

Yellow oil (62 mg, 78%). ^{1}H NMR (400 MHz, $CDCl_3$): δ 7.89 (dd, *J* = 8.1, 1.1 Hz, 1H), 7.62 (dt, *J* = 1.3, 8.5 Hz, 1H), 7.45-7.40 (m, 5H), 7.35 (t, *J* = 8.7 Hz, 2H), 6.89 (q, *J* = 8.3 Hz, 1H), 4.22 (t, *J* = 8.0 Hz, 2H), 1.80-1.72 (m, 2H), 1.90-1.42 (m, 2H), 1.38-1.30 (m, 4H), 0.92 (t, *J* = 6.8 Hz, 3H). ^{13}C NMR (100 MHz, $CDCl_3$): δ 154.3, 153.5, 148.7 (q, *J* = 5.6 Hz), 135.2, 132.8, 132.7, 131.4, 131.3, 128.6 (q, *J* = 1.8 Hz), 128.5, 127.8, 123.8, 122.8 (q, *J* = 271.7 Hz), 122.5 (q, *J* = 34.3 Hz),

113.7, 42.6, 31.5, 27.2, 26.7, 22.6, 14.0. ^{19}F NMR (376 MHz, $CDCl_3$) δ -56.18; HRESIMS calcd for $[C_{23}H_{23}F_3N_2O + H]^+$ 401.1841, found 401.1858.

(*E*)-1-(Cyclohexylmethyl)-3-(3,3,3-trifluoro-1-phenylprop-1-en-1-yl)quinoxalin-2(1*H*)-one (**3r**)

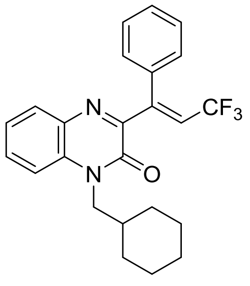

Yellow oil (55 mg, 67%). ^{1}H NMR (400 MHz, $CDCl_3$): δ 7.88 (dd, *J* = 8.0, 1.3 Hz, 1H), 7.60 (dt, *J* = 1.5, 8.6 Hz, 1H), 7.44-7.38 (m, 5H), 7.37 (dt, *J* = 1.1, 7.4 Hz, 1H), 7.36 (t, *J* = 8.5 Hz, 1H), 6.90 (dq, *J* = 1.0, 8.3 Hz, 1H), 4.13 (d, *J* = 7.2 Hz, 2H), 1.98-1.85 (m, 1H), 1.78-1.62 (m, 5H), 1.23-1.10 (m, 5H). ^{13}C NMR (100 MHz, $CDCl_3$): δ 154.4, 154.0, 148.7 (q, *J* = 5.5 Hz), 135.2, 133.2, 132.7, 131.3, 131.2, 128.6 (q, *J* = 1.5 Hz), 128.5, 127.8, 123.7, 122.8 (q, *J* = 271.7 Hz), 122.3 (q, *J* = 34.3 Hz), 114.2, 48.2, 36.5, 30.8, 26.1, 25.8. ^{19}F NMR (376 MHz, $CDCl_3$) δ -56.21; HRESIMS calcd for $[C_{24}H_{23}F_3N_2O + H]^+$ 413.1841, found 413.1853.

(*E*)-1-(But-3-en-1-yl)-3-(3,3,3-trifluoro-1-phenylprop-1-en-1-yl)quinoxalin-2(1*H*)-one (**3s**)

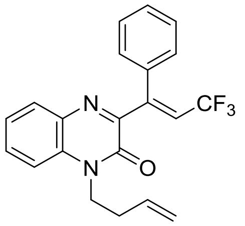

Yellow oil (57 mg, 77%). ^{1}H NMR (400 MHz, $CDCl_3$): δ 7.89 (dd, *J* = 8.0, 1.4 Hz, 1H), 7.63 (dt, *J* = 1.5, 8.6 Hz, 1H), 7.43-7.28 (m, 5H), 7.36 (dt, *J* = 1.4, 7.1 Hz, 2H), 6.90 (q, *J* = 8.3 Hz, 1H), 5.92-5.81 (m, 1H), 5.16-5.08 (m, 2H), 1.43 (dt, *J* = 1.7, 6.3 Hz, 2H), 2.52 (dd, *J* = 15.3, 7.0 Hz, 2H). ^{13}C NMR (100 MHz, $CDCl_3$): δ 154.3, 153.5, 148.6 (q, *J* = 5.5 Hz), 135.1, 133.6, 132.7, 132.6, 131.5, 131.3, 128.6 (q, *J* = 1.8 Hz), 128.5, 127.8, 123.9, 122.8 (q, *J* = 271.6 Hz), 122.5 (q, *J* = 34.4 Hz), 117.9, 113.6, 41.8, 31.4. ^{19}F NMR (376 MHz, $CDCl_3$) δ -56.23; HRESIMS calcd for $[C_{21}H_{17}F_3N_2O + H]^+$ 371.1371, found 371.1352.

(*E*)-1-(Prop-2-yn-1-yl)-3-(3,3,3-trifluoro-1-phenylprop-1-en-1-yl)quinoxalin-2(1*H*)-one (**3t**)

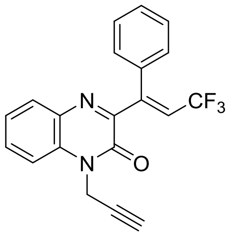

Yellow oil (46 mg, 65%). ^{1}H NMR (400 MHz, $CDCl_3$): δ 7.90 (dd, *J* = 8.1, 1.4 Hz, 1H), 7.67 (dt, *J* = 1.4, 8.6 Hz, 1H), 7.50 (dd, *J* = 8.4, 0.7 Hz, 1H), 7.43-7.38 (m, 6H), 6.97 (q, *J* = 8.3 Hz, 1H), 5.06 (dd, *J* = 22.7, 2.5 Hz, 2H), 2.33 (t, *J* = 2.5 Hz, 1H). ^{13}C NMR (100 MHz, $CDCl_3$): δ 154.0,

152.9, 148.3 (q, J = 5.7 Hz), 135.0, 132.7, 132.0, 131.7, 131.2, 128.7 (q, J = 1.7 Hz), 128.5, 127.9, 124.4, 123.0 (q, J = 34.2 Hz), 122.7 (q, J = 271.7 Hz), 114.0, 76.5, 73.6, 31.7. ^{19}F NMR (376 MHz, $CDCl_3$) δ -56.21; HRESIMS calcd for $[C_{20}H_{13}F_3N_2O + H]^+$ 355.1058, found 355.1067.

Ethyl (*E*)-2-(2-oxo-3-(3,3,3-trifluoro-1-phenylprop-1-en-1-yl)quinoxalin-1(2*H*)-yl)acetate (**3u**)

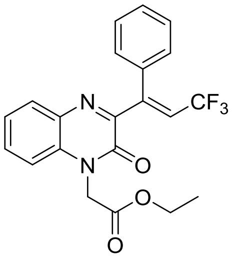

Yellow oil (60 mg, 75%). ^{1}H NMR (400 MHz, $CDCl_3$): δ 7.89 (dd, J = 8.0, 1.3 Hz, 1H), 7.59 (dt, J = 1.3, 7.5 Hz, 1H), 7.40 (s, 5H), 7.38 (dt, J = 1.0, 8.2 Hz, 1H), 7.11 (d, J = 8.2 Hz, 1H), 6.98 (q, J = 8.2 Hz, 1H), 5.00 (s, 2H), 4.26 (q, J = 7.1 Hz, 2H), 1.29 (t, J = 7.1 Hz, 3H). ^{13}C NMR (100 MHz, $CDCl_3$): δ 166.8, 153.9, 153.5, 148.2 (q, J = 5.6 Hz), 135.0, 132.7, 132.6, 131.8, 131.4, 128.7 (q, J = 1.7 Hz), 128.5, 127.8, 124.3, 123.0 (q, J = 34.2 Hz), 122.7 (q, J = 271.7 Hz), 113.2, 62.2, 43.6, 14.1. ^{19}F NMR (376 MHz, $CDCl_3$) δ -56.18; HRESIMS calcd for $[C_{21}H_{17}F_3N_2O_3 + H]^+$ 403.1270, found 403.1246.

(*E*)-1-(2-Oxo-2-phenylethyl)-3-(3,3,3-trifluoro-1-phenylprop-1-en-1-yl)quinoxalin-2(1*H*)-one (**3v**)

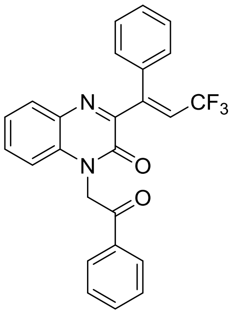

Yellow oil (44 mg, 51%). ^{1}H NMR (400 MHz, $CDCl_3$): δ 8.04 (d, J = 7.3 Hz, 2H), 7.91 (dd, J = 8.0, 1.3 Hz, 1H), 7.67 (t, J = 7.4 Hz, 1H), 7.53 (t, J = 7.7 Hz, 2H), 7.50 (t, J = 7.3 Hz, 1H), 7.46-7.38 (m, 5H), 7.36 (dt, J = 0.7, 7.7 Hz, 1H), 6.98 (q, J = 8.5 Hz, 2H), 5.72 (d, J = 12.9 Hz, 2H). ^{13}C NMR (100 MHz, $CDCl_3$): δ 190.8, 153.7, 153.6, 148.3 (q, J = 5.6 Hz), 135.1, 134.5, 134.3, 133.1, 132.7, 131.7, 131.3, 129.1, 128.7 (q, J = 1.8 Hz), 128.5, 128.1, 127.8, 124.1, 122.8 (q, J = 278.3 Hz), 122.9 (q, J = 34.2 Hz), 113.6, 48.6. ^{19}F NMR (376 MHz, $CDCl_3$) δ -56.19; HRESIMS calcd for $[C_{25}H_{17}F_3N_2O_2 + H]^+$ 435.1320, found 435.1326.

(*E*)-1-(Naphthalen-2-ylmethyl)-3-(3,3,3-trifluoro-1-phenylprop-1-en-1-yl)quinoxalin-2(1*H*)-one (**3w**)

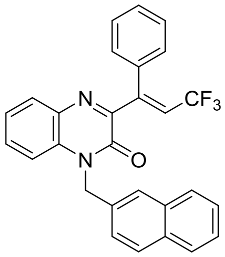

Yellow oil (47 mg, 52%). ^{1}H NMR (400 MHz, $CDCl_3$): δ 7.91 (dd, J = 8.0, 1.2 Hz, 1H), 7.84 (d, J = 8.4 Hz, 2H), 7.77 (t, J = 5.0 Hz, 1H), 7.64 (s, 1H), 7.60-7.45 (m, 8H), 7.37 (dt, J = 1.7, 9.9 Hz, 2H), 7.34 (dt, J = 1.0, 7.1 Hz, 1H), 7.09 (q, J = 8.3 Hz, 1H), 5.68 (d, J = 20.5 Hz, 2H). ^{13}C NMR (100 MHz, $CDCl_3$): δ 154.3, 154.1, 148.6 (q, J = 5.6 Hz), 135.2, 133.3, 133.0, 132.9, 132.8, 132.4, 131.6, 131.2, 129.0, 128.8 (q, J = 1.5 Hz), 128.6, 127.9, 127.8, 127.5, 126.5, 126.3, 125.7, 124.7, 124.1, 122.9 (q, J = 34.3 Hz), 122.8 (q, J = 271.6 Hz), 114.6, 46.3. ^{19}F NMR (376 MHz, $CDCl_3$) δ -56.10; HRESIMS calcd for $[C_{28}H_{19}F_3N_2O + H]^+$ 457.1528, found 457.1505.

(*E*)-3-(3,3,3-trifluoro-1-phenylprop-1-en-1-yl)quinoxalin-2(1*H*)-one (**3x**)

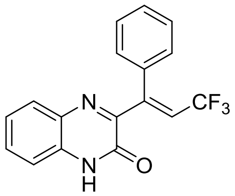

Yellow oil (51 mg, 81%). ^{1}H NMR (400 MHz, $CDCl_3$): δ 12.63 (s, 1H), 7.85 (dd, J = 8.2, 1.0 Hz, 1H), 7.57 (dt, J = 1.2, 8.3 Hz, 1H), 7.42 (s, 5H), 7.38 (dt, J = 1.2, 8.3 Hz, 1H), 7.14 (dd, J = 8.2, 1.0 Hz, 1H), 7.05 (q, J = 8.3 Hz, 1H). ^{13}C NMR (100 MHz, $CDCl_3$): δ 155.6, 155.4, 146.2 (q, J = 5.4 Hz), 136.3, 132.6, 131.4, 131.3, 129.9, 129.7, 128.9, 127.0, 124.6, 112.7 (q, J = 273.3 Hz), 118.7 (q, J = 34.3 Hz), 116.2. ^{19}F NMR (376 MHz, $CDCl_3$) δ -56.20; HRESIMS calcd for $[C_{17}H_{11}F_3N_2O + H]^+$ 317.0902, found 317.0911.

(*E*)-1-Methyl-3-(3,3,3-trifluoro-1-(*p*-tolyl)prop-1-en-1-yl)quinoxalin-2(1*H*)-one (**3y**)

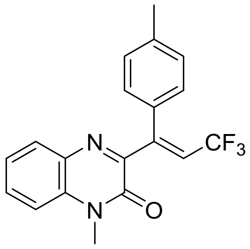

Yellow solid (59 mg, 86%). Mp: 107-108 ℃. ^{1}H NMR (400 MHz, $CDCl_3$): δ 8.16 (dd, J = 8.0, 1.3 Hz, 1H), 7.92 (dd, J = 8.5, 1.4 Hz, 1H), 7.63 (dt, J = 1.4, 8.5 Hz, 1H), 7.40 (dt, J = 1.3, 8.2 Hz, 1H), 7.34 (d, J = 8.0 Hz, 2H), 7.21 (d, J = 8.0 Hz, 2H), 6.70 (q, J = 8.2 Hz, 1H), 3.68 (s, 3H), 2.40 (s, 3H). ^{13}C NMR (100 MHz, $CDCl_3$): δ 154.9, 153.8, 148.9 (q, J = 5.8 Hz), 138.5, 133.7, 132.5, 132.1, 131.5, 131.0 128.7, 128.5 (q, J = 1.7 Hz), 124.0, 122.8 (q, J = 271.6 Hz), 121.7 (q, J = 34.3 Hz), 113.7, 29.2, 21.4. ^{19}F NMR (376 MHz, $CDCl_3$) δ -56.17; HRESIMS calcd for $[C_{19}H_{15}F_3N_2O + H]^+$ 345.1215, found 345.1213.

(*E*)-1-Methyl-3-(3,3,3-trifluoro-1-(4-methoxyphenyl)prop-1-en-1-yl)quinoxalin-2(1*H*)-one (**3z**)

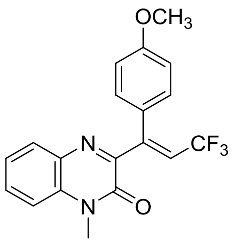

Yellow solid (53 mg, 74%). Mp: 166-168 ℃. ^{1}H NMR (400 MHz, $CDCl_3$): δ 7.92 (dd, J = 8.0, 1.4 Hz, 1H), 7.63 (dd, J = 8.5, 1.4 Hz, 1H), 7.40 (dt, J = 1.4, 8.0 Hz, 1H), 7.35 (d, J = 8.7 Hz, 2H), 7.34 (d, J = 8.7 Hz, 1H), 6.92 (dt, J = 1.4, 8.7 Hz, 2H), 6.63 (q, J = 8.3 Hz, 1H), 3.83 (s, 3H), 3.67 (s, 3H). ^{13}C NMR (100 MHz, $CDCl_3$): δ 154.8, 155.0, 153.8, 148.6 (q, J = 5.8 Hz), 133.7, 132.5, 131.4, 131.0, 129.9 (q, J = 1.7 Hz), 127.2, 124.0, 122.8 (q, J = 270.2 Hz), 121.3 (q, J = 34.3 Hz), 113.7, 113.4, 55.2, 29.2. ^{19}F NMR (376 MHz, $CDCl_3$) δ -56.19; HRESIMS calcd for $[C_{19}H_{15}F_3N_2O_2 + H]^+$ 361.1164, found 361.1176.

(E)-1-Methyl-3-(3,3,3-trifluoro-1-(2-fluorophenyl)prop-1-en-1-yl)quinoxalin-2(1H)-one (**3aa**)

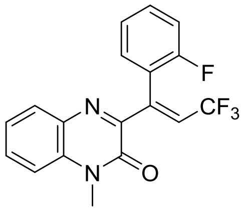

Yellow solid (49 mg, 70%). Mp: 137-138 ℃. ^{1}H NMR (400 MHz, $CDCl_3$): δ 7.76 (dd, J = 8.1, 1.3 Hz, 1H), 7.65 (t, J = 8.2 Hz, 1H), 7.61 (dt, J = 1.3, 9.1 Hz, 1H), 7.44-7.39 (s, 1H), 7.37-7.30 (s, 3H), 7.21 (dt, J = 1.0, 7.5 Hz, 1H), 7.12 (dt, J = 0.9, 9.5 Hz, 1H), 3.71 (s, 3H). ^{13}C NMR (100 MHz, $CDCl_3$): δ 159.7 (d, J = 247.0 Hz), 153.9, 151.4, 142.5 (q, J = 5.6 Hz), 133.3, 132.4, 131.8, 131.2, 130.9 (q, J = 1.4 Hz), 130.4 (d, J = 8.1 Hz), 126.0 (q, J = 34.1 Hz), 123.9, 123.5 (q, J = 3.4 Hz), 122.7 (q, J = 271.6 Hz), 115.1, 114.9, 113.7, 29.3. ^{19}F NMR (376 MHz, $CDCl_3$) δ -57.91, -113.43; HRESIMS calcd for $[C_{18}H_{12}F_4N_2O + H]^+$ 349.0964, found 349.0945.

(E)-3-(1-cyclohexyl-3,3,3-trifluoroprop-1-en-1-yl)-1-methylquinoxalin-2(1H)-one (**3ab**)

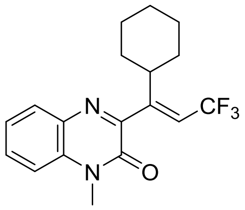

Yellow oil (38 mg, 57%). ^{1}H NMR (400 MHz, $CDCl_3$): δ 7.92 (d, J = 7.9, Hz, 1H), 7.63 (t, J = 8.4 Hz, 1H), 7.40 (t, J = 7.9 Hz, 1H), 7.36 (d, J = 8.4 Hz, 1H), 5.82 (q, J = 8.4 Hz, 1H), 3.74 (s, 3H), 3.02 (t, J = 1.5 Hz, 1H), 1.93 (d, J = 12.2 Hz, 2H), 1.76 (d, J = 12.2 Hz, 2H), 1.65 (d, J = 12.6 Hz, 1H), 1.47 (q, J = 12.6 Hz, 2H), 1.39-1.27 (m, 3H). ^{13}C NMR (100 MHz, $CDCl_3$): δ 156.2, 154.2, 154.0 (q, J = 5.5 Hz), 133.4, 132.1, 131.1, 130.6, 123.9, 123.2 (q, J = 272.2 Hz), 119.5 (q, J = 34.2 Hz), 113.8, 41.8, 31.1, 29.4, 26.3, 25.7. ^{19}F NMR (376 MHz, $CDCl_3$) δ -56.88; HRESIMS calcd for $[C_{18}H_{19}F_3N_2O + H]^+$ 337.1528, found 337.1540.

(E)-3-(1-cyclopropyl-3,3,3-trifluoroprop-1-en-1-yl)-1-methylquinoxalin-2(1H)-one (**3ac**)

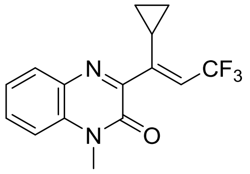

Yellow oil (48 mg, 81%). ^{1}H NMR (400 MHz, $CDCl_3$): δ 7.80 (dd, J = 8.0, 1.1 Hz, 1H), 7.52 (dt, J = 1.3, 8.4 Hz, 1H), 7.32 (dt, J = 1.1, 8.0 Hz, 1H), 7.28 (d, J = 8.4 Hz, 1H), 5.89-5.80 (m, 1H), 5.45-5.36 (m, 1H), 3.67 (s, 3H), 3.08 (dt, J = 1.2, 7.1 Hz, 2H), 2.69-2.62 (m, 2H). ^{13}C NMR (100 MHz, $CDCl_3$): δ 205.2 (q, J = 5.8 Hz), 158.9, 154.7, 132.8 (d, J = 46.8 Hz), 129.8, 129.7, 123.6, 122.7 (q, J = 270.3 Hz), 113.6, 97.8, 86.3 (q, J = 38.9 Hz), 32.6, 29.0, 24.3. ^{19}F NMR (376 MHz, $CDCl_3$) δ -60.60; HRESIMS calcd for $[C_{15}H_{13}F_3N_2O + H]^+$ 295.1058, found 295.1067.

(E)-1-Methyl-3-(1,1,1-trifluorohept-2-en-3-yl)quinoxalin-2(1H)-one (**3ad**)

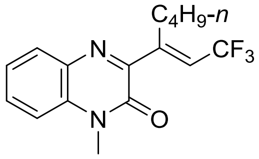

Yellow oil (55 mg, 89%). ^{1}H NMR (400 MHz, $CDCl_3$): δ 7.88 (d, J = 8.0, Hz, 1H), 7.61 (t, J = 8.4 Hz, 1H), 7.37 (t, J = 7.7 Hz, 1H), 7.33 (d, J = 8.4 Hz, 1H), 7.02 (q, J = 8.9 Hz, 1H), 3.72 (s, 3H), 2.92 (t, J = 7.2 Hz, 2H), 1.47-1.33 (m, 4H), 0.89 (t, J = 7.2 Hz, 3H). ^{13}C NMR (100 MHz, $CDCl_3$): δ 154.0, 152.7, 149.6 (q, J = 5.6 Hz), 133.3, 132.3, 131.4, 130.8, 123.9, 123.7 (q, J = 271.7 Hz), 122.7 (q, J = 33.8 Hz), 113.7, 31.0, 29.3, 29.2, 22.8, 13.8. ^{19}F NMR (376 MHz, $CDCl_3$) δ -57.02; HRESIMS calcd for $[C_{16}H_{17}F_3N_2O + H]^+$ 311.1371, found 311.1372.

(E)-1-Methyl-3-(1,1,1-trifluorotridec-2-en-3-yl)quinoxalin-2(1H)-one (**3ae**)

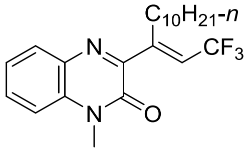

Yellow oil (56 mg, 71%). ^{1}H NMR (400 MHz, $CDCl_3$): δ 7.91 (dd, J = 8.0, 1.3 Hz, 1H), 7.63 (dt, J = 1.5, 8.6 Hz, 1H), 7.40 (dt, J = 1.3, 8.0 Hz, 1H), 7.35 (d, J = 8.6 Hz, 1H), 7.00 (q, J = 8.9 Hz, 1H), 3.74 (s, 3H), 2.93 (t, J = 7.0 Hz, 2H), 1.47-1.41 (m, 2H), 1.40-1.34 (m, 2H), 1.30-1.20 (m, 12H), 0.88 (t, J = 6.9 Hz, 3H). ^{13}C NMR (100 MHz, $CDCl_3$): δ 154.0, 152.9, 149.6 (q, J = 5.6 Hz), 133.3, 132.4, 131.4, 130.8, 123.9, 122.6 (q, J = 33.9 Hz), 122.3 (q, J = 271.6 Hz), 113.7, 36.1, 31.9, 29.7, 29.6, 29.5, 29.4, 29.3, 28.9, 26.9, 22.7, 14.1. ^{19}F NMR (376 MHz, $CDCl_3$) δ -57.04; HRESIMS calcd for $[C_{22}H_{29}F_3N_2O + H]^+$ 395.2310, found 395.2325.

(E)-7,7,7-Trifluoro-5-(4-methyl-3-oxo-3,4-dihydroquinoxalin-2-yl)hept-5-enenitrile (**3af**)

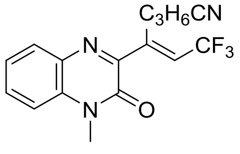

Yellow oil (36 mg, 56%). ^{1}H NMR (400 MHz, $CDCl_3$): δ 7.89 (dd, J = 8.0, 1.3 Hz, 1H), 7.65 (dt, J

= 1.4, 8.6 Hz, 1H), 7.41 (dt, *J* = 1.4, 8.0 Hz, 1H), 7.38 (d, *J* = 8.6 Hz, 1H), 5.94 (q, *J* = 8.0 Hz, 1H), 3.74 (s, 3H), 2.76 (dt, *J* = 1.3, 8.6 Hz, 2H), 2.54 (t, *J* = 7.2 Hz, 2H), 1.89 (dt, *J* = 7.2, 14.5 Hz, 2H). ^{13}C NMR (100 MHz, $CDCl_3$): *δ* 155.1, 153.6, 146.0 (q, *J* = 5.3 Hz), 133.4, 132.4, 131.4, 130.6, 124.1, 122.1 (q, *J* = 271.1 Hz), 119.5 (q, *J* = 33.1 Hz), 119.3, 113.9, 34.9, 29.3, 22.8, 16.2. ^{19}F NMR (376 MHz, $CDCl_3$) *δ* -59.46; HRESIMS calcd for $[C_{16}H_{14}F_3N_3O + H]^+$ 322.1167, found 322.1172.

(*E*)-3-(6-Chloro-1,1,1-trifluorohex-2-en-3-yl)-1-methylquinoxalin-2(1*H*)-one (**3ag**)

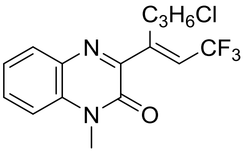

Yellow oil (42 mg, 63%). ^{1}H NMR (400 MHz, $CDCl_3$): *δ* 7.90 (dd, *J* = 8.0, 1.4 Hz, 1H), 7.63 (dt, *J* = 1.3, 8.8 Hz, 1H), 7.40 (dt, *J* = 1.4, 8.0 Hz, 1H), 7.37 (d, *J* = 8.3 Hz, 1H), 5.92 (tq, *J* = 1.3, 8.3 Hz, 1H), 3.74 (s, 3H), 3.66 (t, *J* = 6.4 Hz, 2H), 2.76 (dt, *J* = 1.6, 8.8 Hz, 2H), 2.01 (dt, *J* = 6.4, 12.9 Hz, 2H). ^{13}C NMR (100 MHz, $CDCl_3$): *δ* 155.6, 153.5, 146.9 (q, *J* = 5.3 Hz), 133.4, 132.5, 131.2, 130.6, 124.0, 122.3 (q, *J* = 271.0 Hz), 118.7 (q, *J* = 34.0 Hz), 113.9, 43.8, 33.3, 29.6, 29.2. ^{19}F NMR (376 MHz, $CDCl_3$) *δ* -59.24; HRESIMS calcd for $[C_{15}H_{14}ClF_3N_2O + H]^+$ 331.0825, found 331.0820.

(*E*)-1-Methyl-3-(1,1,1-trifluoro-7-hydroxyhept-2-en-3-yl)quinoxalin-2(1*H*)-one (**3ah**)

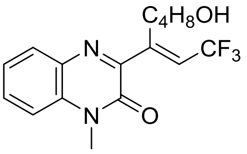

Yellow oil (21 mg, 32%). ^{1}H NMR (400 MHz, $CDCl_3$): *δ* 7.89 (dd, *J* = 8.0, 1.3 Hz, 1H), 7.62 (dt, *J* = 1.4, 8.6 Hz, 1H), 7.39 (dt, *J* = 1.4, 8.0 Hz, 1H), 7.36 (d, *J* = 8.6 Hz, 1H), 5.87 (q, *J* = 8.0 Hz, 1H), 3.73 (s, 3H), 3.66 (t, *J* = 5.8 Hz, 2H), 2.60 (d, *J* = 7.4 Hz, 2H), 2.10 (s, 1H), 1.69-1.60 (m, 4H). ^{13}C NMR (100 MHz, $CDCl_3$): *δ* 156.1, 153.6, 148.5 (q, *J* = 5.3 Hz), 133.3, 132.5, 131.1, 130.5, 124.0, 122.6 (q, *J* = 270.9 Hz), 117.7 (q, *J* = 33.6 Hz), 113.9, 62.2, 35.9, 31.9, 29.3, 23.0. ^{19}F NMR (376 MHz, $CDCl_3$) *δ* -59.06; HRESIMS calcd for $[C_{16}H_{17}F_3N_2O_2 + H]^+$ 327.1320, found 327.1328.

(*E*)-7,7,7-Trifluoro-5-(4-methyl-3-oxo-3,4-dihdroquinoxalin-2-yl)hept-5-en-1-yl acetate (**3ai**)

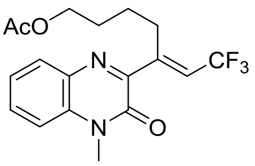

Yellow oil (50 mg, 68%). ^{1}H NMR (400 MHz, $CDCl_3$): *δ* 7.89 (dd, *J* = 8.0, 1.3 Hz, 1H), 7.62 (dt, *J* = 1.3, 8.5 Hz, 1H), 7.39 (dt, *J* = 1.0, 8.0 Hz, 1H), 7.36 (t, *J* = 8.5 Hz, 1H), 7.12 (q, *J* = 8.0 Hz, 1H), 4.06 (t, *J* = 6.6 Hz, 2H), 3.73 (s, 3H), 2.95 (t, *J* = 7.1 Hz, 2H), 1.98 (s, 3H), 1.74-1.67 (m, 2H), 1.57-1.50 (m, 2H). ^{13}C NMR (100 MHz, $CDCl_3$): *δ* 171.2, 154.1, 152.2, 148.6 (q, *J* = 5.6 Hz), 133.3, 132.3, 131.5, 130.8, 124.0, 123.6 (q, *J* = 271.6 Hz), 123.3 (q, *J* = 33.8 Hz), 113.7, 64.1, 29.3, 28.9, 28.4, 25.3, 20.9. ^{19}F NMR (376 MHz, $CDCl_3$) *δ* -57.04; HRESIMS calcd for $[C_{18}H_{19}F_3N_2O_3 + H]^+$ 369.1426, found 369.1424.

(*E*)-1-Methyl-3-(3,3,3-trifluoro-2-methyl-1-phenylprop-1-en-1-yl)quinoxalin-2(1*H*)-one (**3aj**)

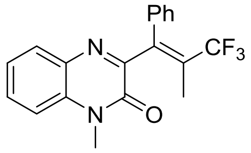

Yellow solid (37 mg, 53%). Mp: 152-153 ℃. ^{1}H NMR (400 MHz, CDCl$_3$): δ 7.96 (dd, *J* = 8.0, 1.4 Hz, 1H), 7.61 (dt, *J* = 1.5, 7.4 Hz, 1H), 7.51 (dd, *J* = 8.1, 1.5 Hz, 2H), 7.40 (dt, *J* = 1.1, 8.1 Hz, 1H), 7.36-7.30 (m, 4H), 3.68 (s, 3H), 1.94 (s, 3H). ^{13}C NMR (100 MHz, CDCl$_3$): δ 156.9, 153.3, 142.3 (q, *J* = 3.7 Hz), 136.3, 133.4, 132.7, 131.0, 130.6, 128.6 (q, *J* = 1.9 Hz), 128.0, 127.9, 127.3 (q, *J* = 29.2 Hz), 123.9, 123.8 (q, *J* = 275.9 Hz),113.8, 29.2, 15.3 (q, *J* = 2.3 Hz). ^{19}F NMR (376 MHz, CDCl$_3$) δ -60.20; HRESIMS calcd for [$C_{19}H_{15}F_3N_2O$ + H]$^+$ 345.1215, found 345.1235.

(*Z*/*E*)-1-Methyl-3-(3,3,3-trifluoro-1,2-diphenylprop-1-en-1-yl)quinoxalin-2(1*H*)-one (**3ak**)

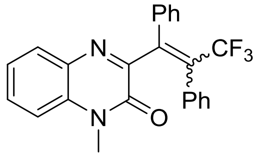

Inseparable yellow oil (42 mg, combined yield: 51%). ^{1}H NMR (400 MHz, CDCl$_3$): δ Z + E 8.03 (dd, *J* = 8.0, 1.4 Hz, 1H), 7.81 (dd, *J* = 8.0, 1.4 Hz, 1H), 7.72 (dd, *J* = 8.5, 1.5 Hz, 2H), 7.58 (dt, *J* = 1.4, 8.5 Hz, 1H), 7.50 (d, *J* = 7.3 Hz, 2H), 7.44 (dt, *J* = 1.4, 8.5 Hz, 2H), 7.43-7.38 (m, 4H), 7.37-7.32 (m, 4H), 7.30 (d, *J* = 3.6 Hz, 1H), 7.26-7.23 (m, 3H), 7.22-7.19 (m, 3H), 7.17 (dt, *J* = 1.4, 7.2 Hz, 1H), 7.15-7.12 (m, 2H), 7.09 (q, *J* = 8.2 Hz, 1H), 3.69 (s, 3H), 3.27 (s, 3H). ^{13}C NMR (100 MHz, CDCl$_3$): δ Z + E 156.8, 156.7, 153.8, 153.3, 145.6 (q, *J* = 3.3 Hz), 144.1 (q, *J* = 3.5 Hz), 136.2, 135.8, 133.6, 133.5, 133.4, 133.2, 133.1, 133.0, 132.9, 132.8, 132.4 (q, *J* = 30.0 Hz), 131.6 (q, *J* = 29.5 Hz), 131.0, 130.8, 130.7, 130.6, 130.3, 130.1, 129.4, 128.6 (q, *J* = 1.9 Hz), 128.4 (q, *J* = 2.2 Hz), 128.3, 128.1, 128.0, 127.9, 127.8, 123.9, 123.7, 123.5 (q, *J* = 275.2 Hz), 123.2 (q, *J* = 276.0 Hz), 113.8, 113.6, 29.2, 28.9. ^{19}F NMR (376 MHz, CDCl$_3$) δ Z + E -56.98, -59.16; HRESIMS calcd for [$C_{24}H_{17}F_3N_2O$ + H]$^+$ 407.1371, found 407.1369.

7. References

1. Jr. W. S. Hummers, R. E. Offeman. Preparation of graphitic oxide. *J. Am. Chem. Soc.*, **1958**, *80*, 1339−1339.
2. D. A. Dikin, S. Stankovich, E. J. Zimney, R. D. Piner, G. H. B. Dommett, G. Evmenenko, S. T. Nguyen, R. S. Ruoff. Preparation and characterization of graphene oxide paper. *Nature*, **2007**, *448*, 457−460.
3. (a) T. Soejima, Y. Maru, S. Ito. Facile low-temperature synthesis and photocatalytic activity of graphene oxide/TiO_2 composite. *Bull. Chem. Soc. Jpn.*, **2013**, *86*, 1065−1070. (b) C.-M. Chen, J.-Q. Huang, Q. Zhang, W.-Z. Gong, Q.-H. Yang, M.-Z. Wang, Y.-G. Yang. Annealing a graphene oxide film to produce a free standing high conductive graphene film. *Carbon*, **2012**, *50*, 659−667. (c) C. W. Lo, D. Zhu, H. Jiang. An infrared-light responsive graphene-oxide incorporated poly (N-isopropylacrylamide) hydrogel nanocomposite. *Soft Matter*, **2011**, *7*, 5604−5609. (d) S, Lv. Y. Ma, C. Qiu, T. Sun, J. Liu, Q. Zhou. Effect of graphene oxide nanosheets of microstructure and mechanical properties of cement composites. *Constr. Build. Mater.*, **2013**, *49*, 121−127.
4. V. B. Rana, S. K. Sahni, S. K. Sangal. Oxovanadium (IV) complexes of potential pentadentate ligands. *J. Inorg. Nucl. Chem.*, **1979**, *41*, 1498−1500.

8. Copies of ^{1}H NMR, ^{19}F NMR and ^{13}C NMR Spectra for All Compounds

^{1}H and ^{13}C NMR Spectra for **3a**

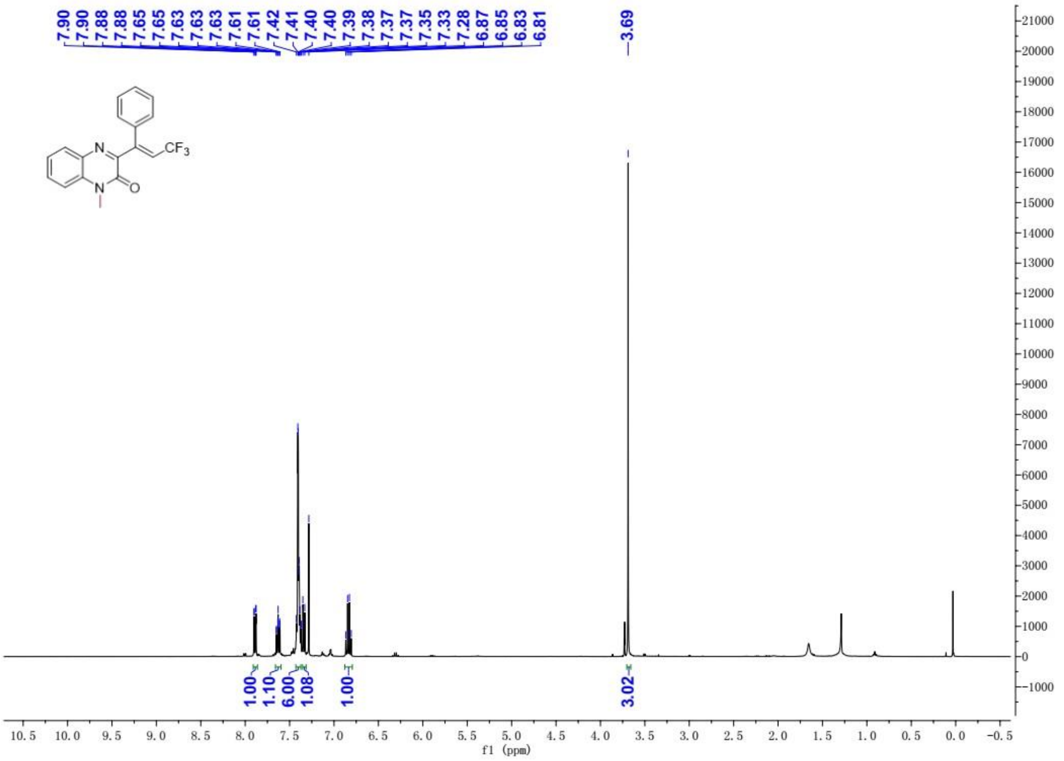

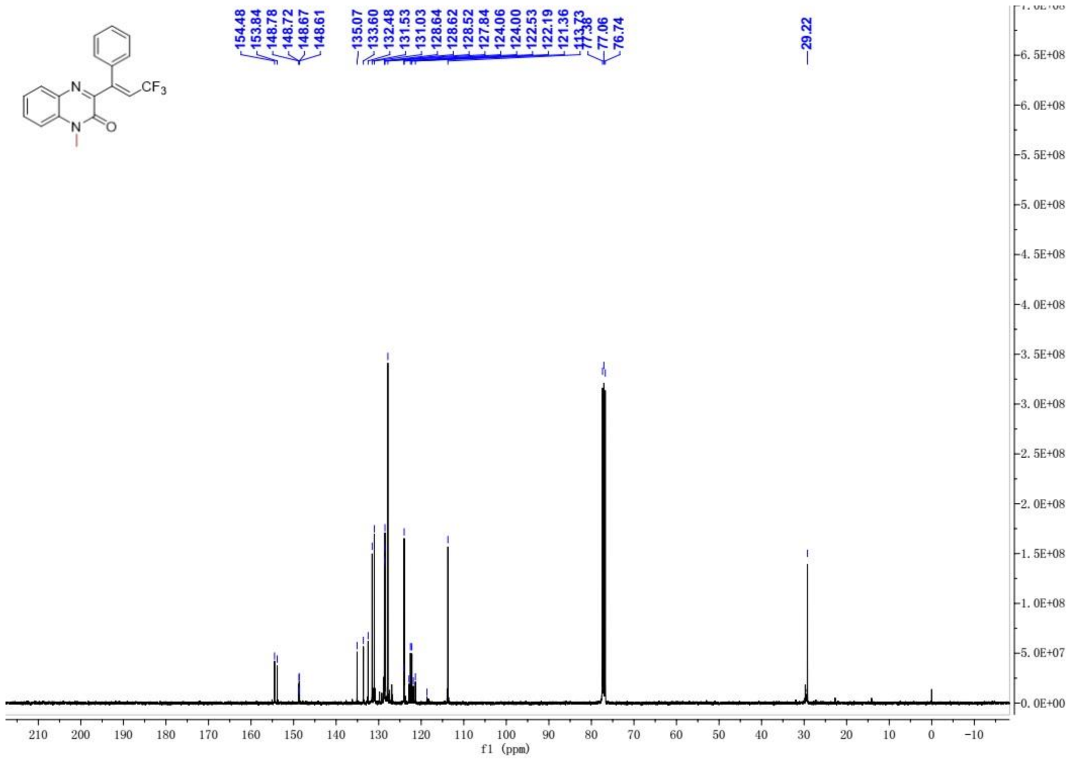

^{1}H and ^{13}C NMR Spectra for **3b**

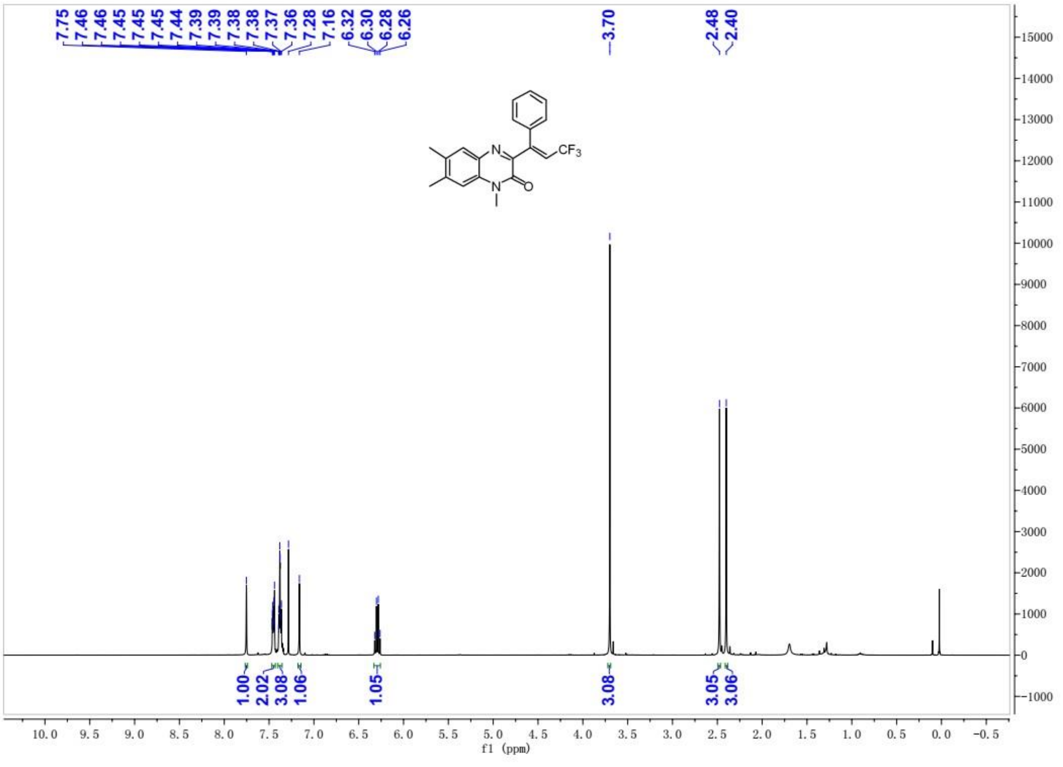

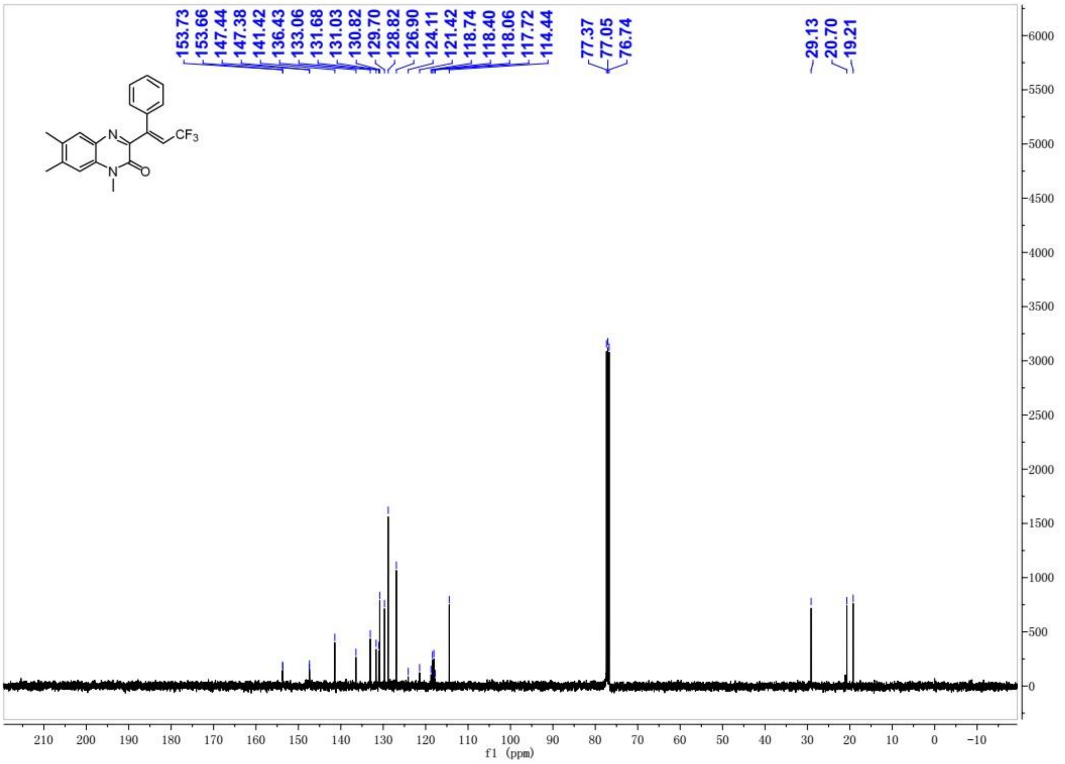

^{1}H and ^{13}C NMR Spectra for **3c**

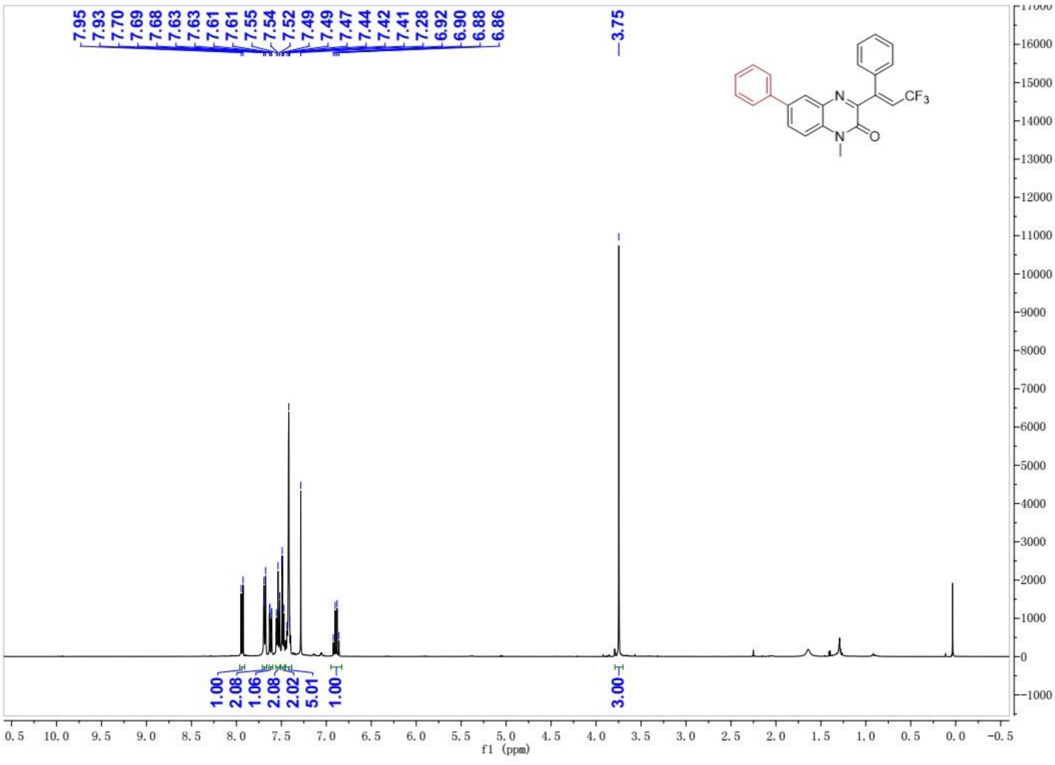

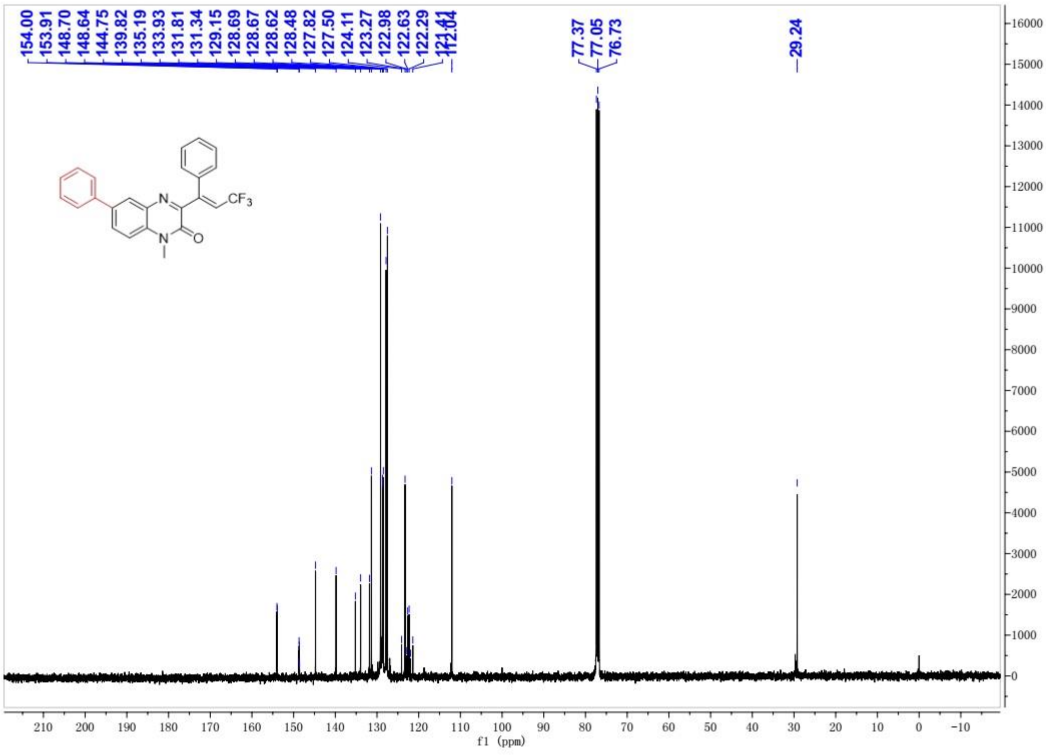

^{1}H and ^{13}C NMR Spectra for **3d**

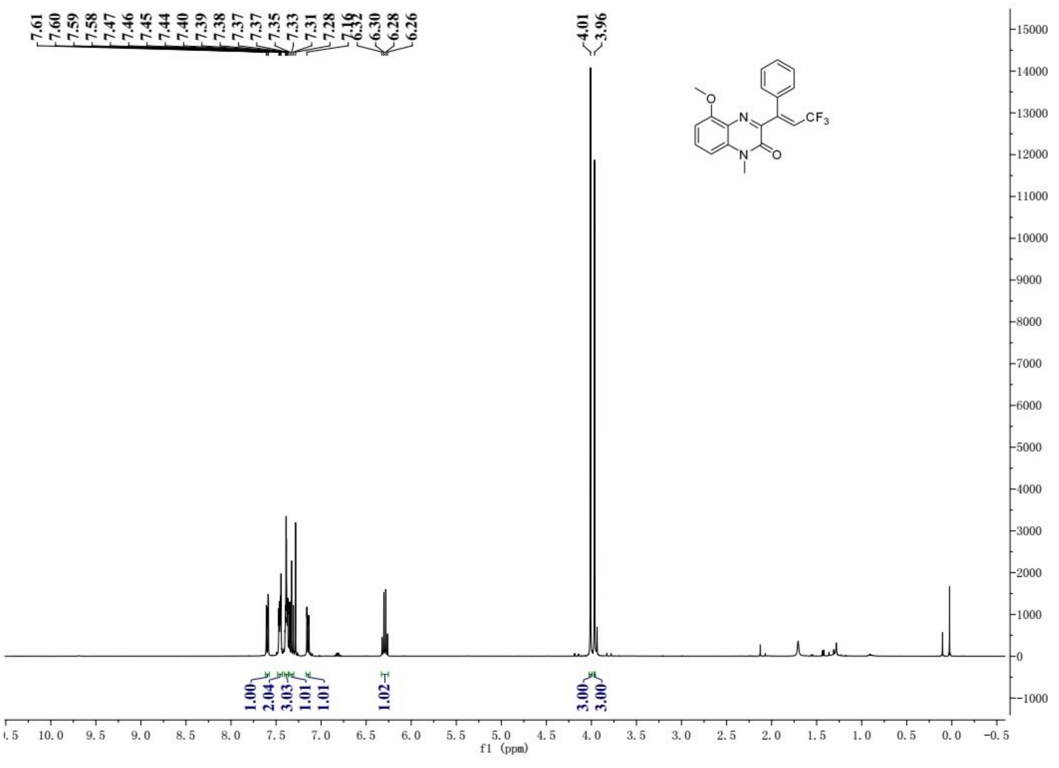

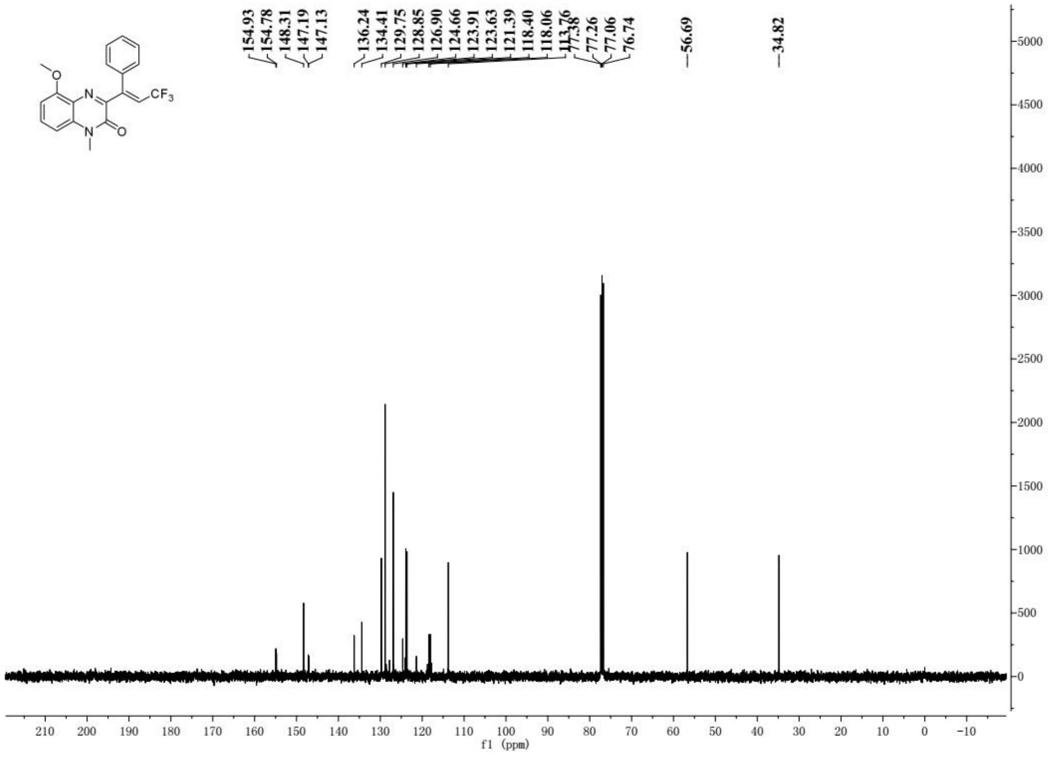

^{1}H and ^{13}C NMR Spectra for **3e**

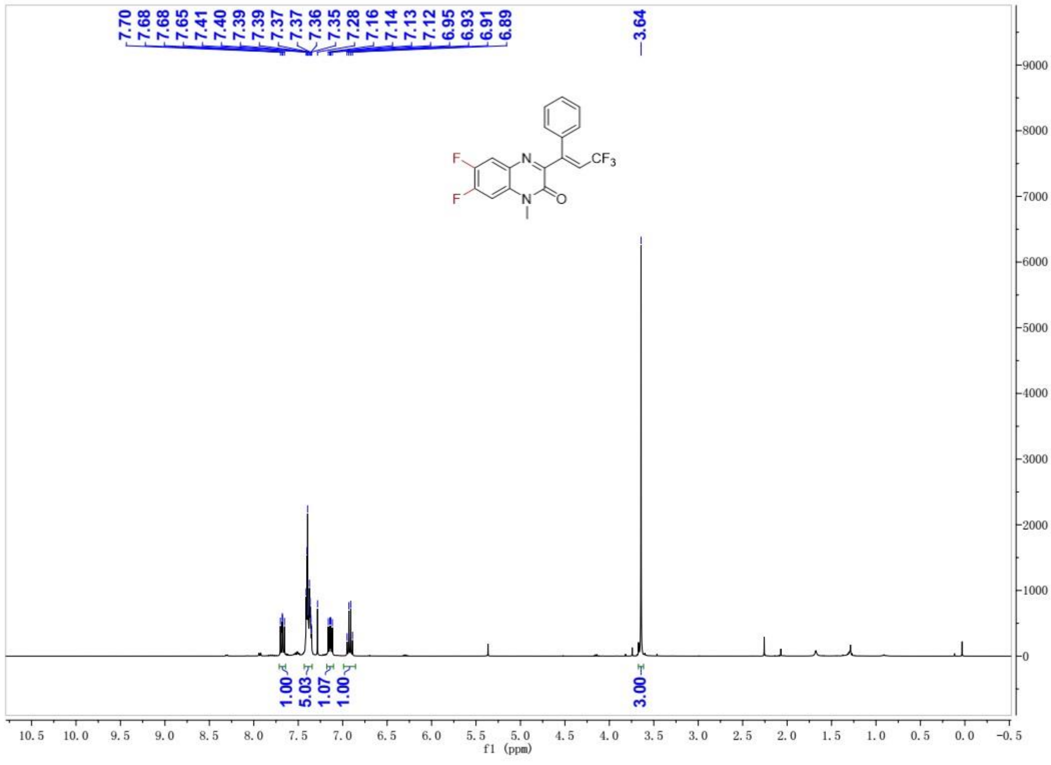

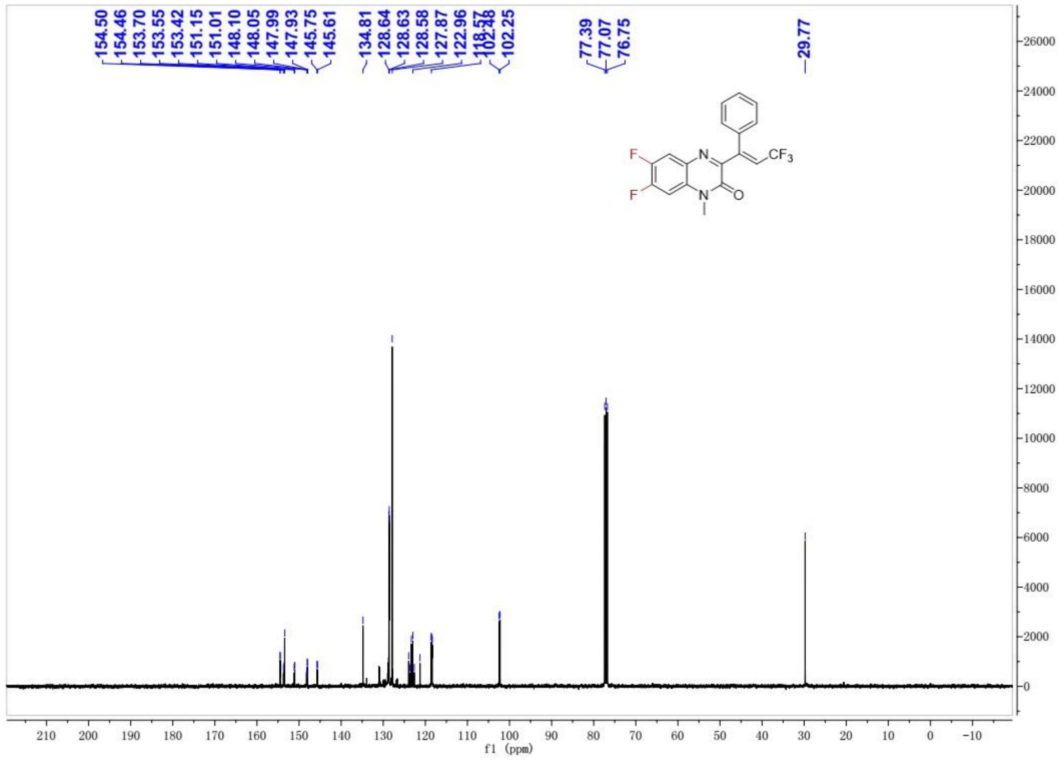

^{1}H and ^{13}C NMR Spectra for **3f**

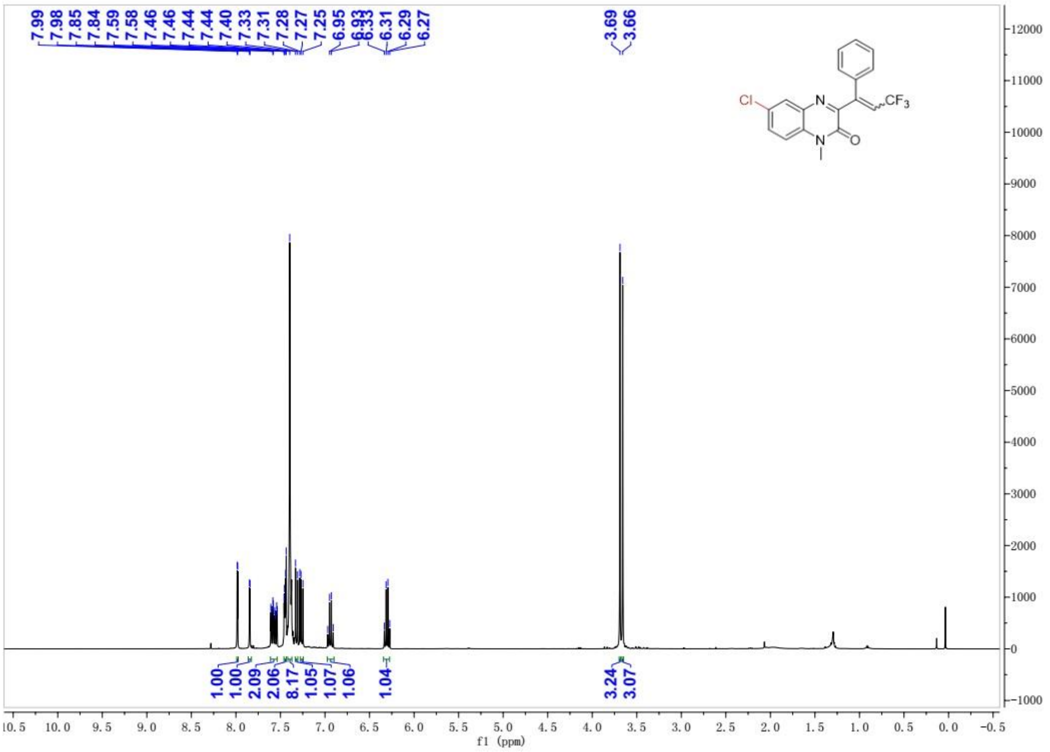

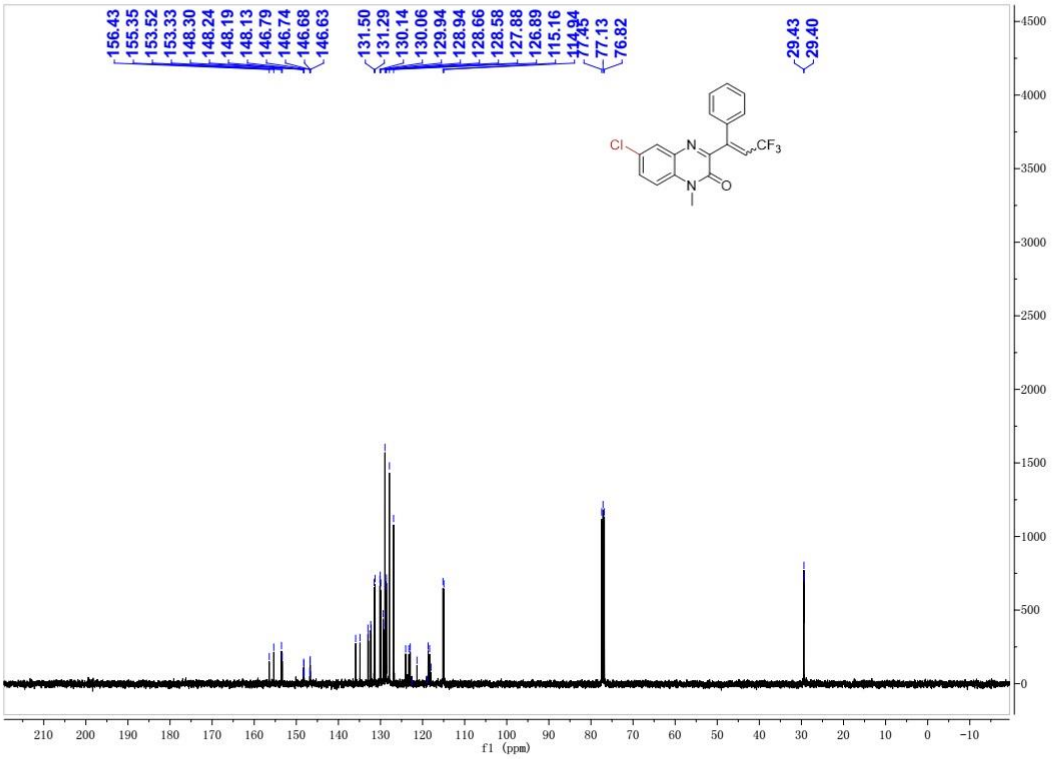

^{1}H and ^{13}C NMR Spectra for **3g**

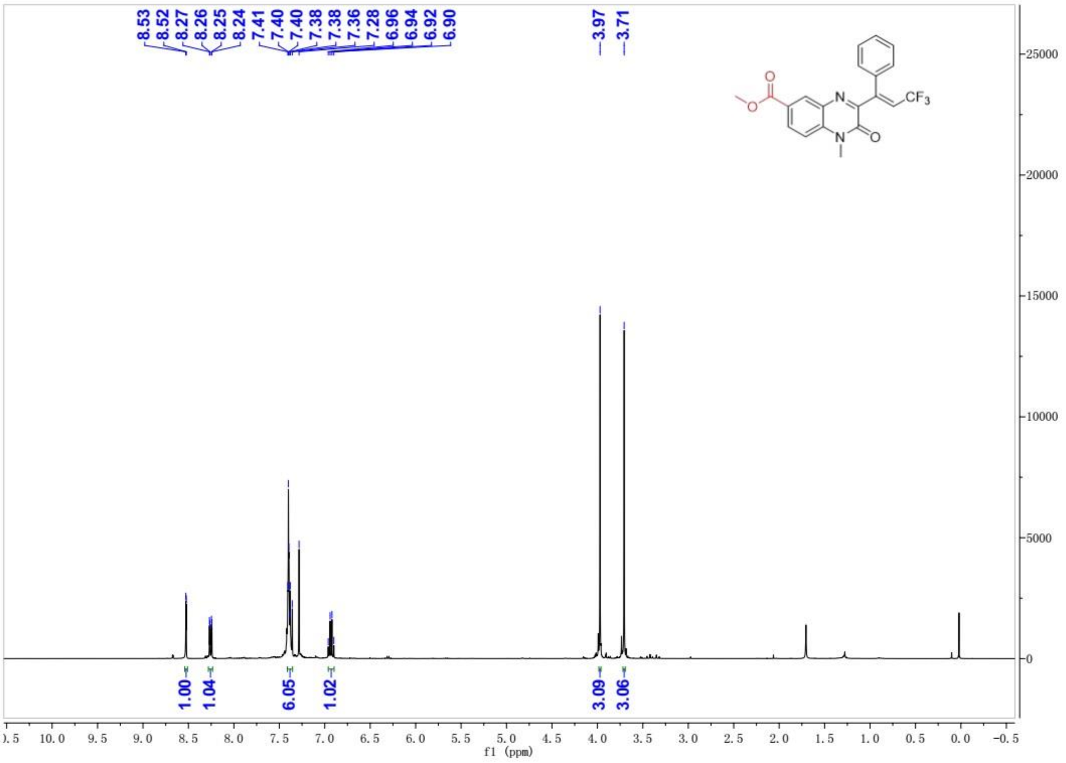

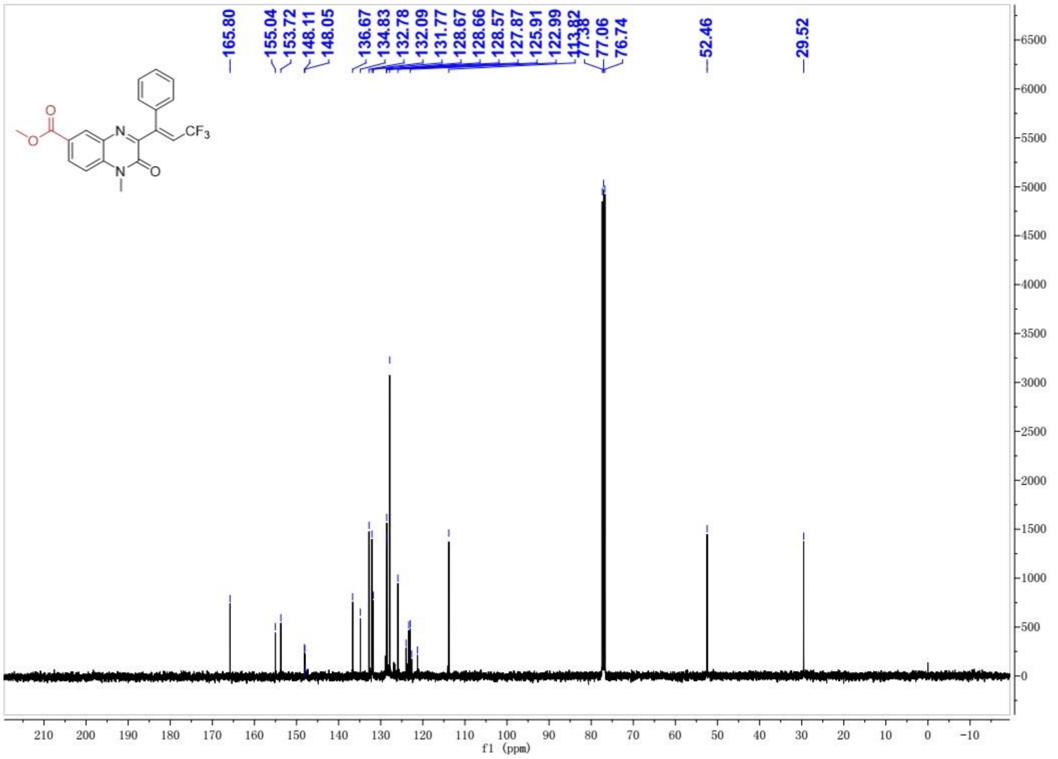

^{1}H and ^{13}C NMR Spectra for **3h**

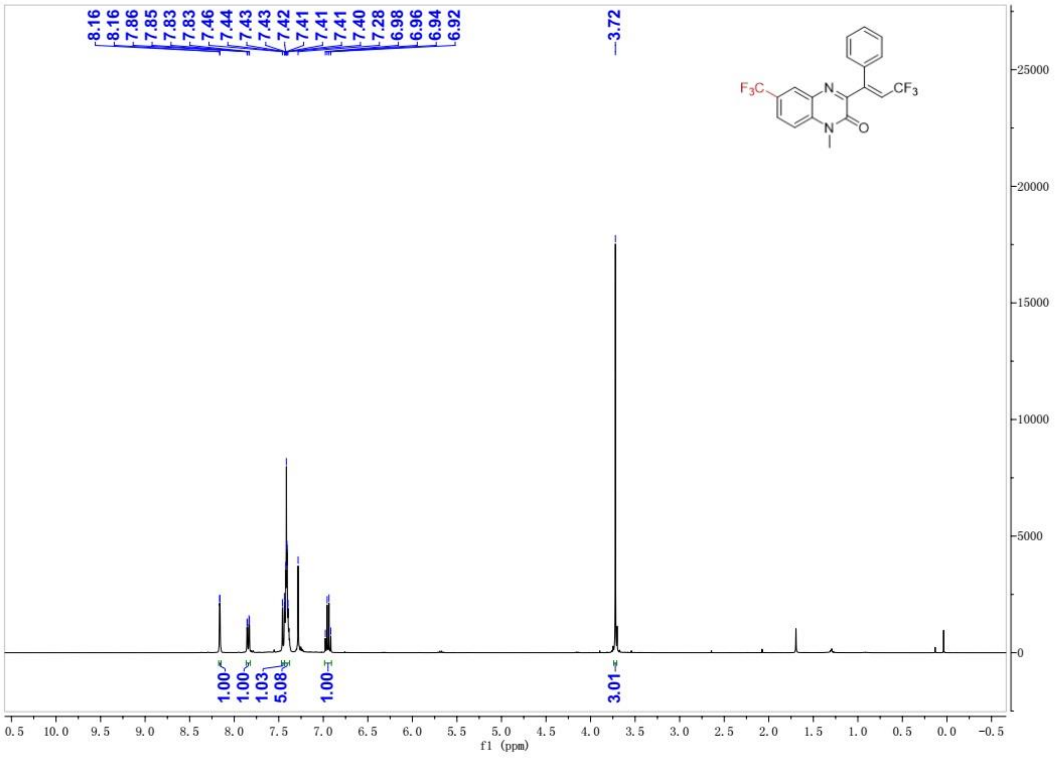

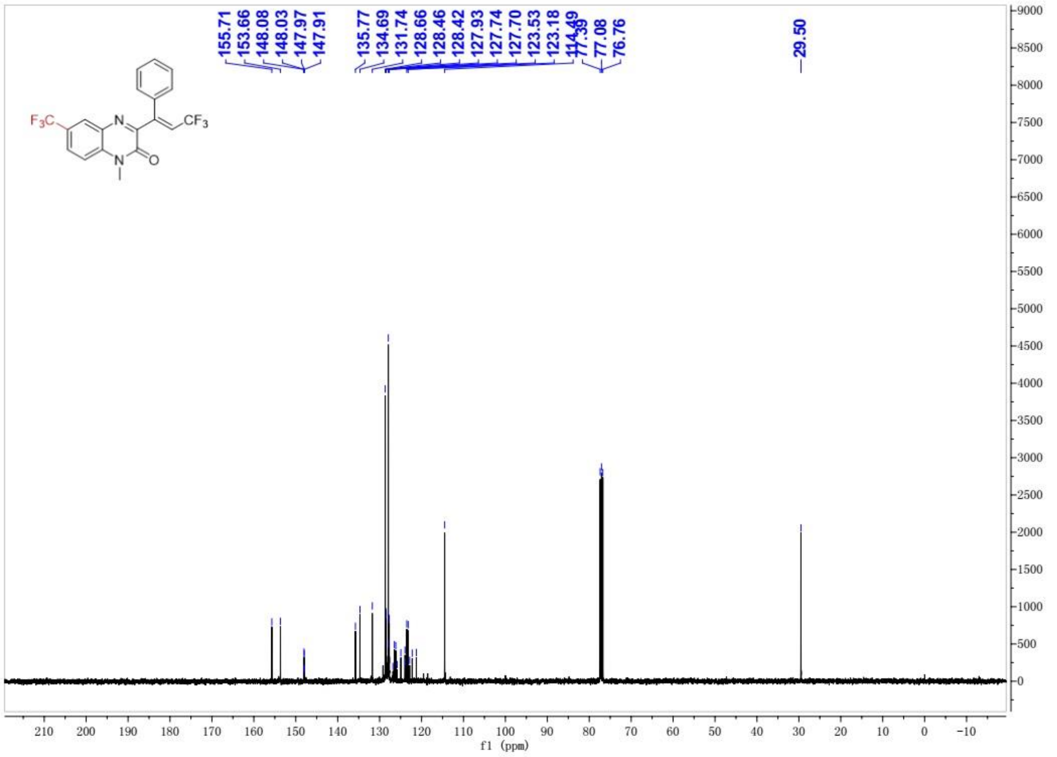

^{1}H and ^{13}C NMR Spectra for **3i**

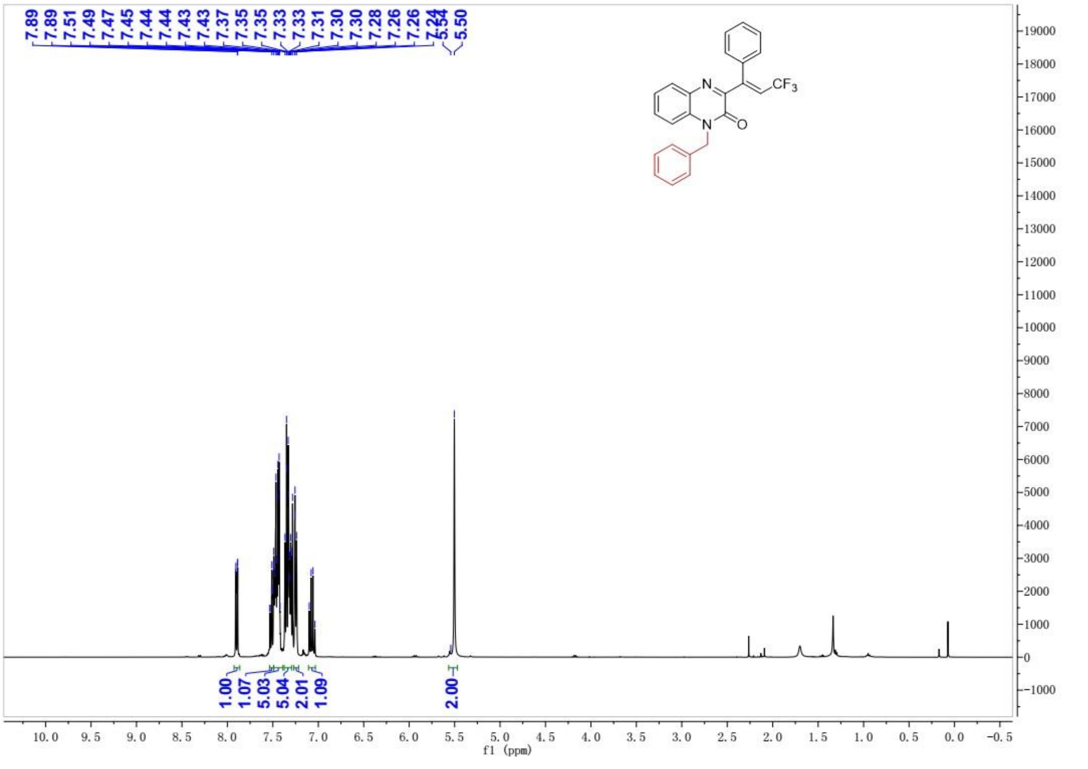

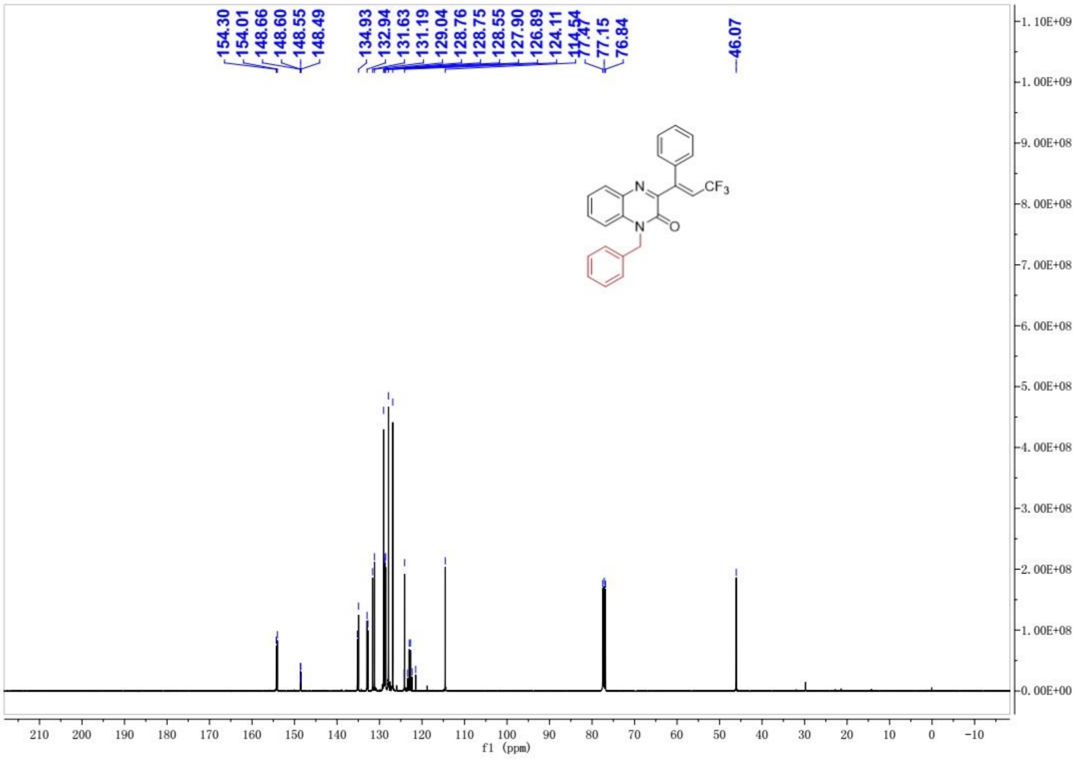

^{1}H and ^{13}C NMR Spectra for **3j**

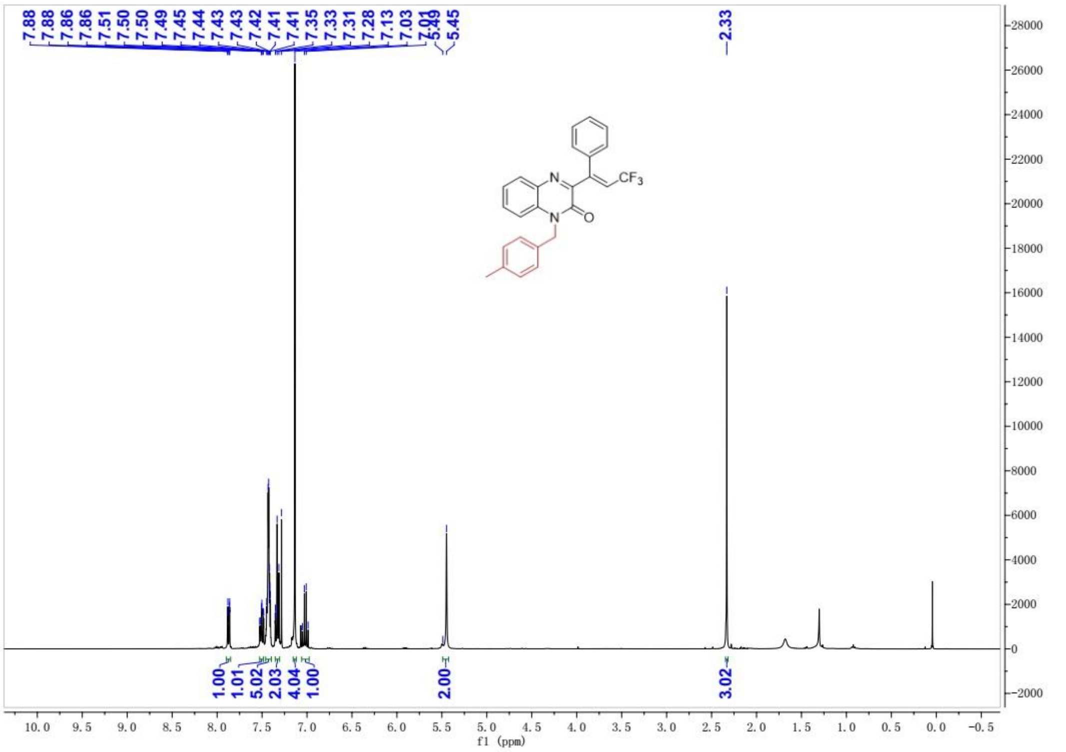

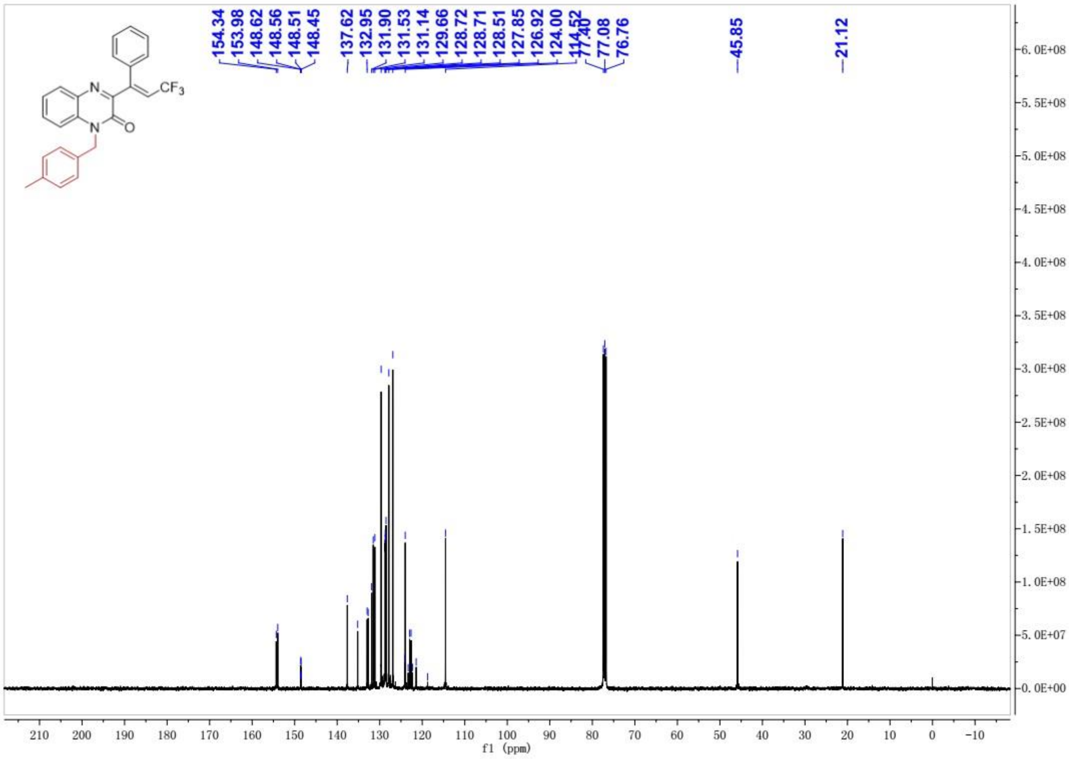

^{1}H and ^{13}C NMR Spectra for **3k**

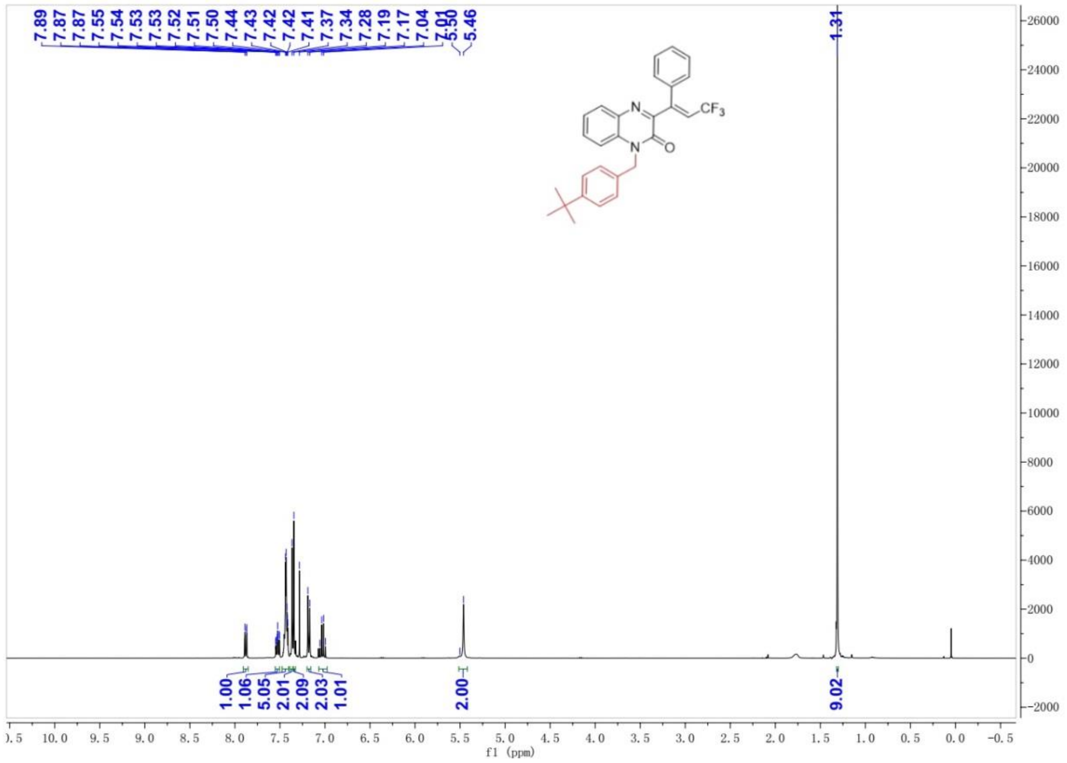

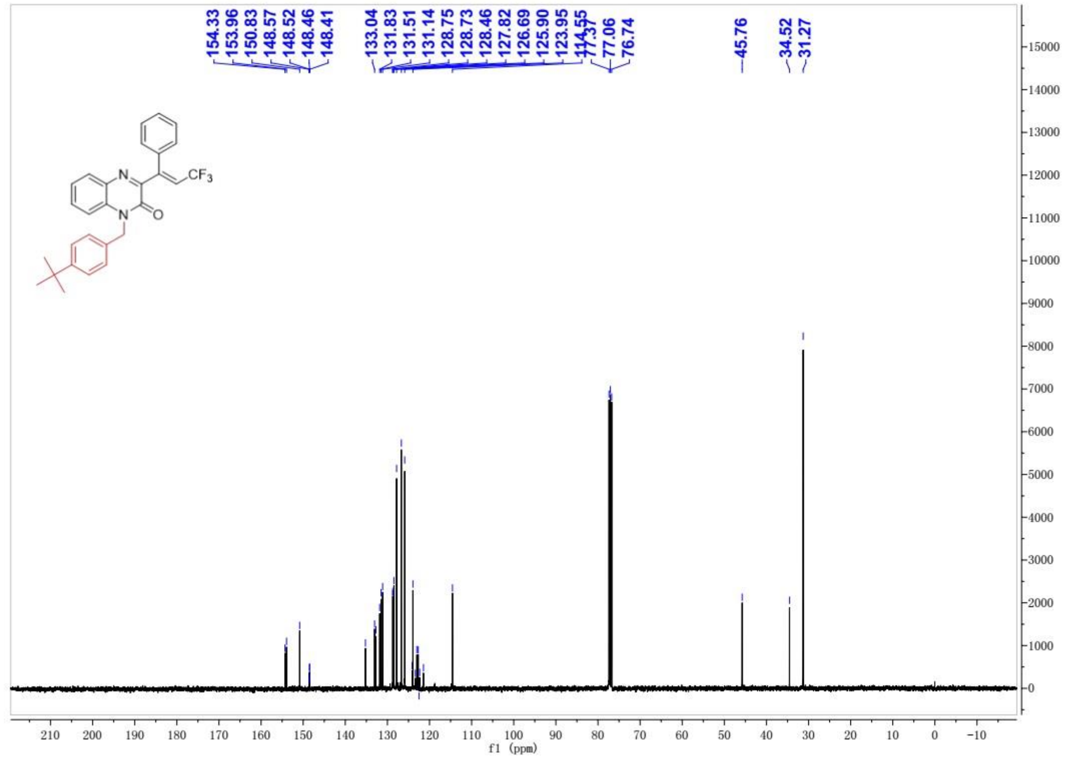

^{1}H and ^{13}C NMR Spectra for **3l**

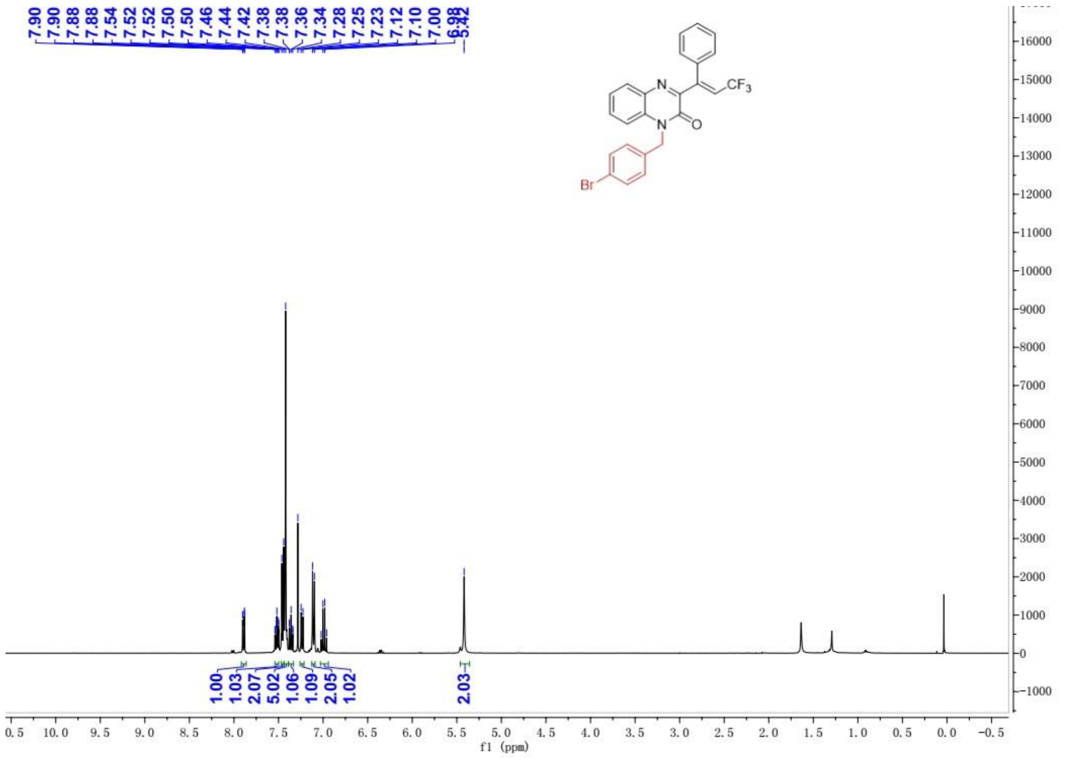

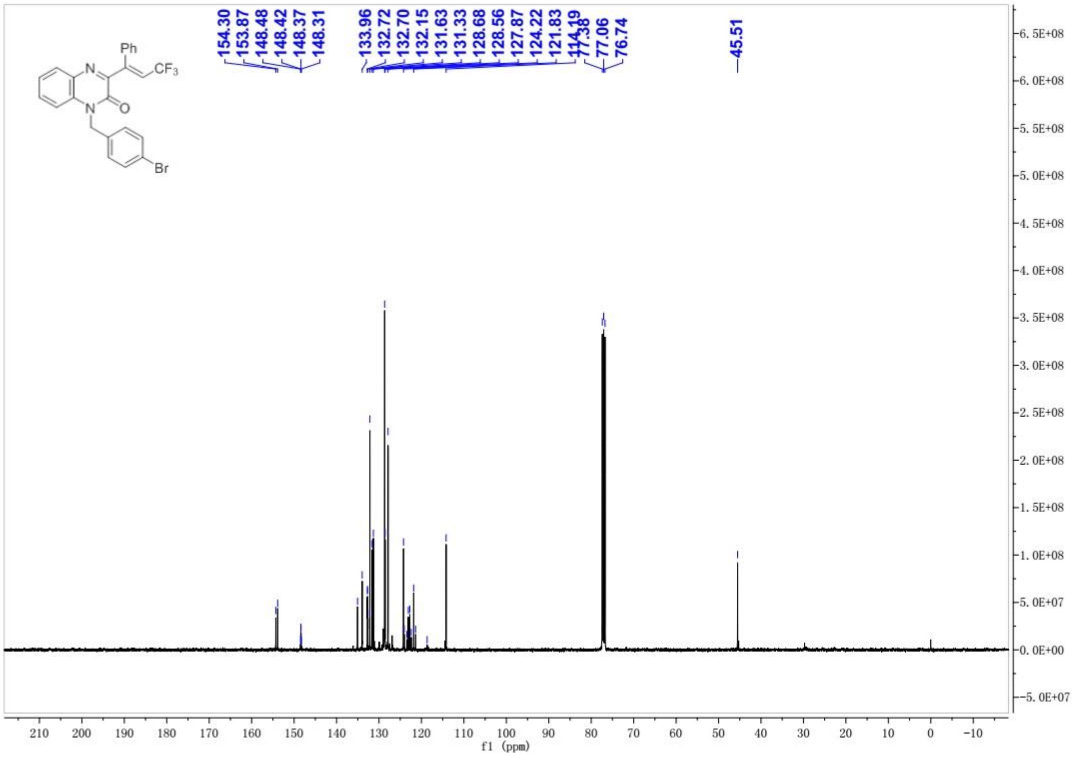

^{1}H and ^{13}C NMR Spectra for **3m**

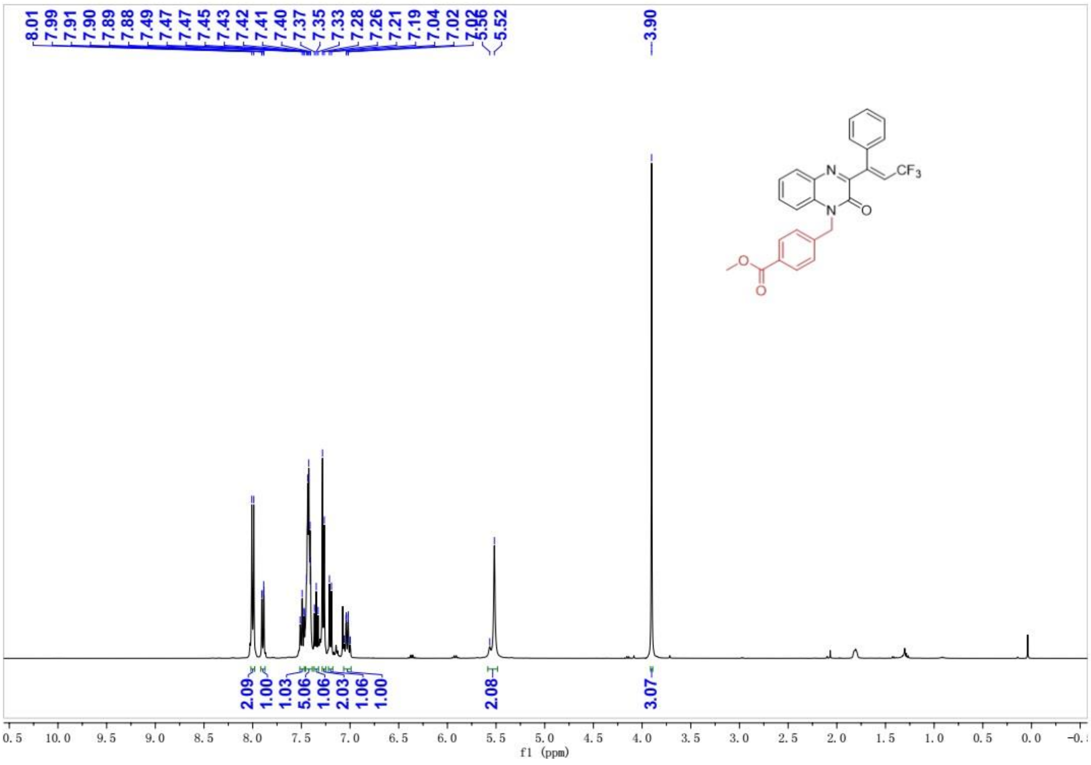

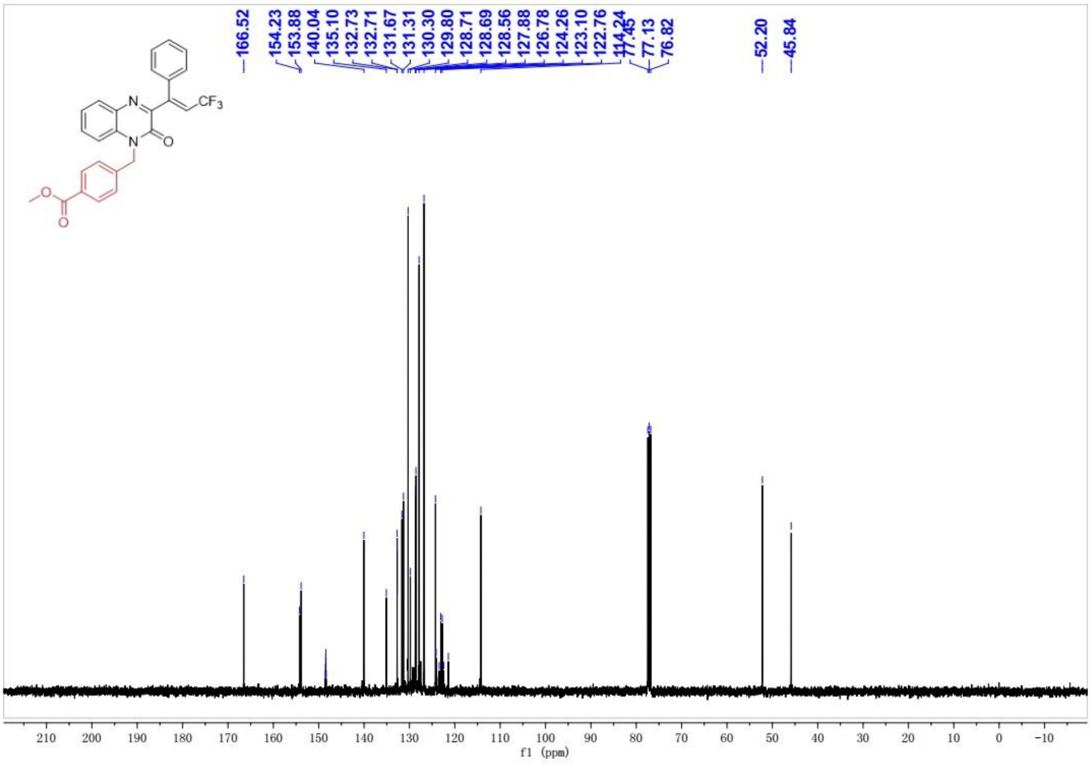

^{1}H and ^{13}C NMR Spectra for **3n**

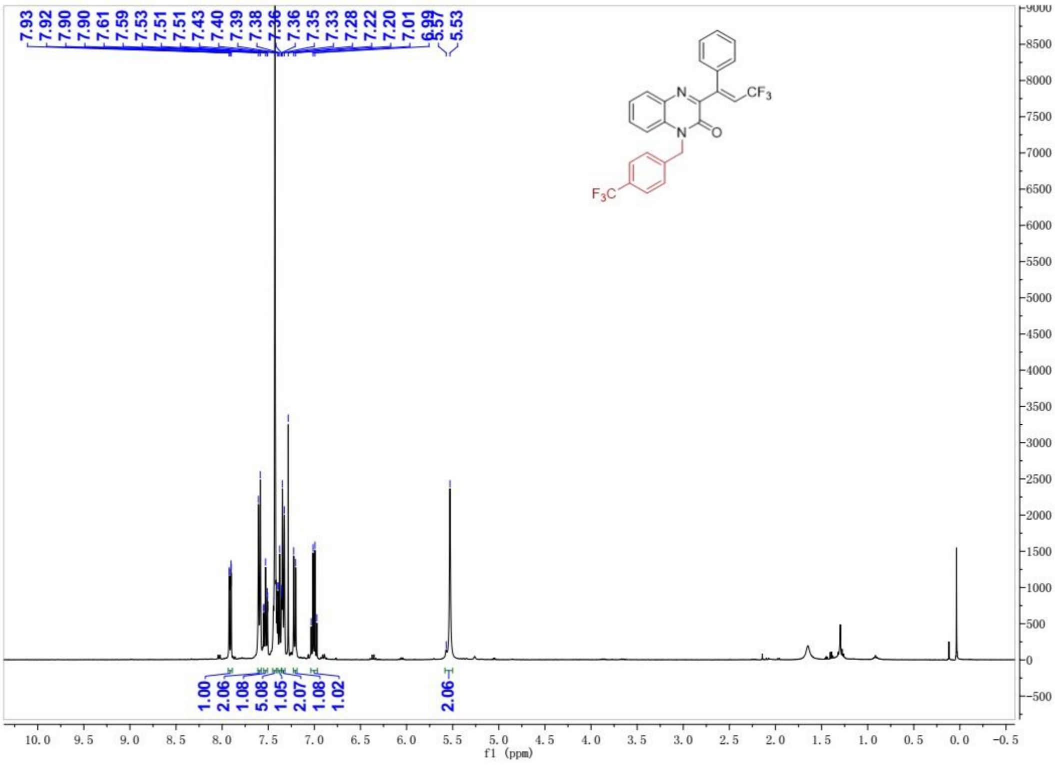

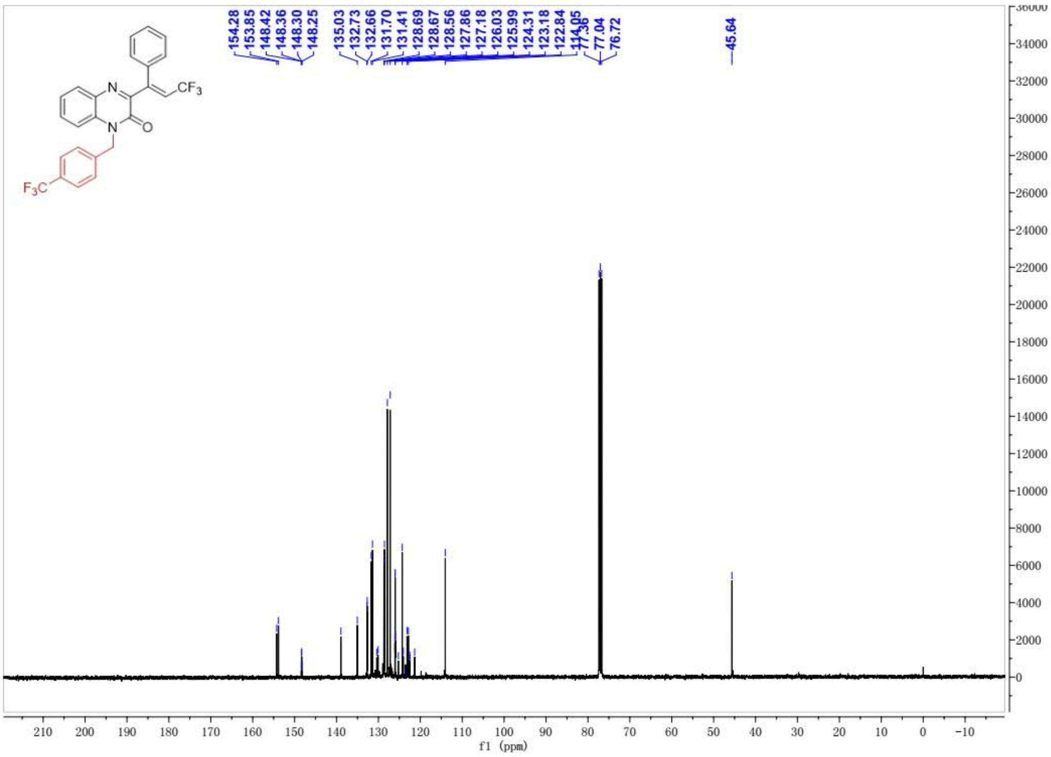

^{1}H and ^{13}C NMR Spectra for **3o**

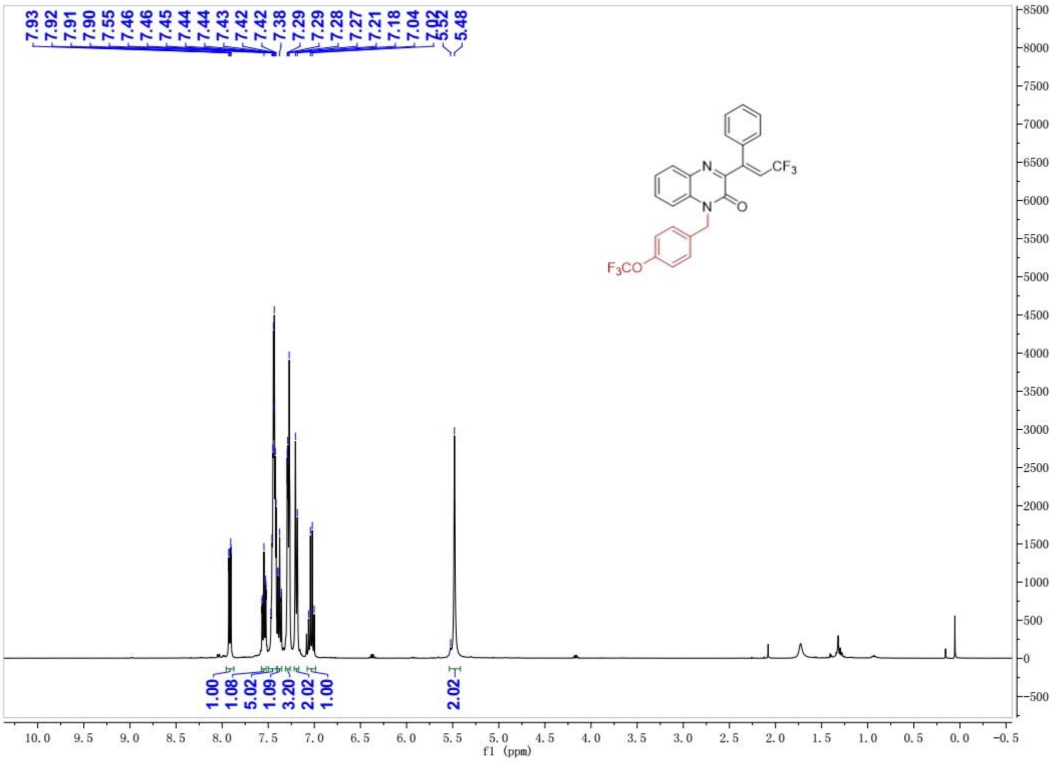

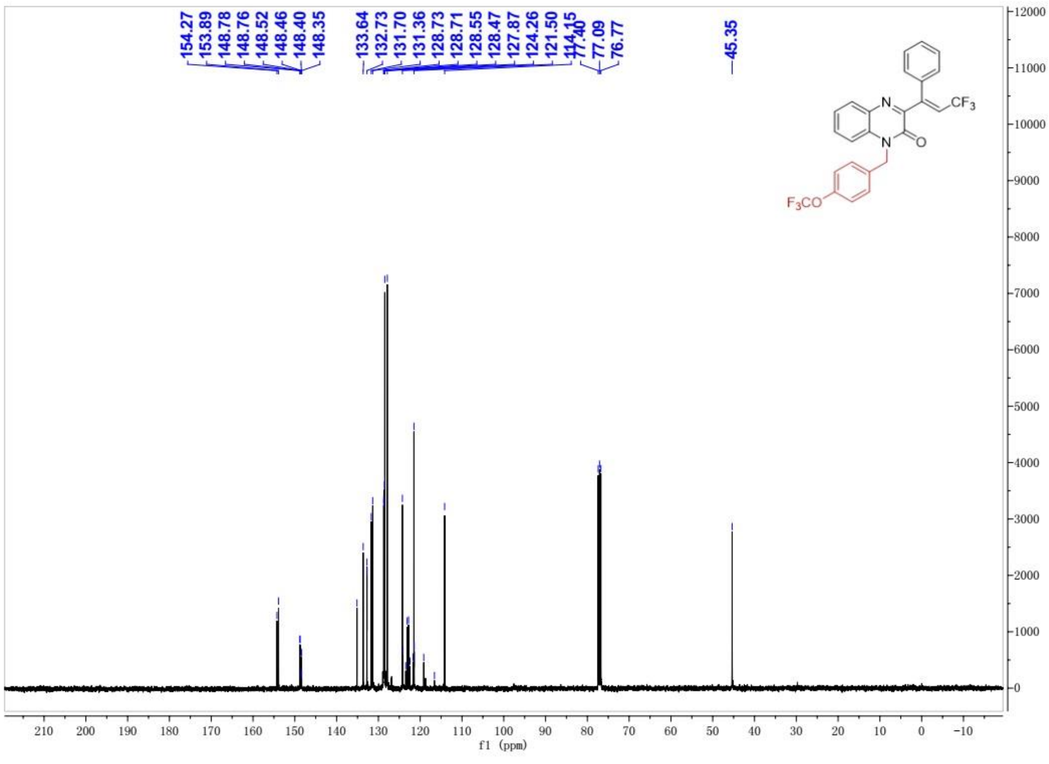

^{1}H and ^{13}C NMR Spectra for **3p**

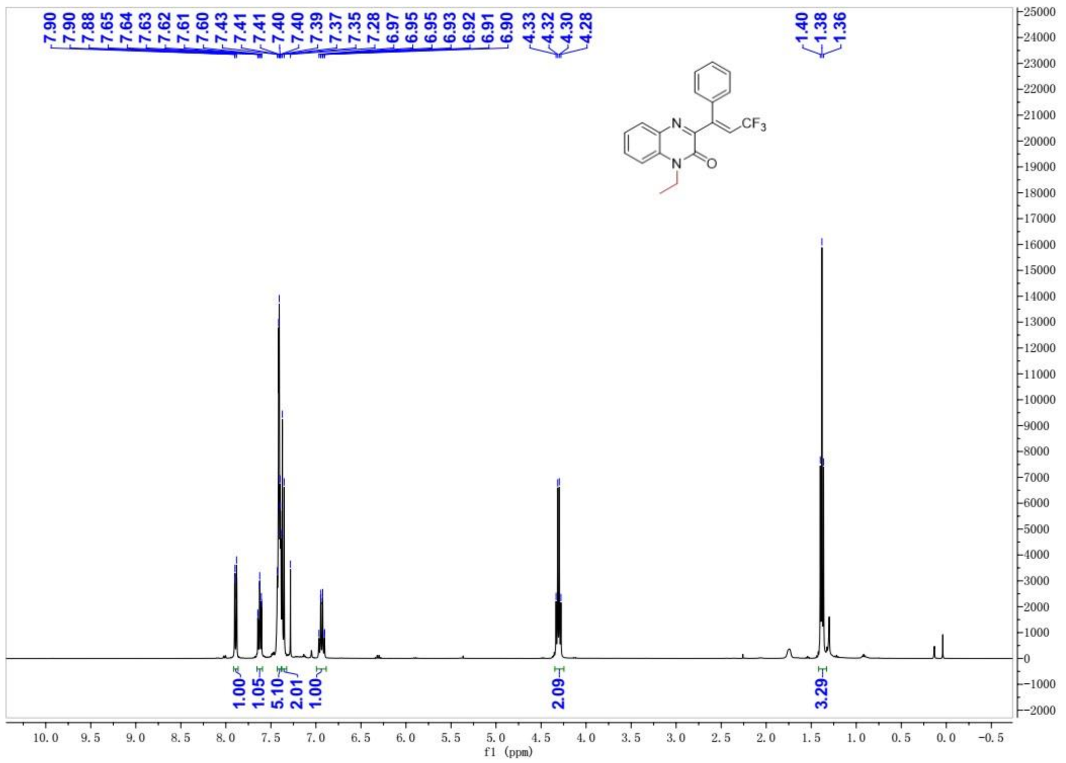

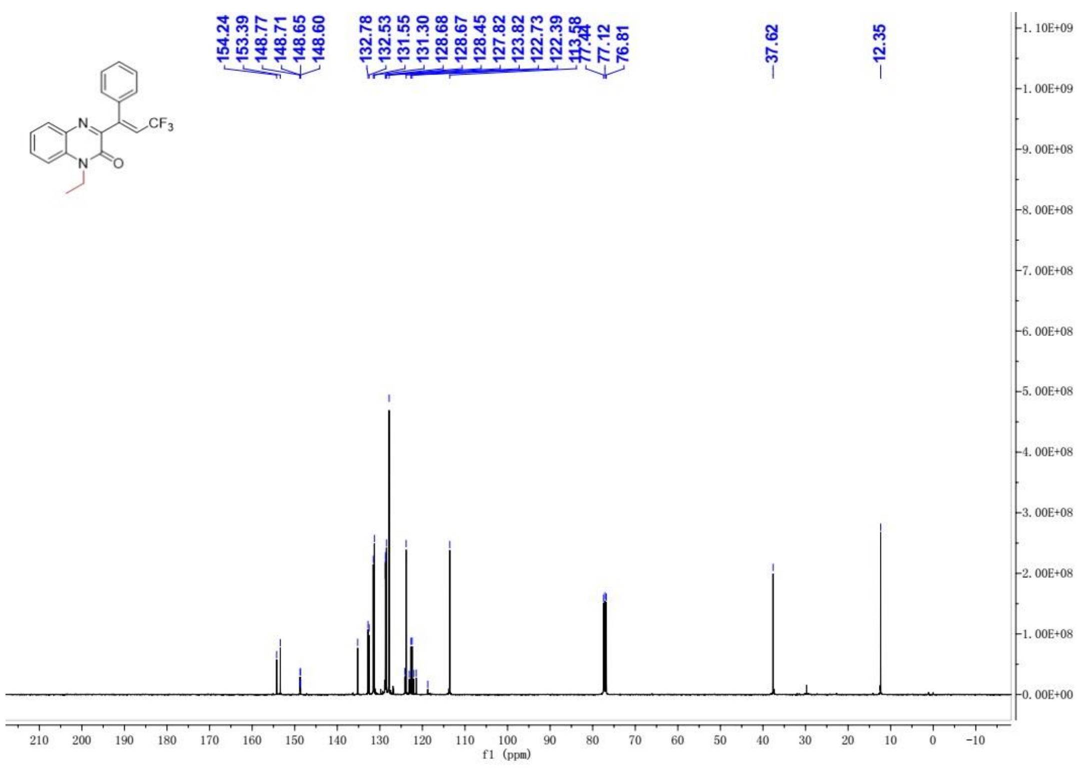

^{1}H and ^{13}C NMR Spectra for **3q**

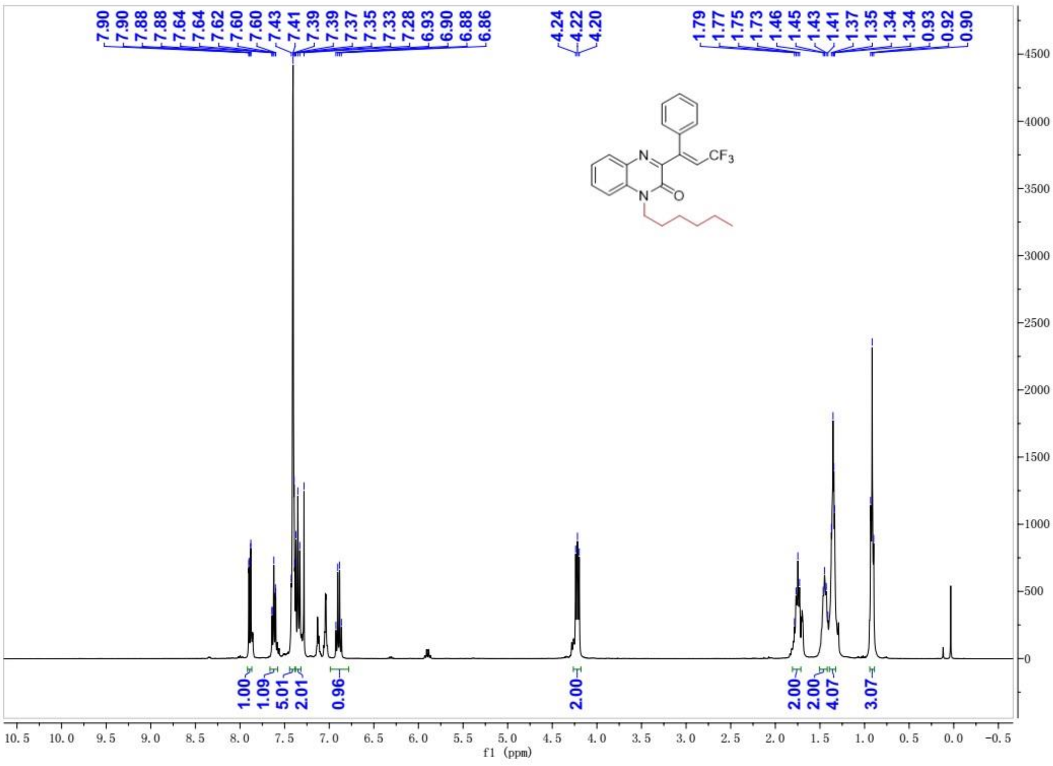

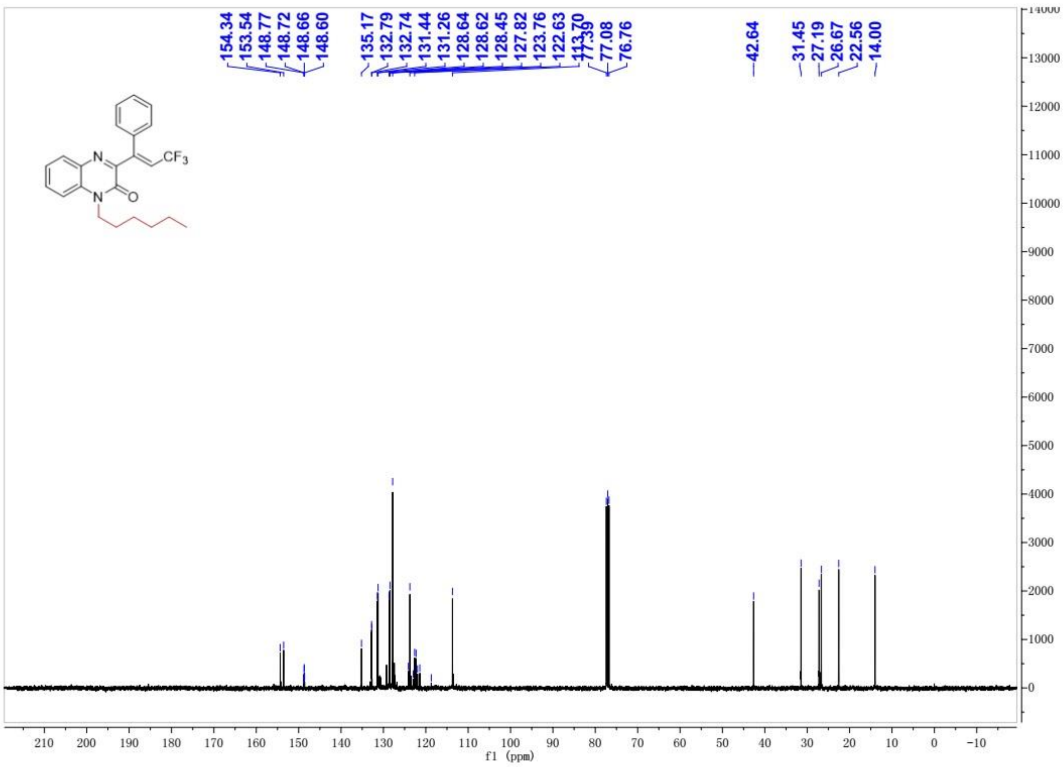

^{1}H and ^{13}C NMR Spectra for **3r**

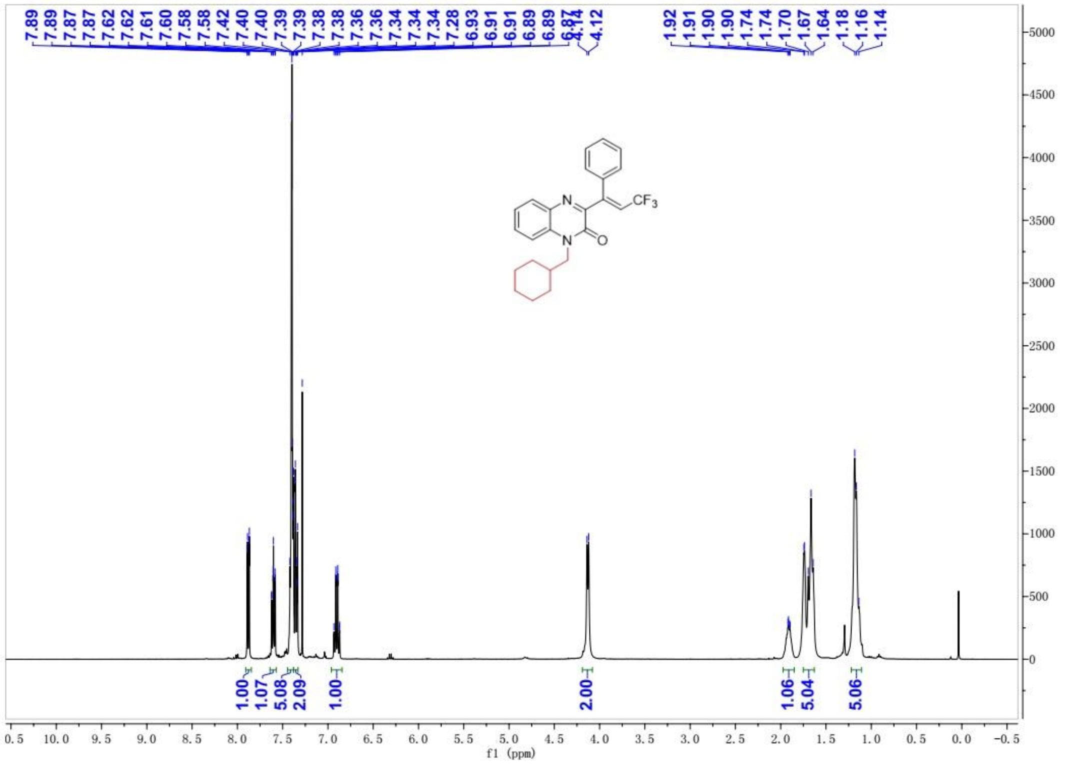

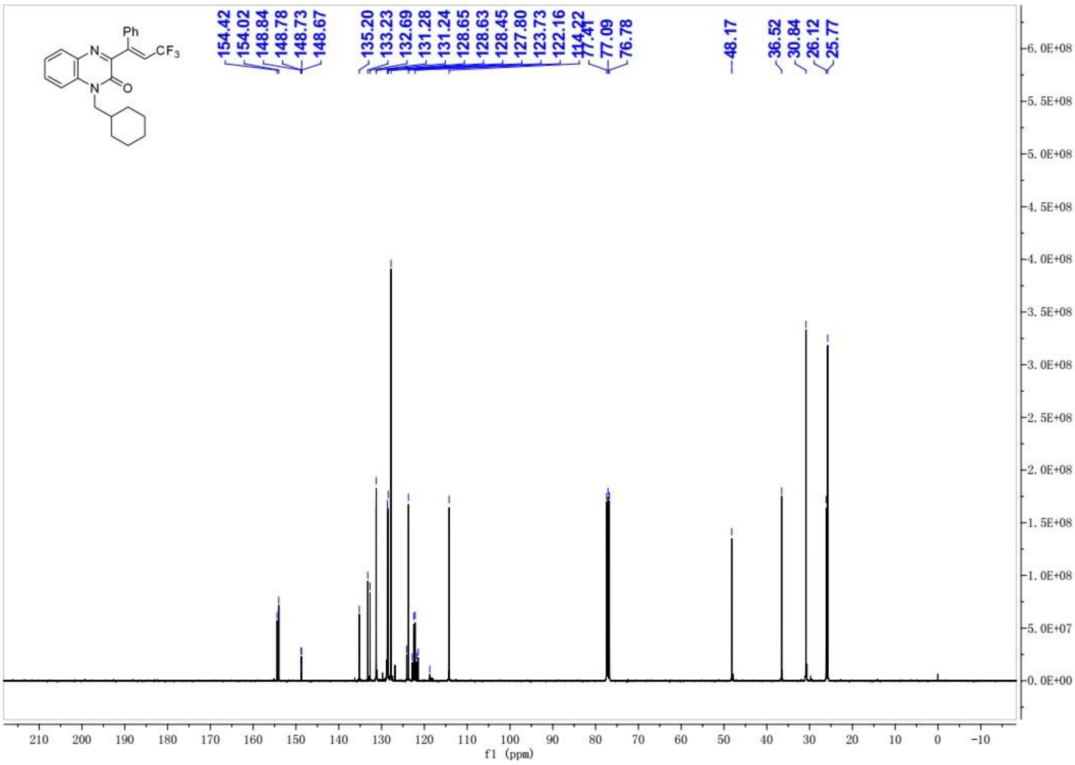

^{1}H and ^{13}C NMR Spectra for **3s**

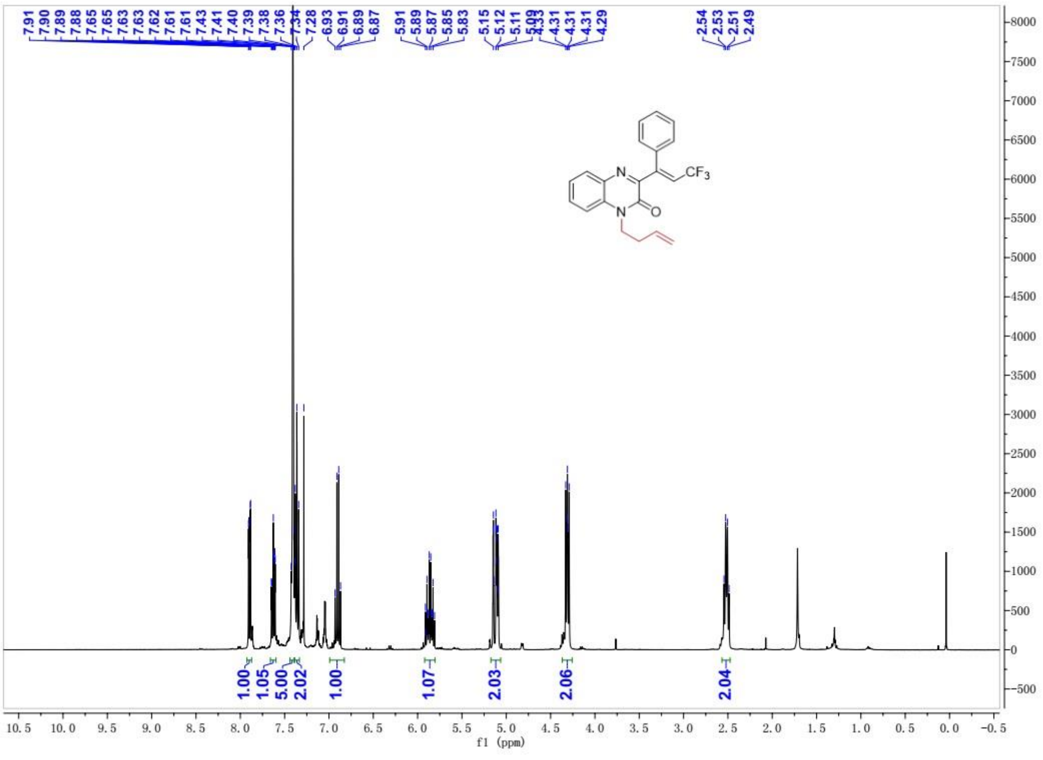

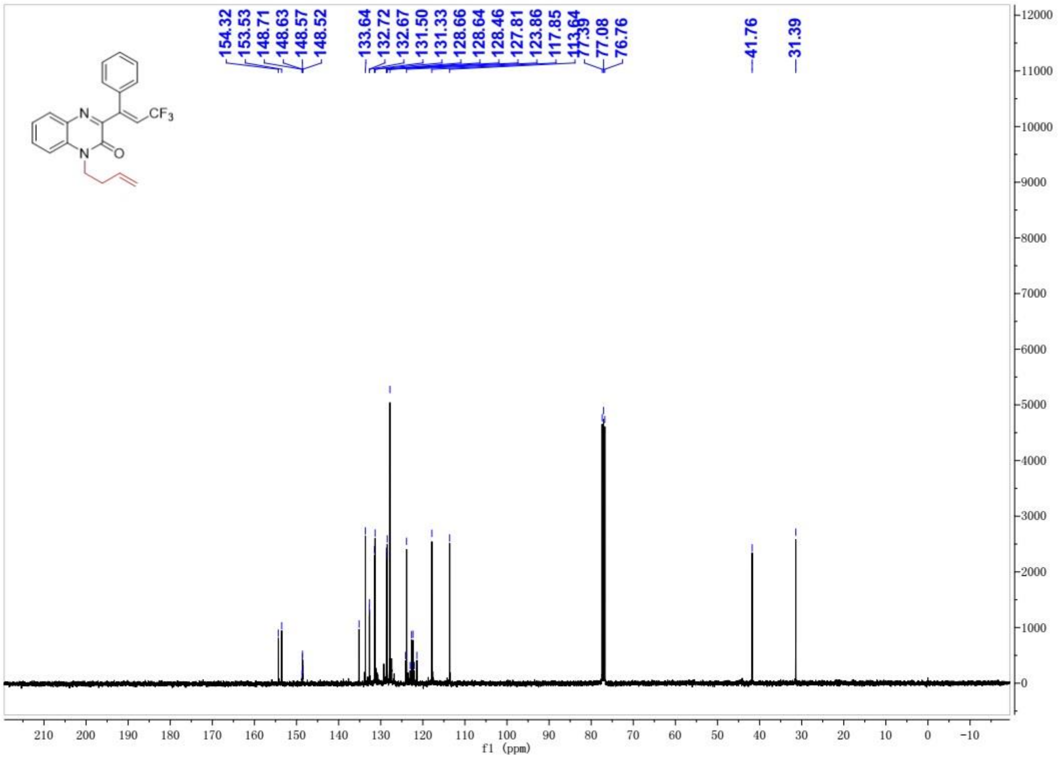

^{1}H and ^{13}C NMR Spectra for **3t**

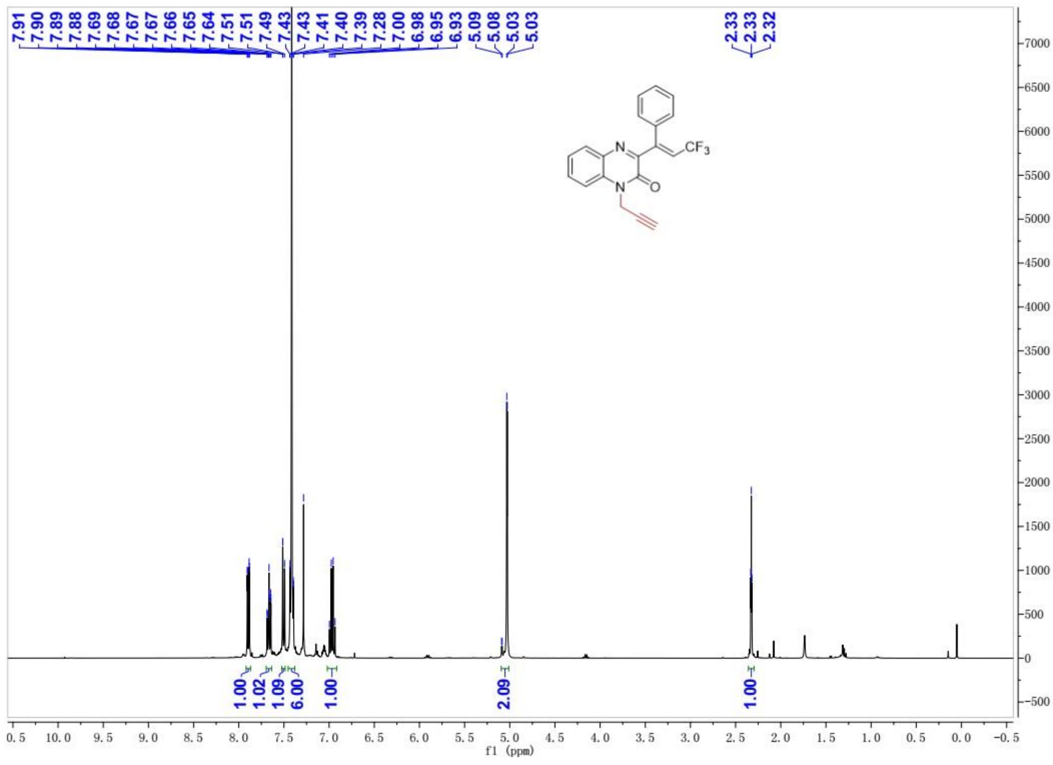

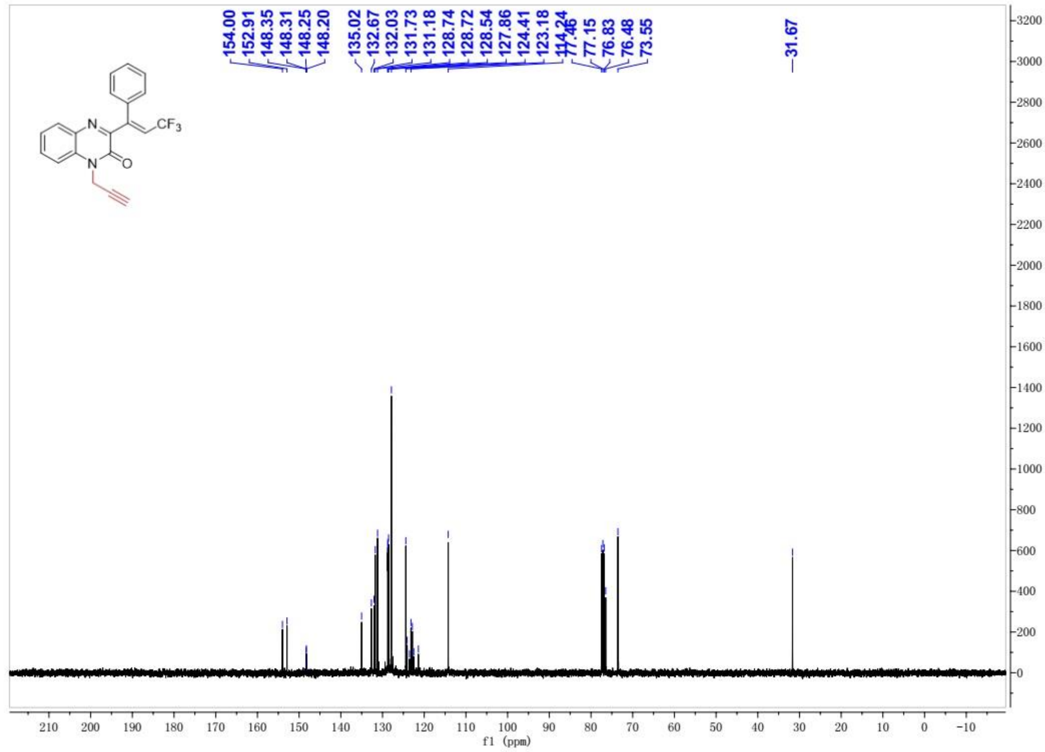

^{1}H and ^{13}C NMR Spectra for **3u**

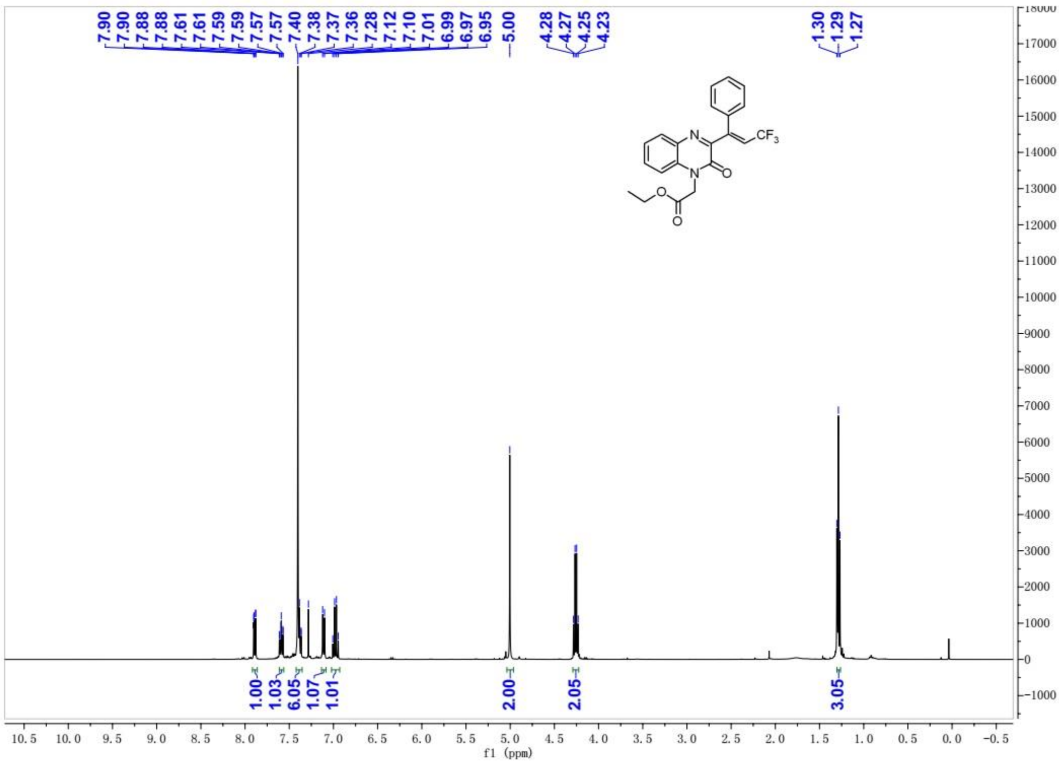

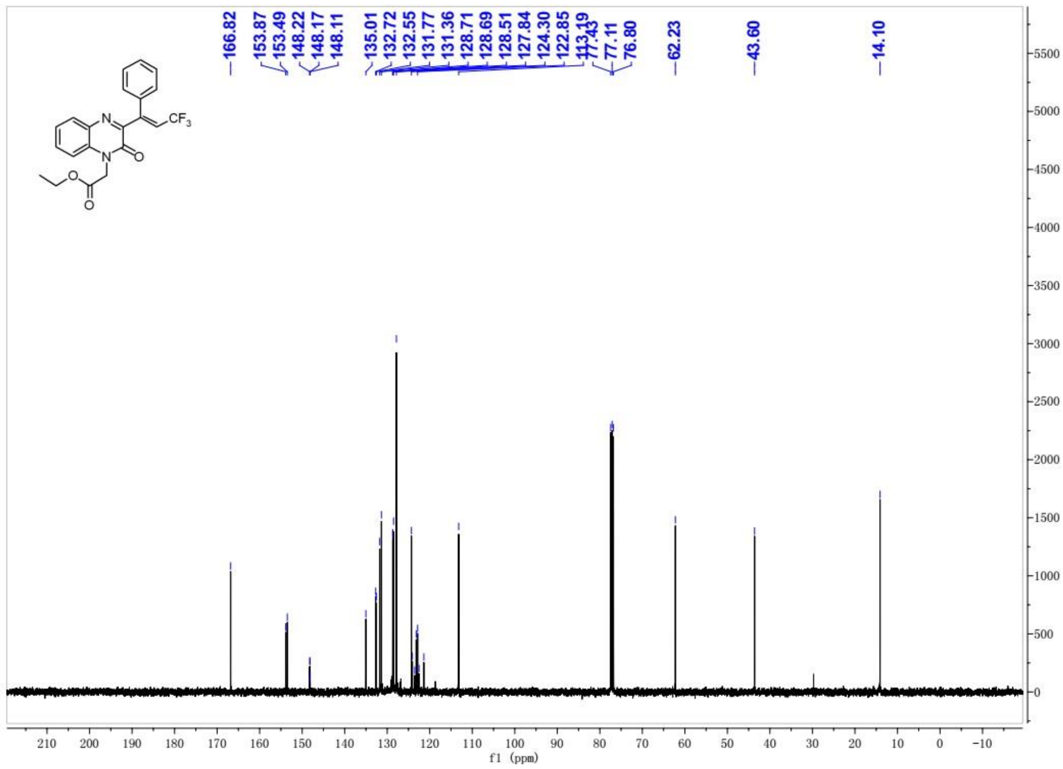

^{1}H and ^{13}C NMR Spectra for **3v**

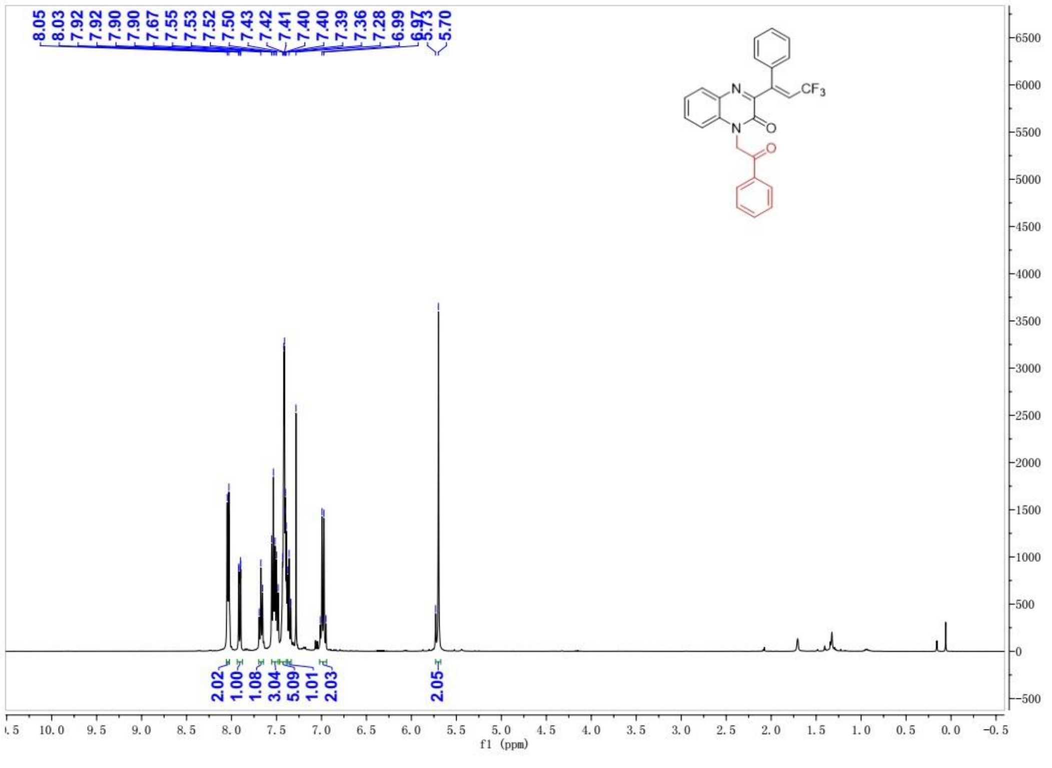

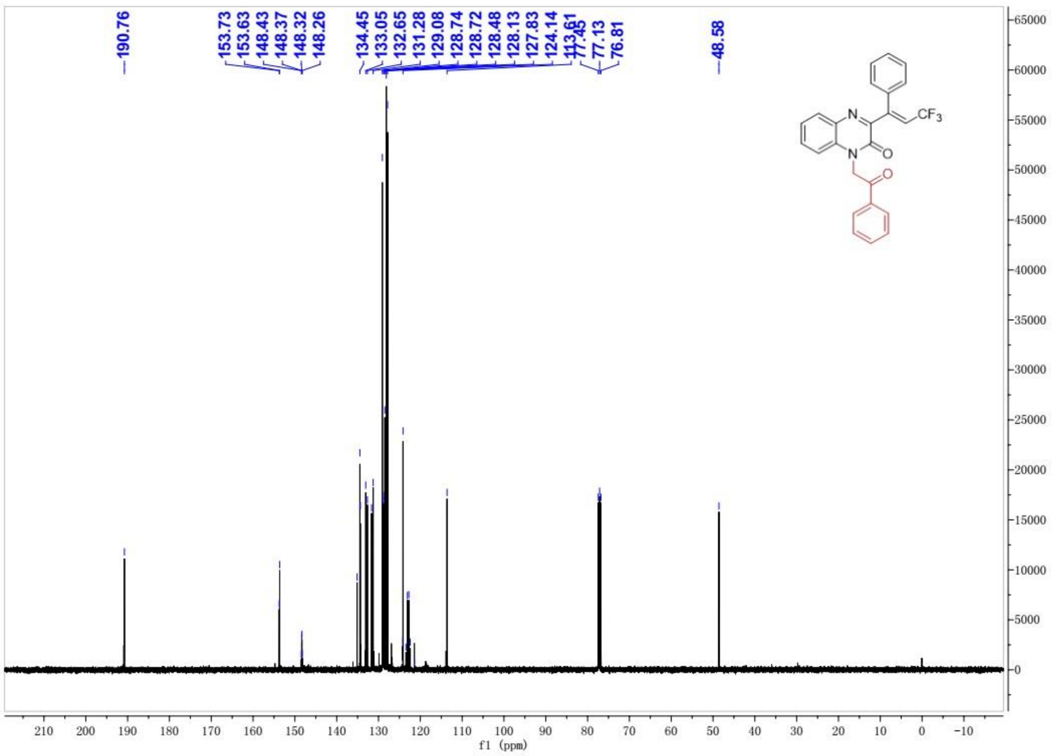

^{1}H and ^{13}C NMR Spectra for **3w**

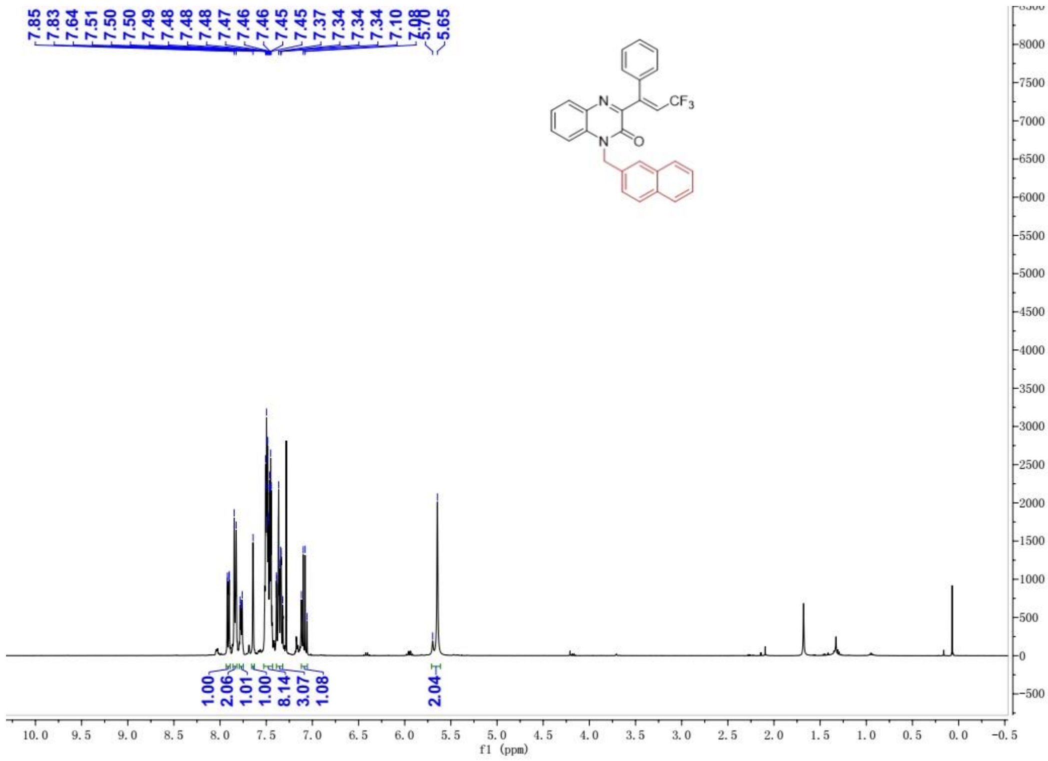

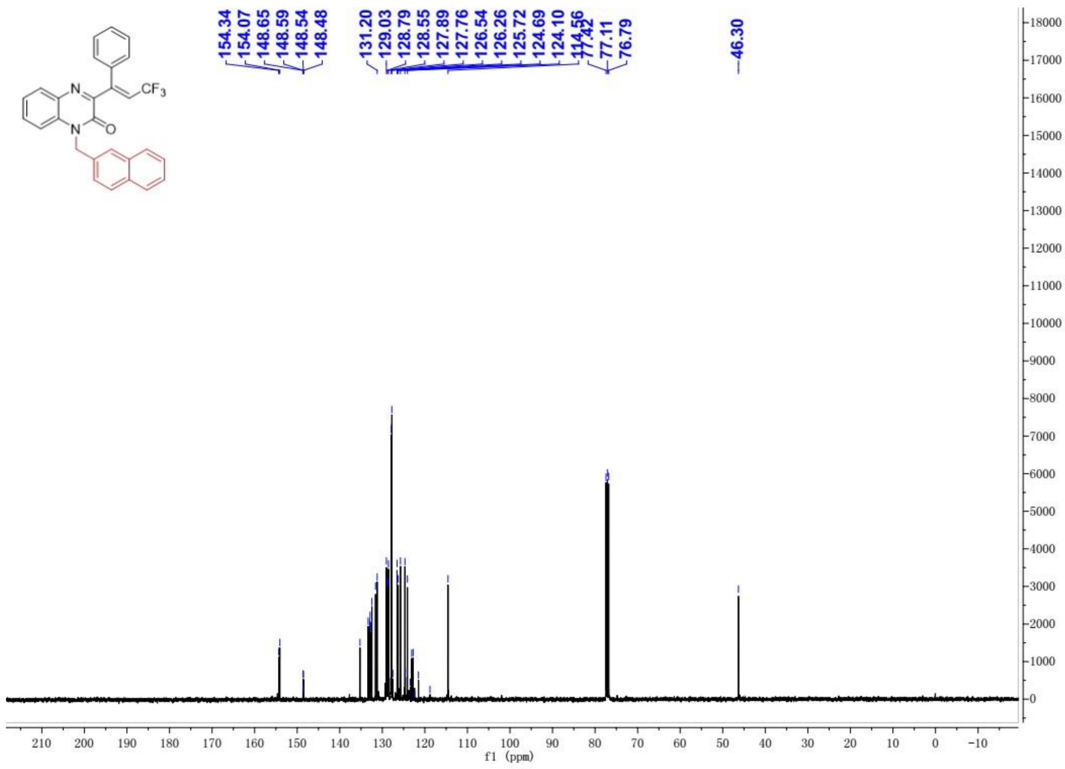

^{1}H and ^{13}C NMR Spectra for **3x**

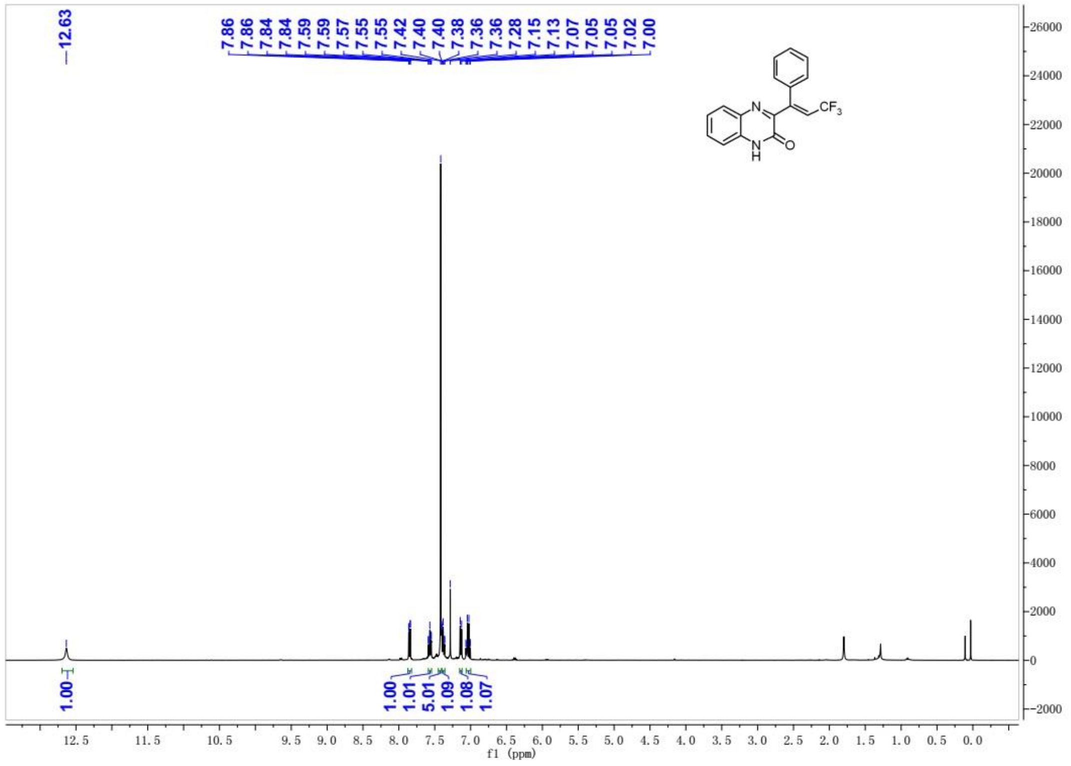

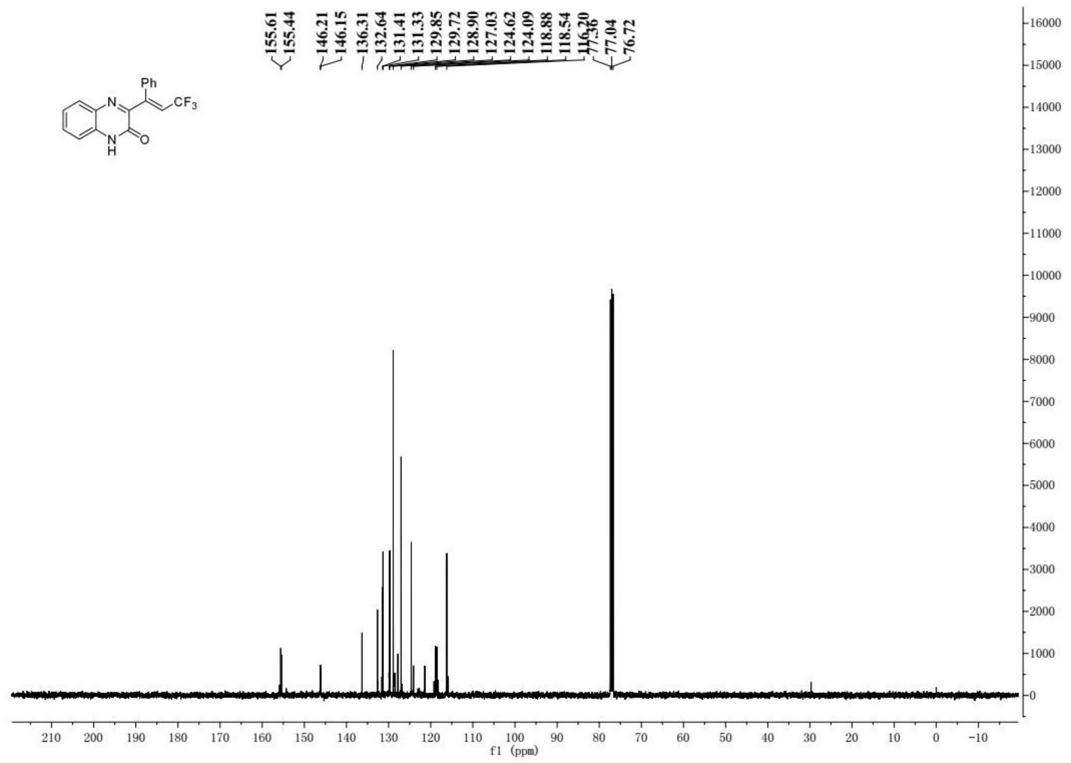

^{1}H and ^{13}C NMR Spectra for **3y**

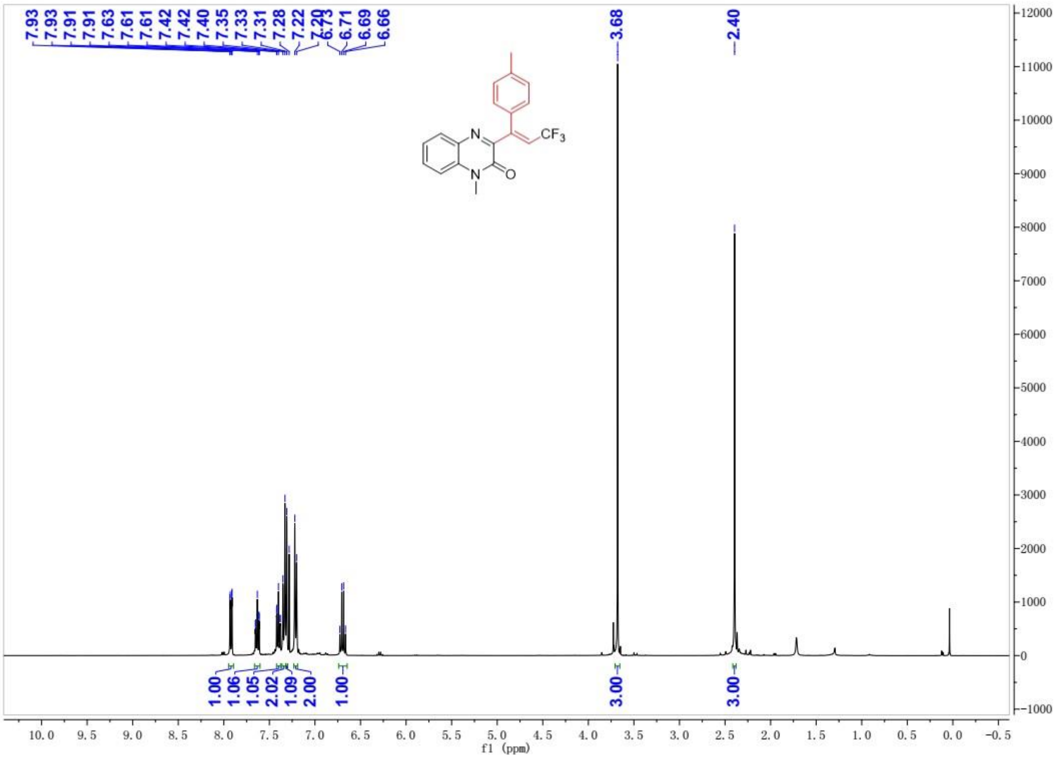

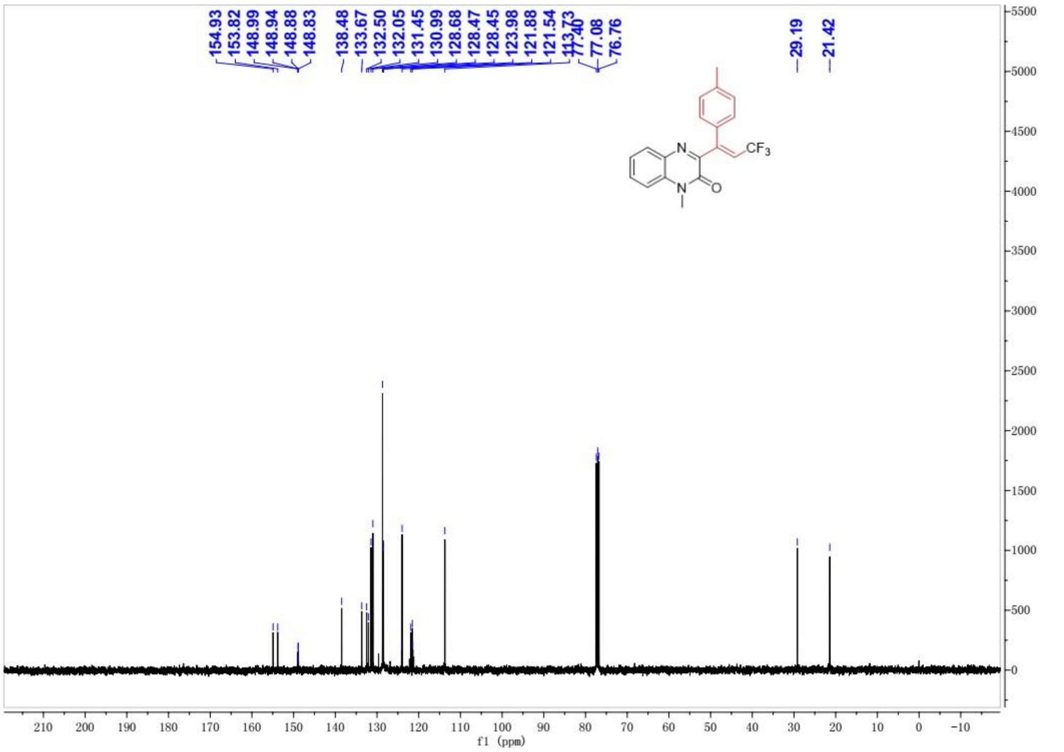

^{1}H and ^{13}C NMR Spectra for **3z**

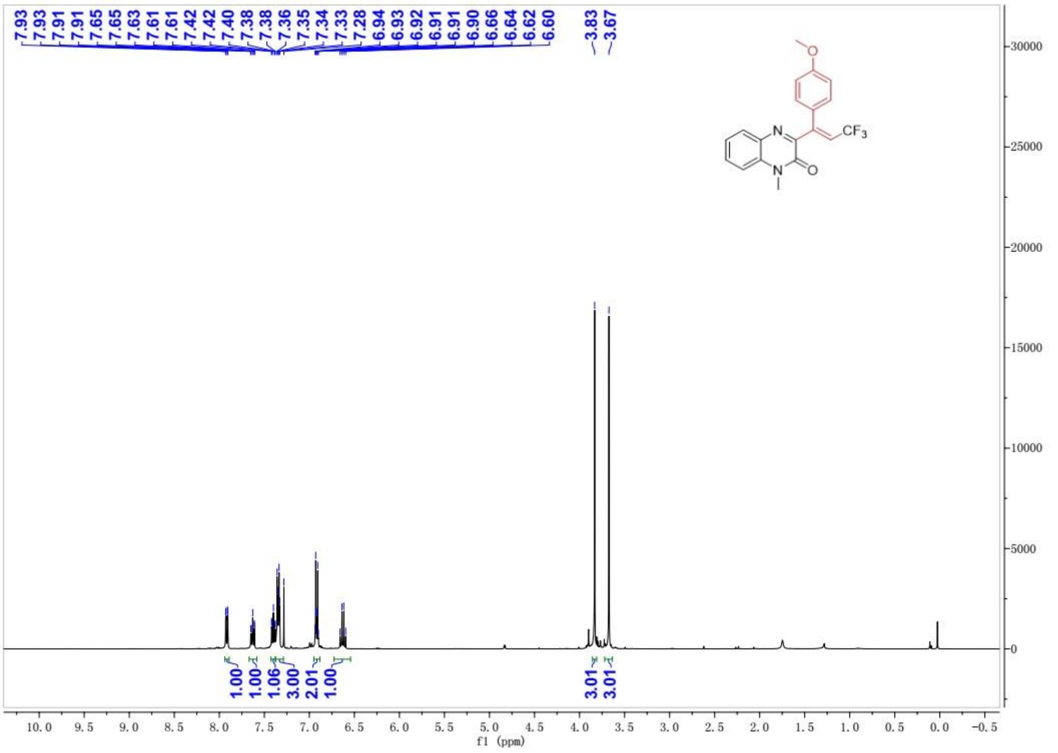

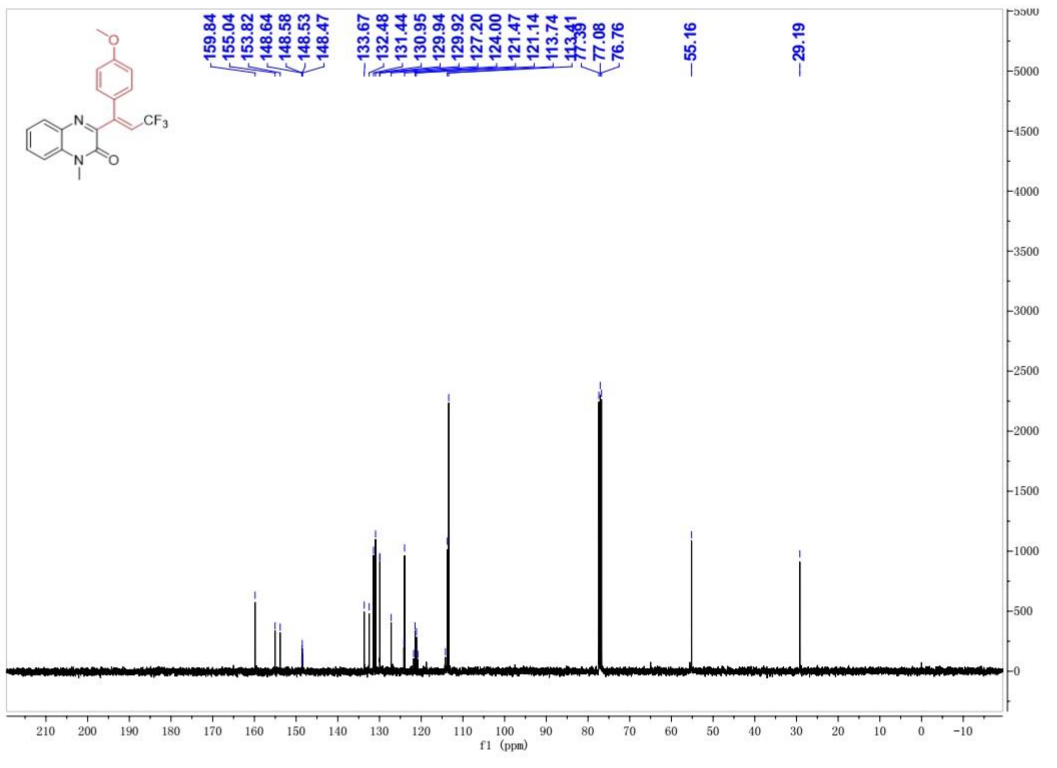

^{1}H and ^{13}C NMR Spectra for **3aa**

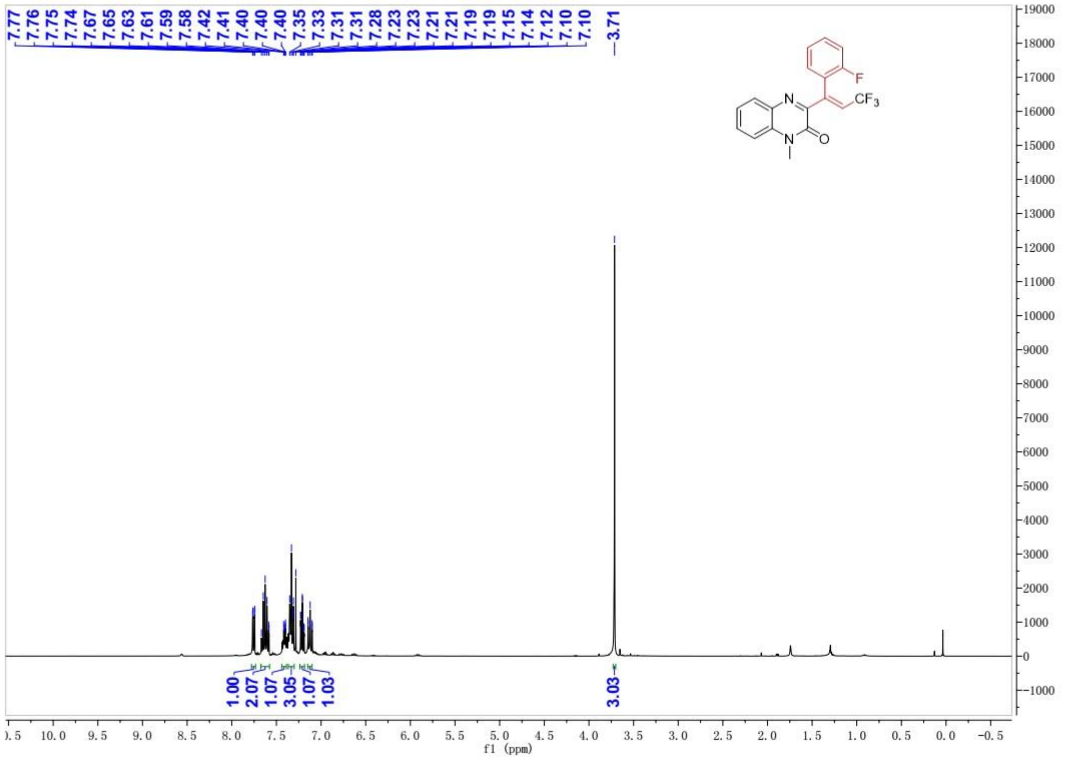

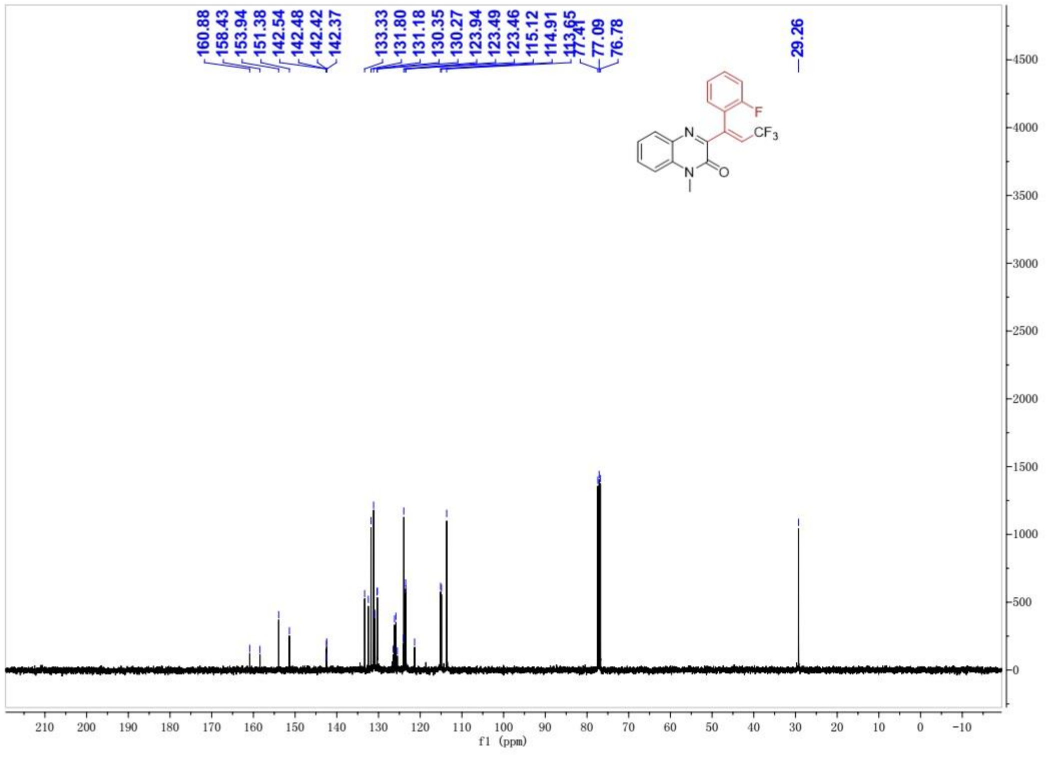

^{1}H and ^{13}C NMR Spectra for **3ab**

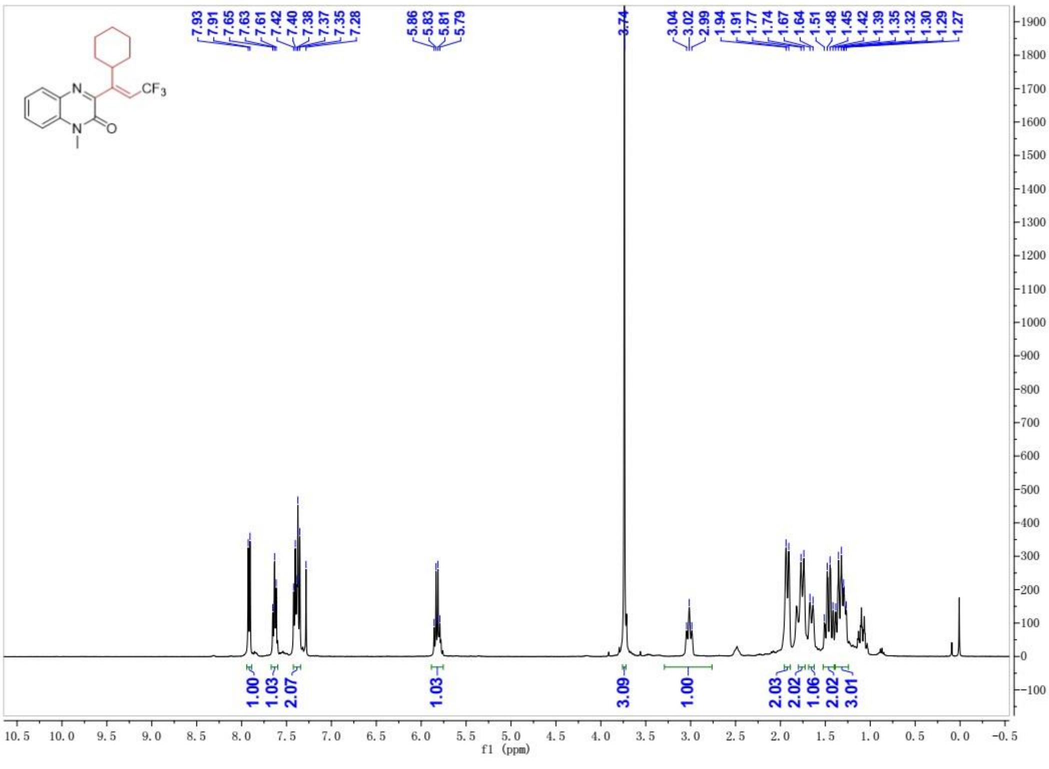

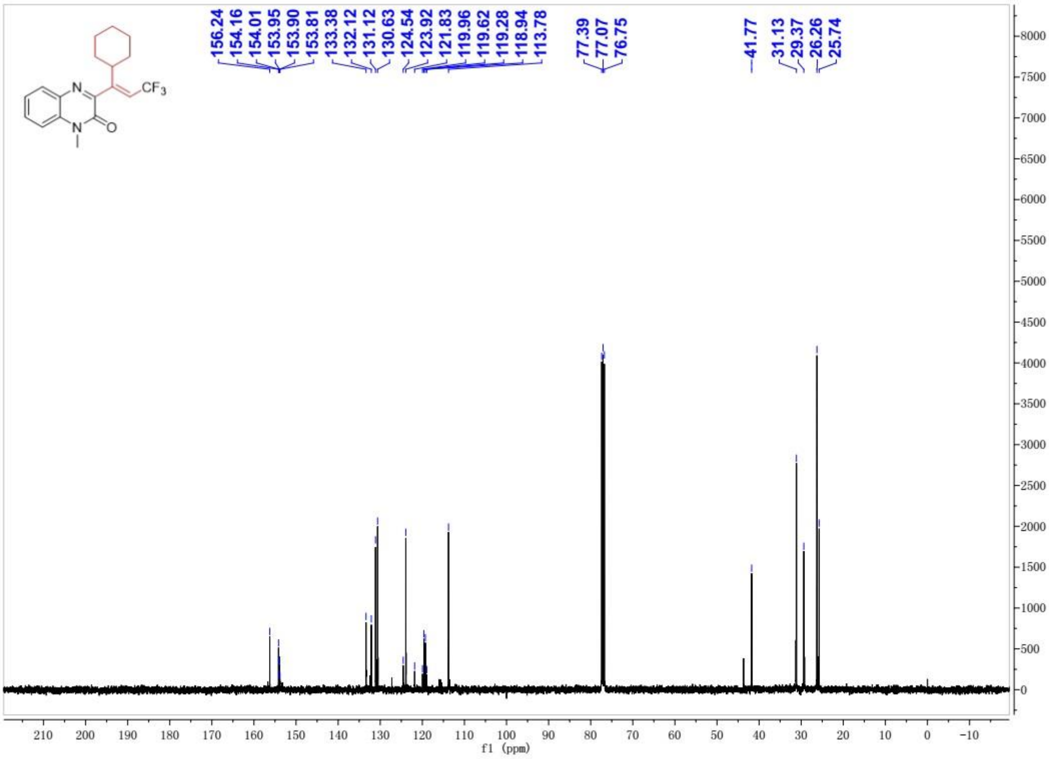

^{1}H and ^{13}C NMR Spectra for **3ac**

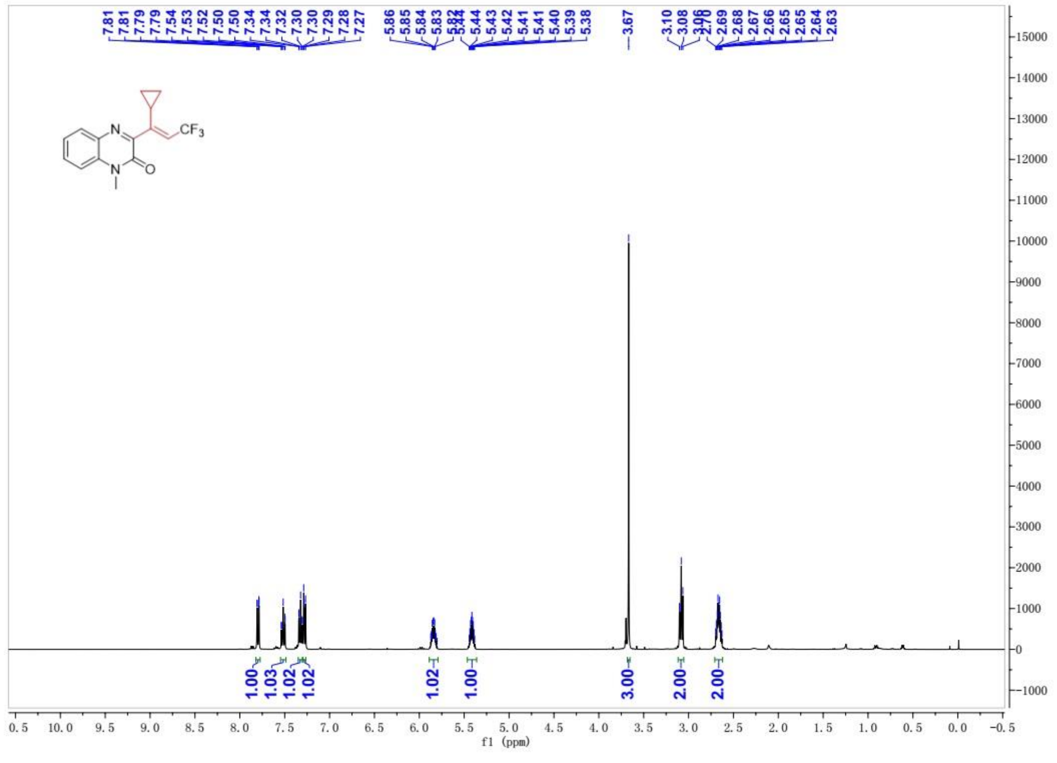

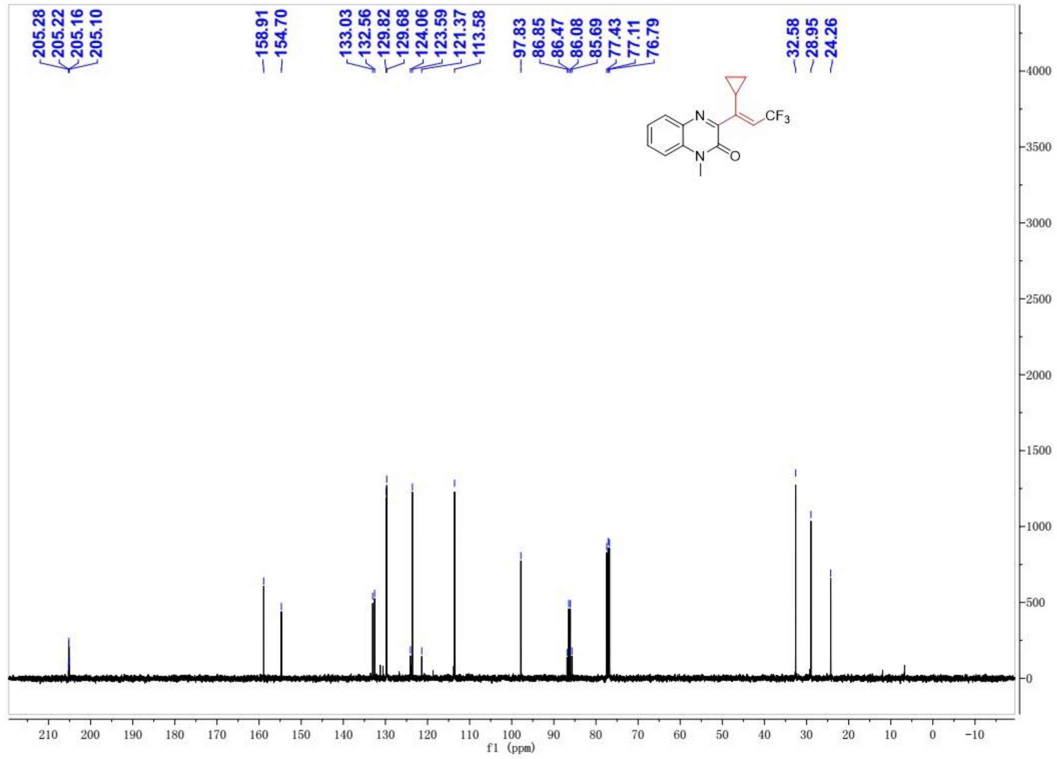

^{1}H and ^{13}C NMR Spectra for **3ad**

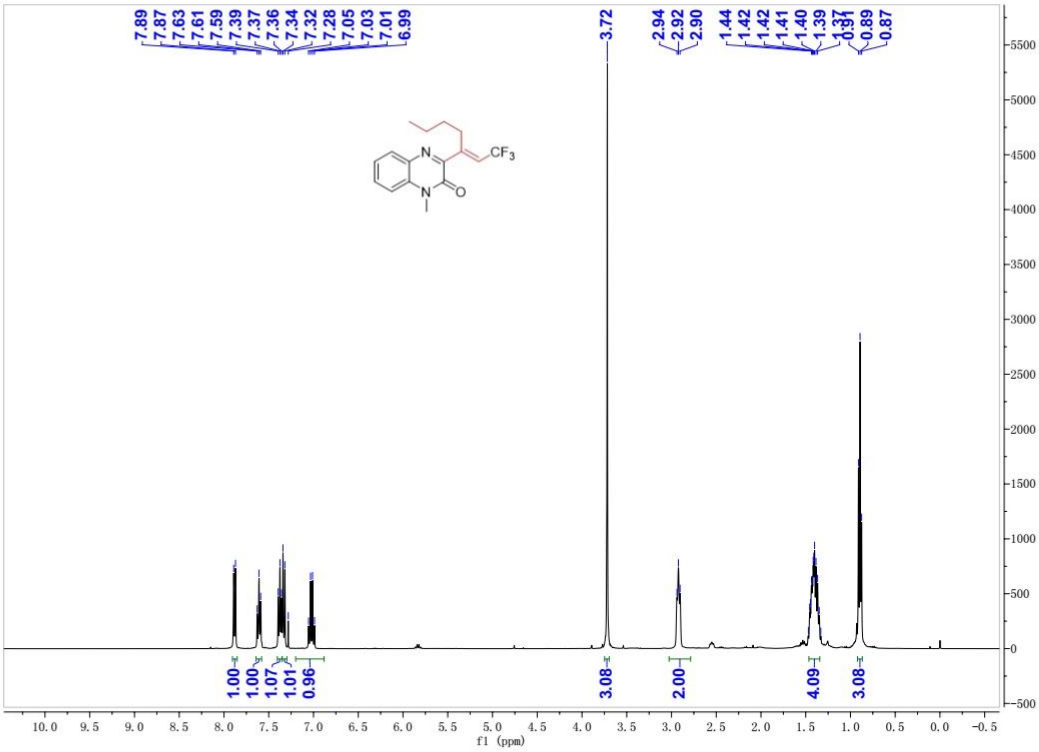

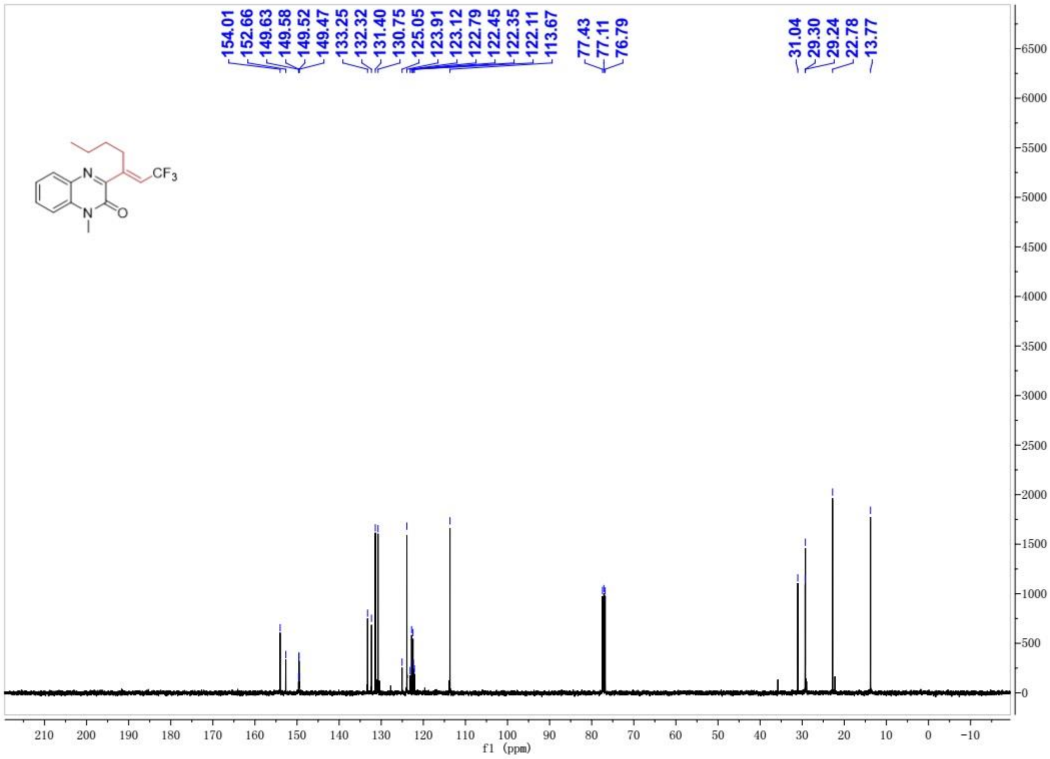

^{1}H and ^{13}C NMR Spectra for **3ae**

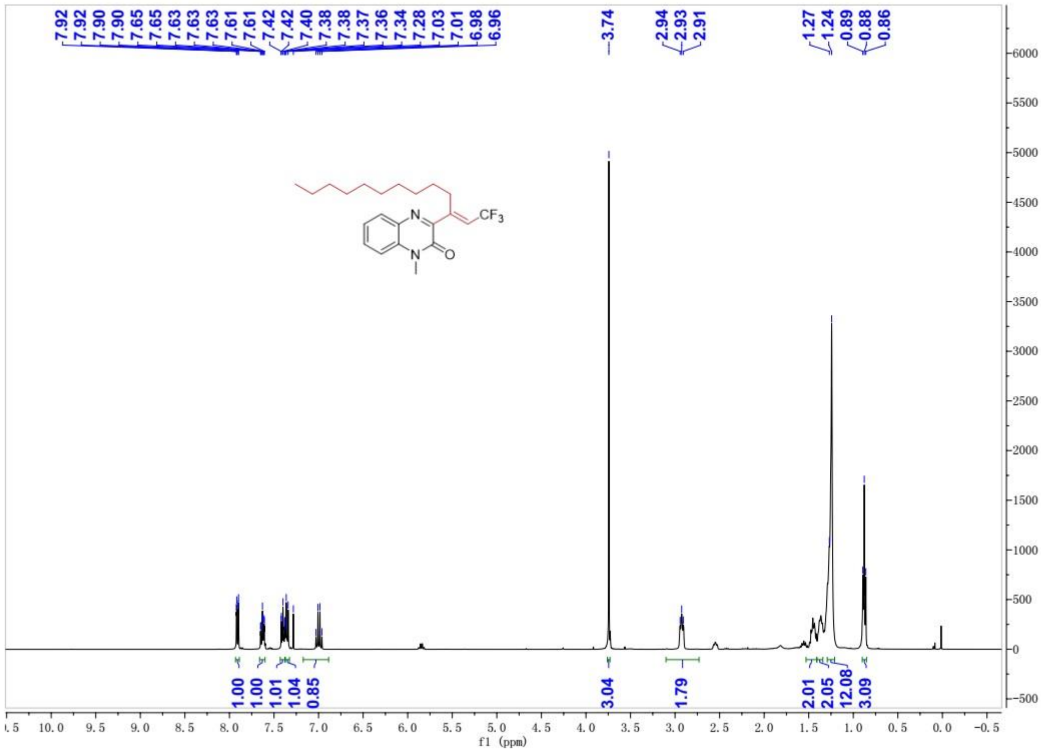

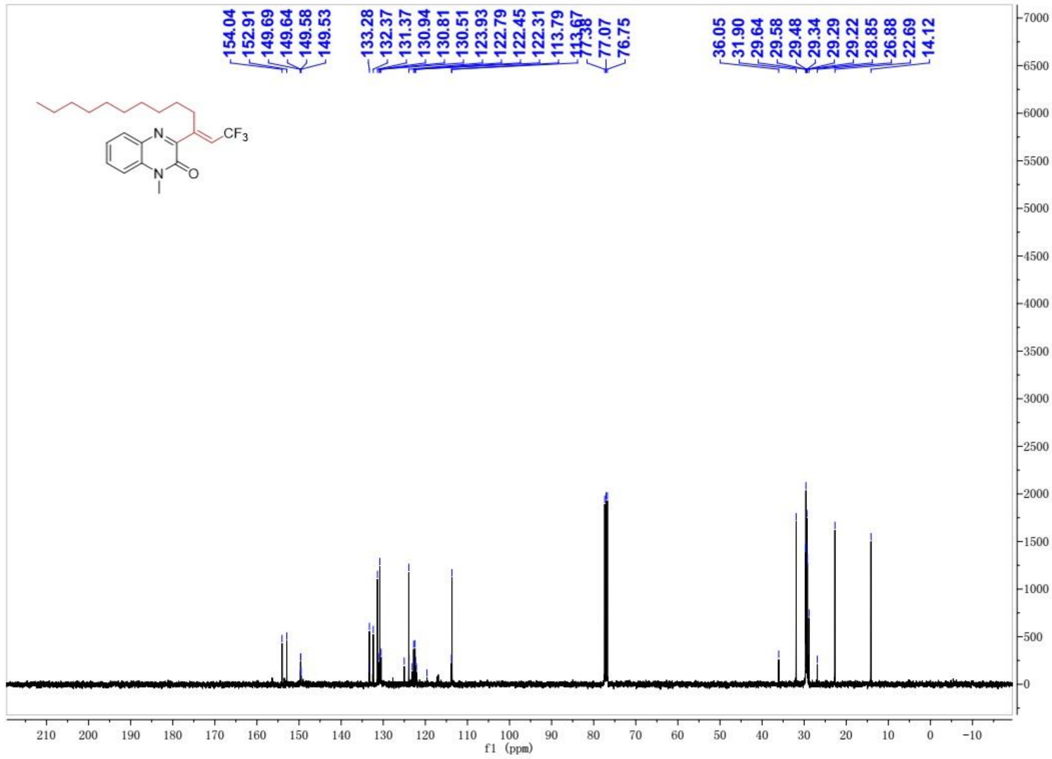

^{1}H and ^{13}C NMR Spectra for **3af**

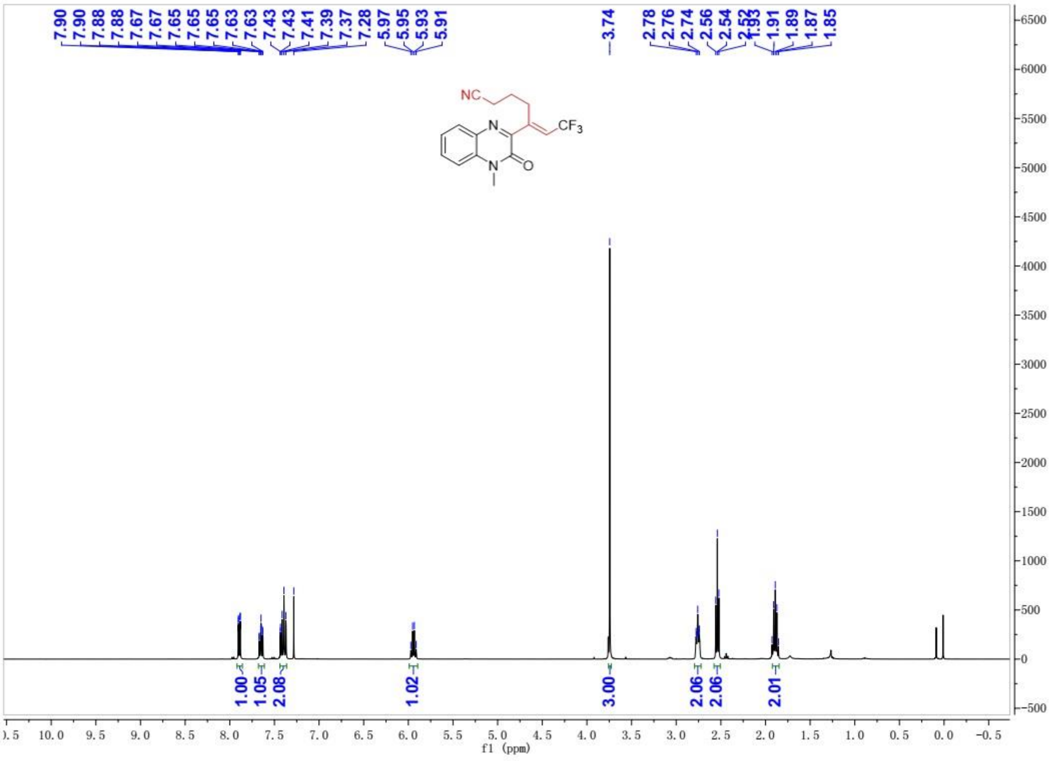

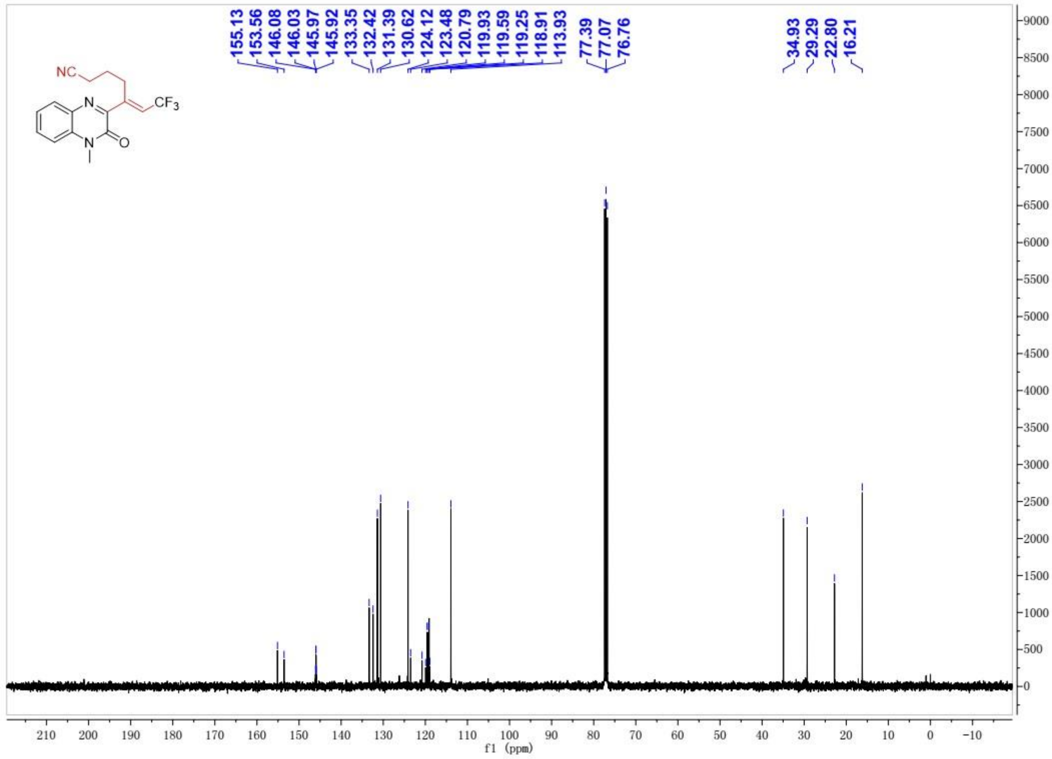

^{1}H and ^{13}C NMR Spectra for **3ag**

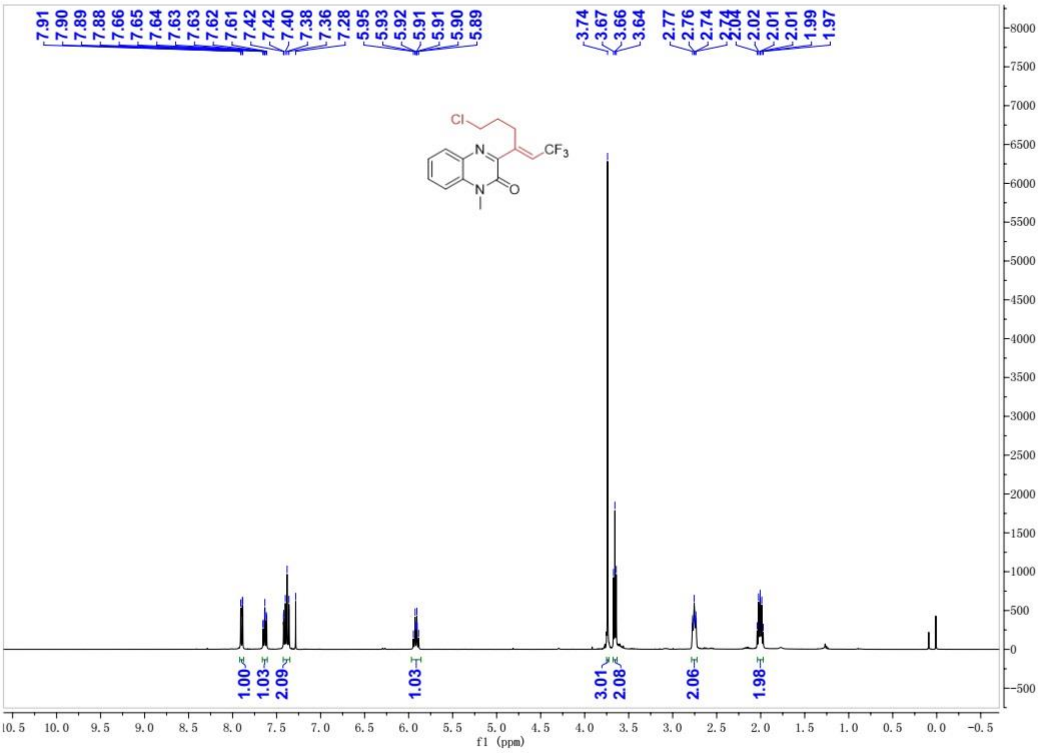

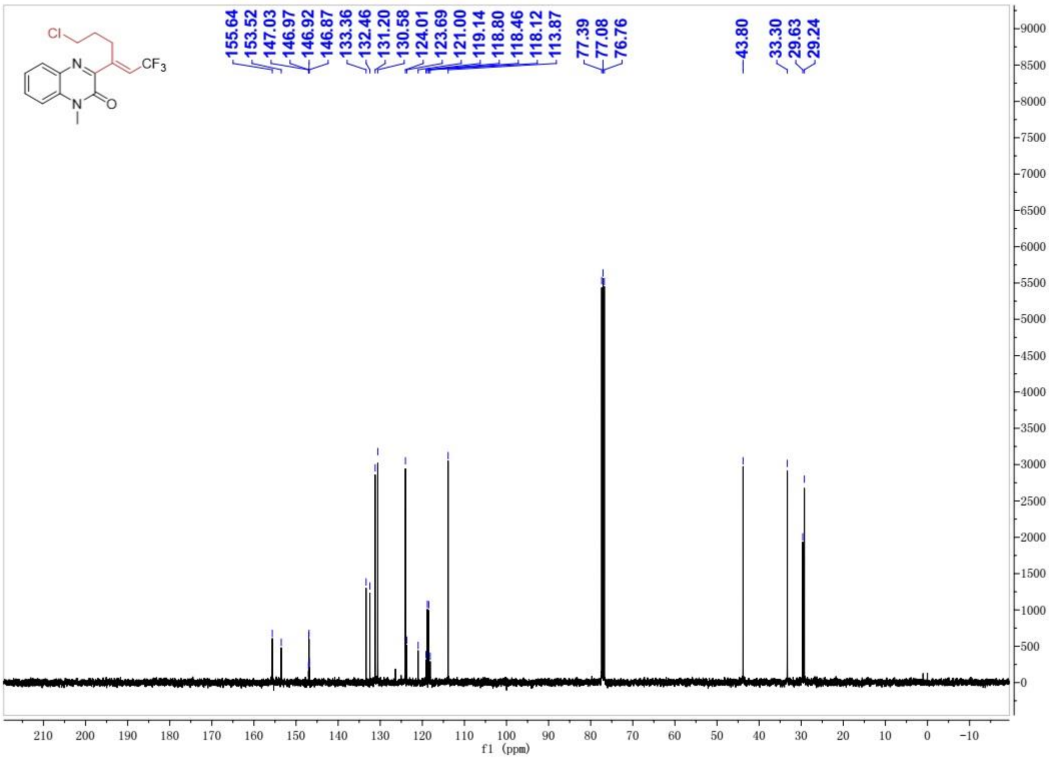

^{1}H and ^{13}C NMR Spectra for **3ah**

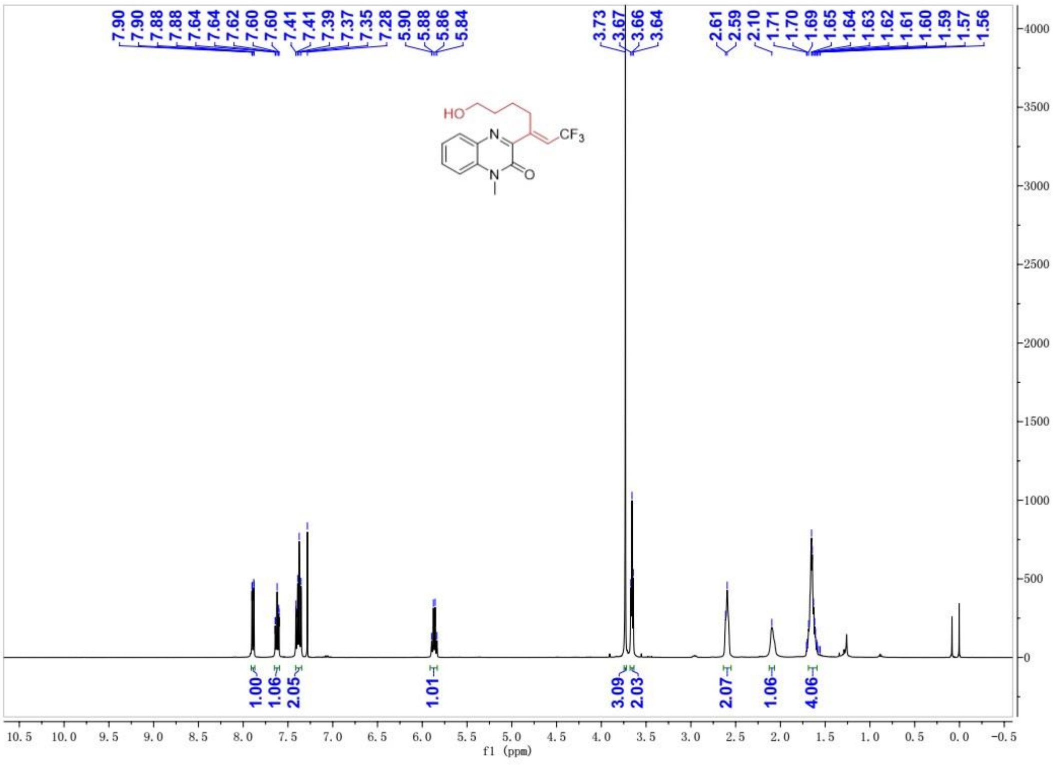

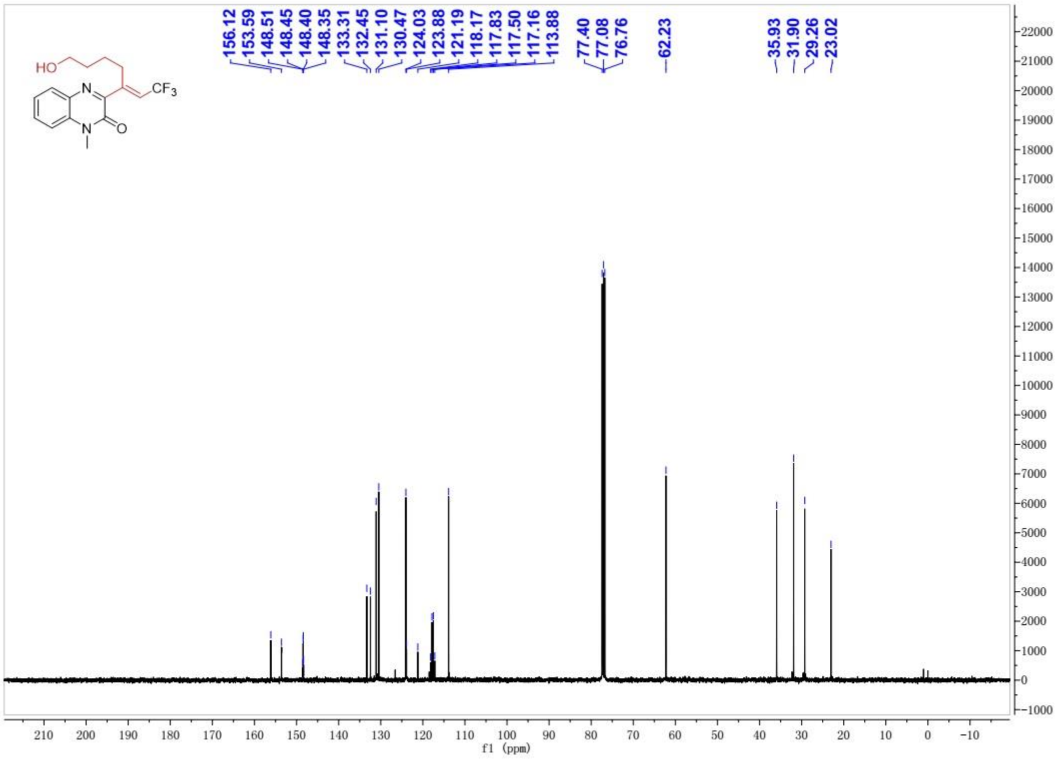

^{1}H and ^{13}C NMR Spectra for **3ai**

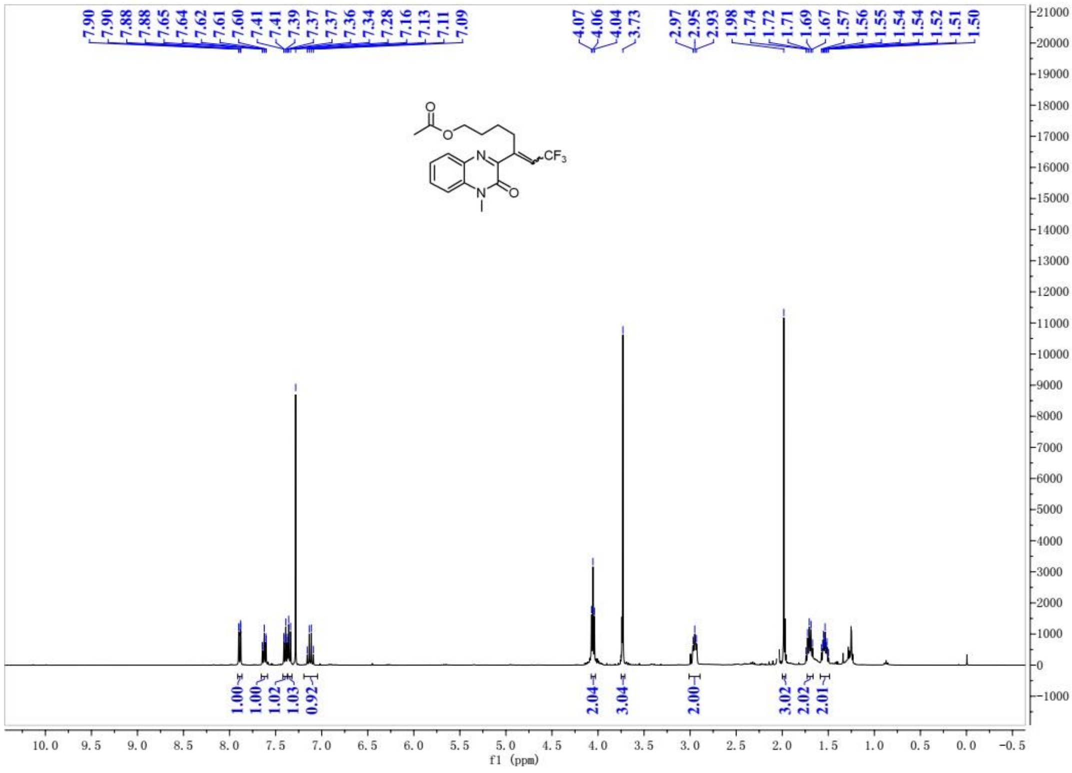

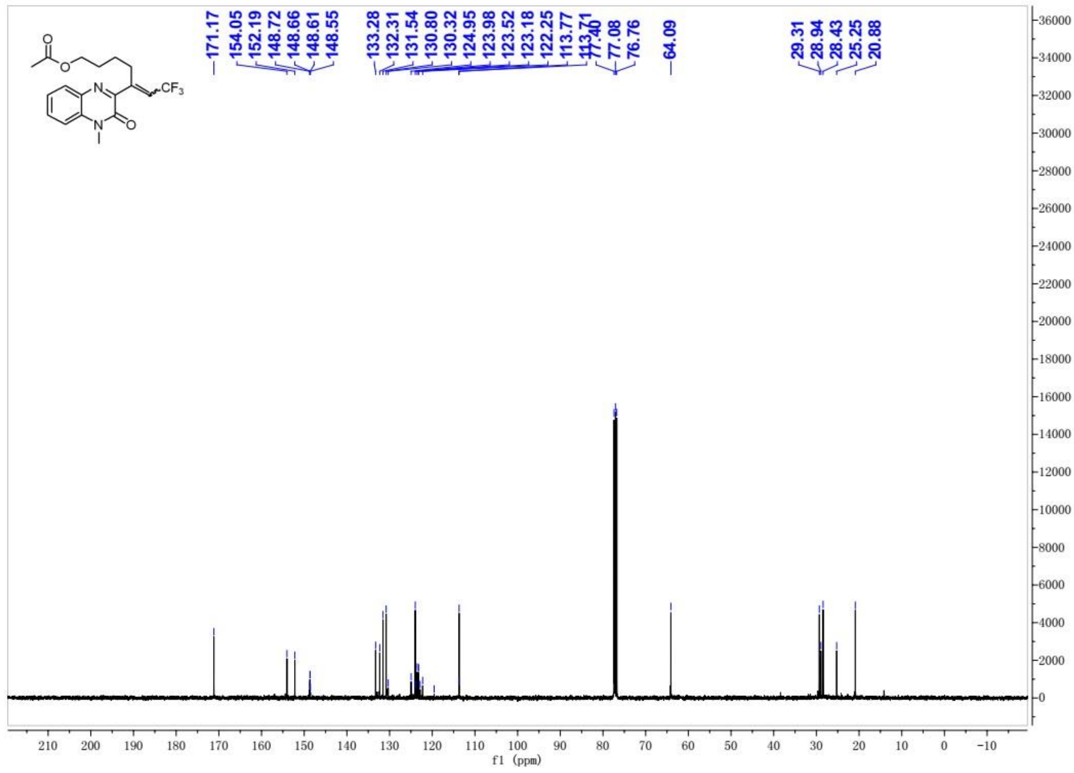

^{1}H and ^{13}C NMR Spectra for **3aj**

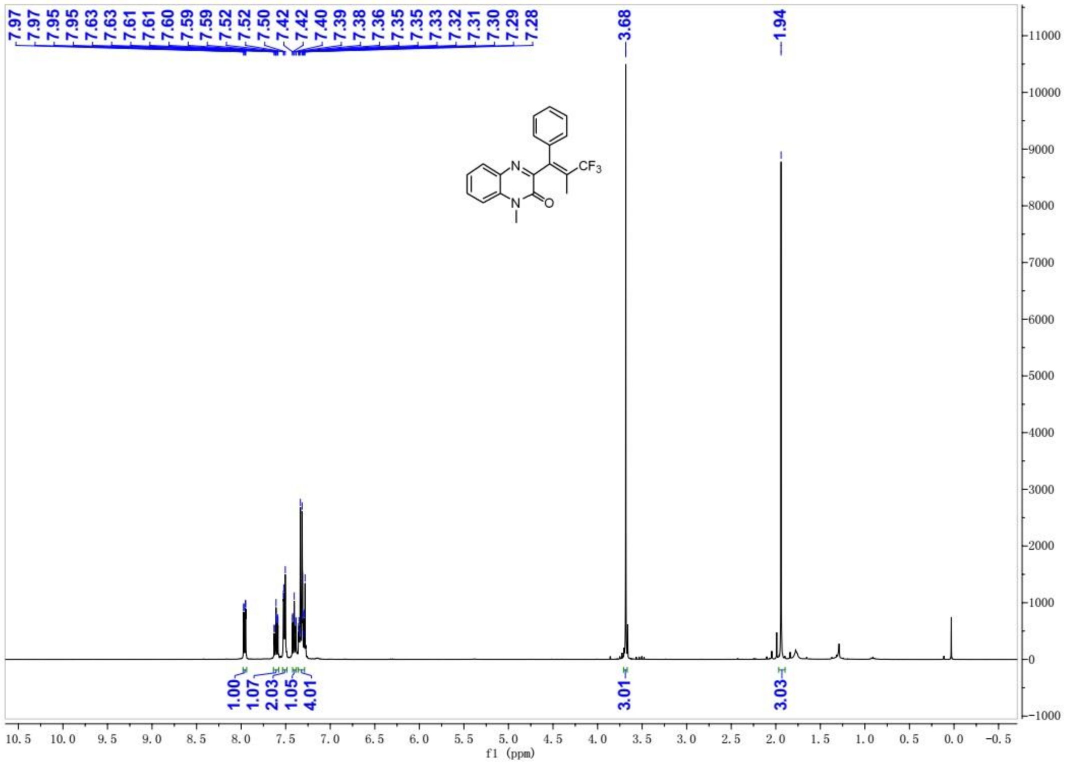

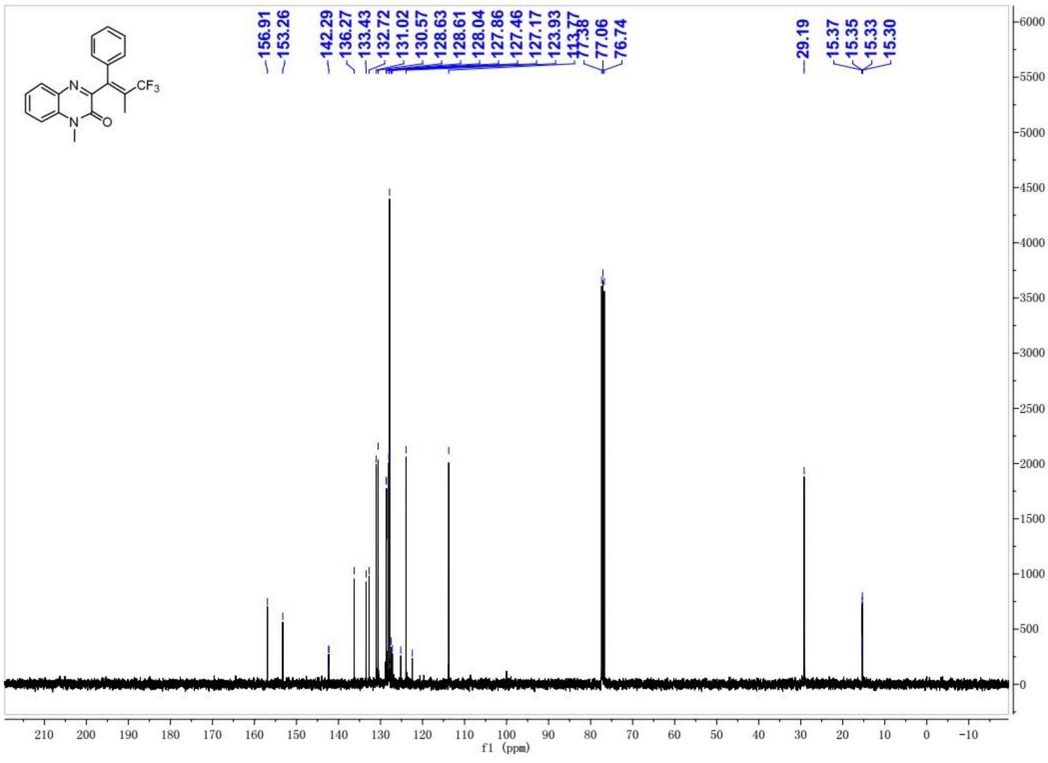

^{1}H and ^{13}C NMR Spectra for **3ak**

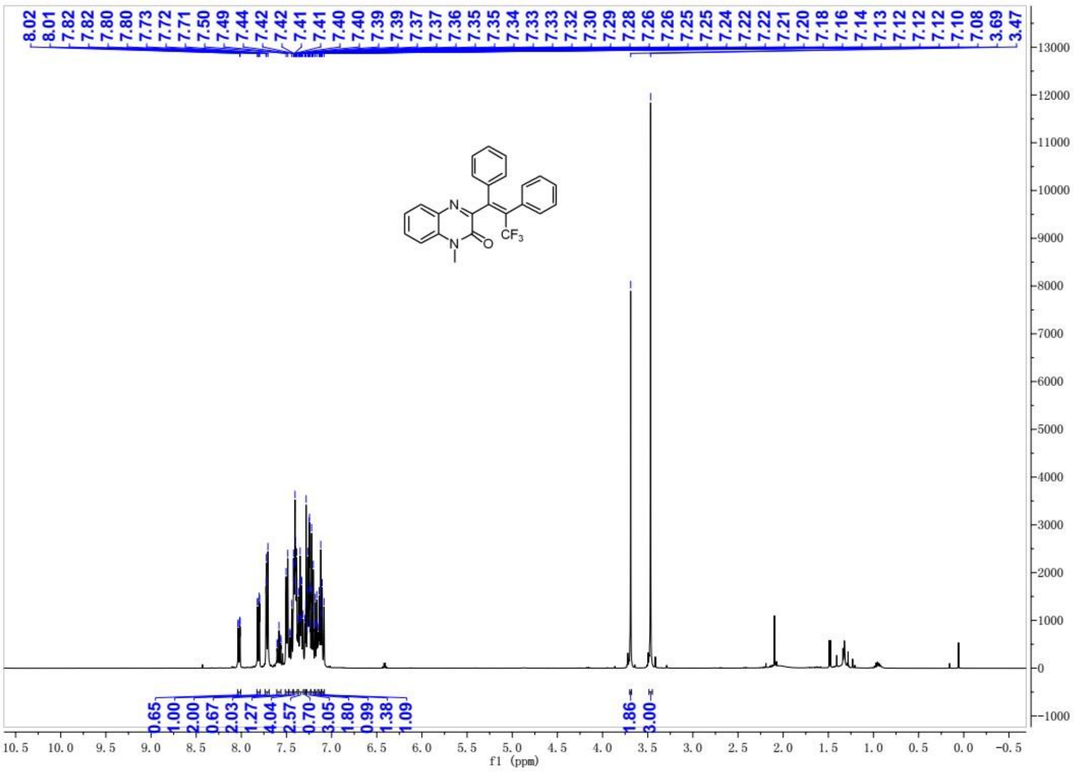

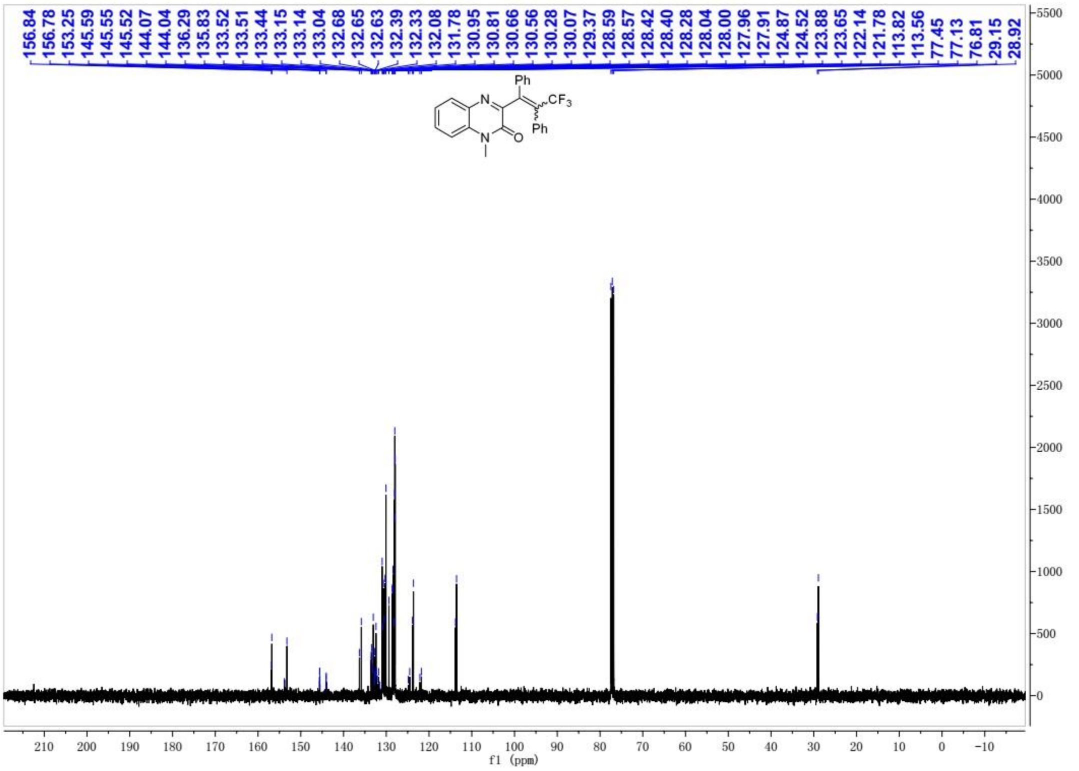

^{19}F NMR Spectra for **3a**

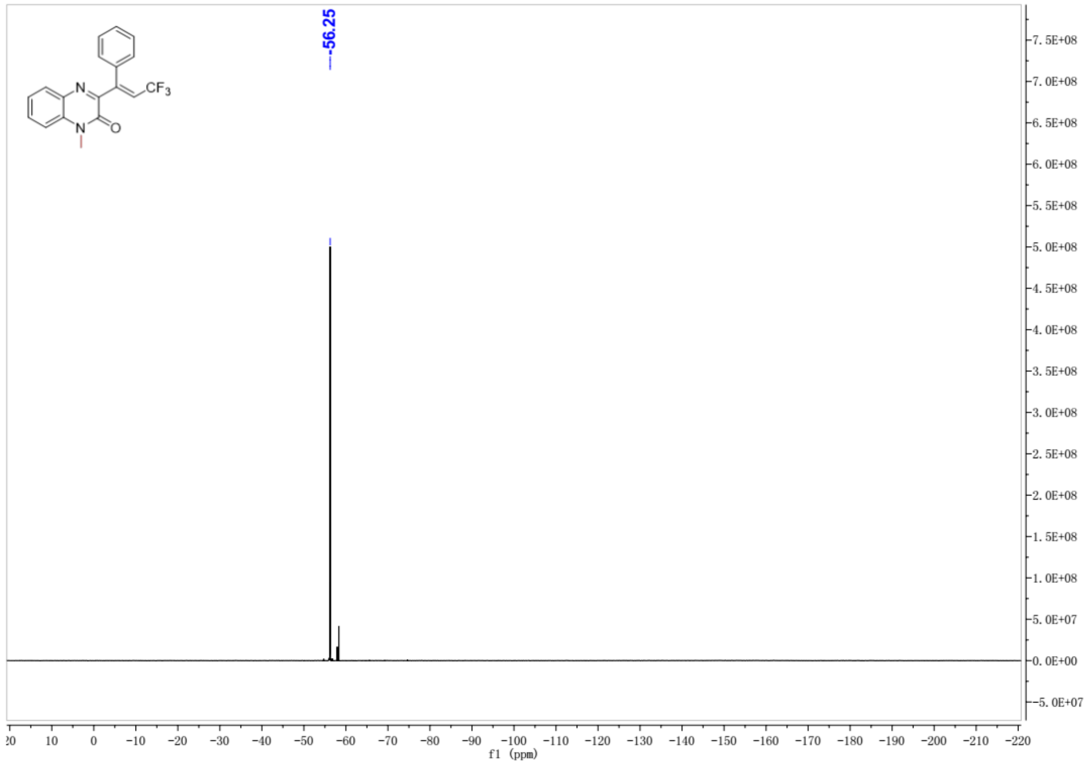

^{19}F NMR Spectra for **3b**

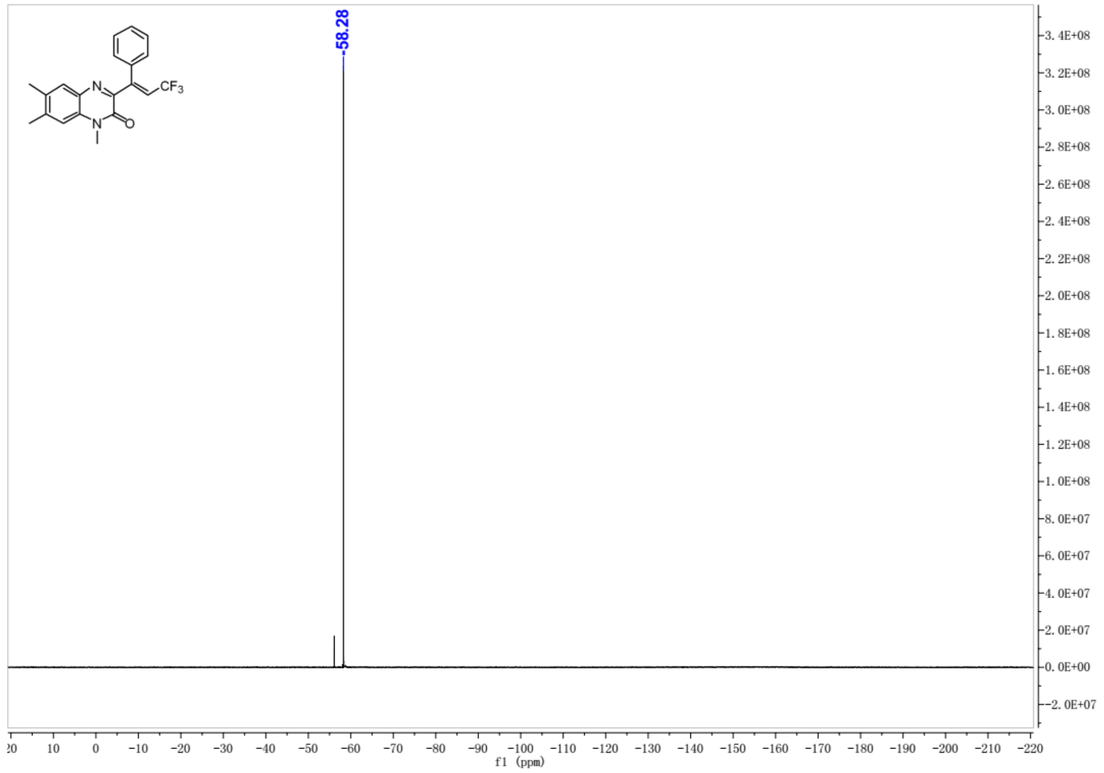

^{19}F NMR Spectra for **3c**

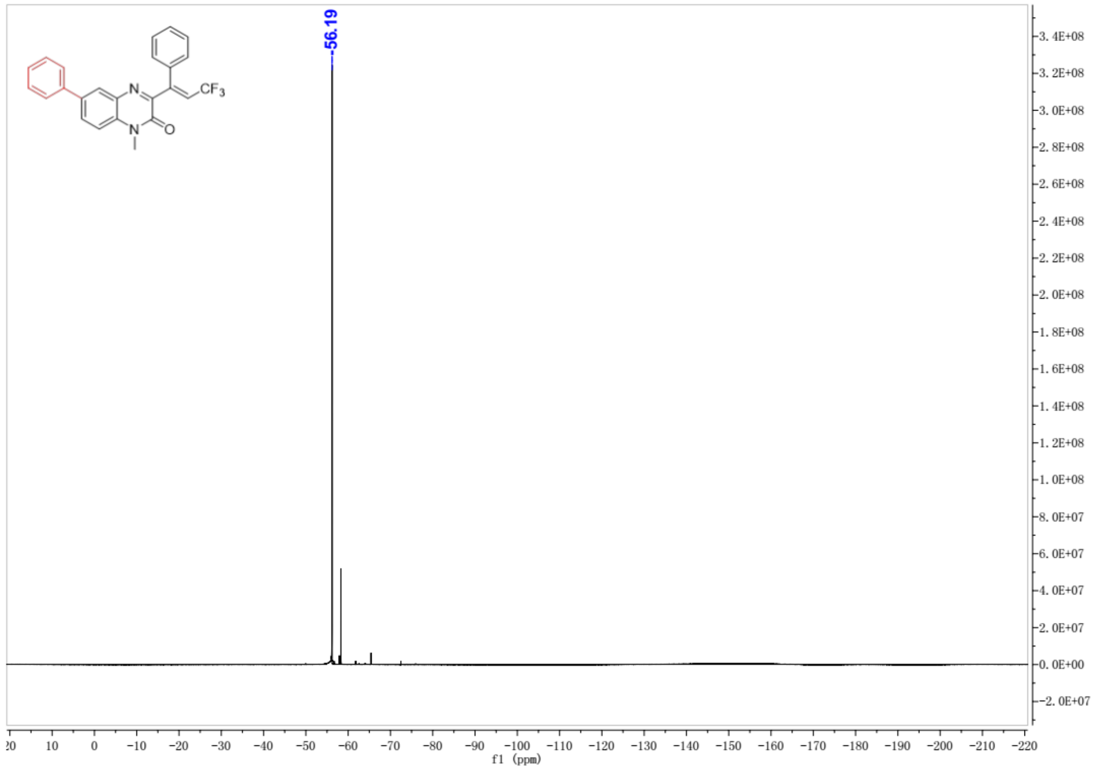

^{19}F NMR Spectra for **3d**

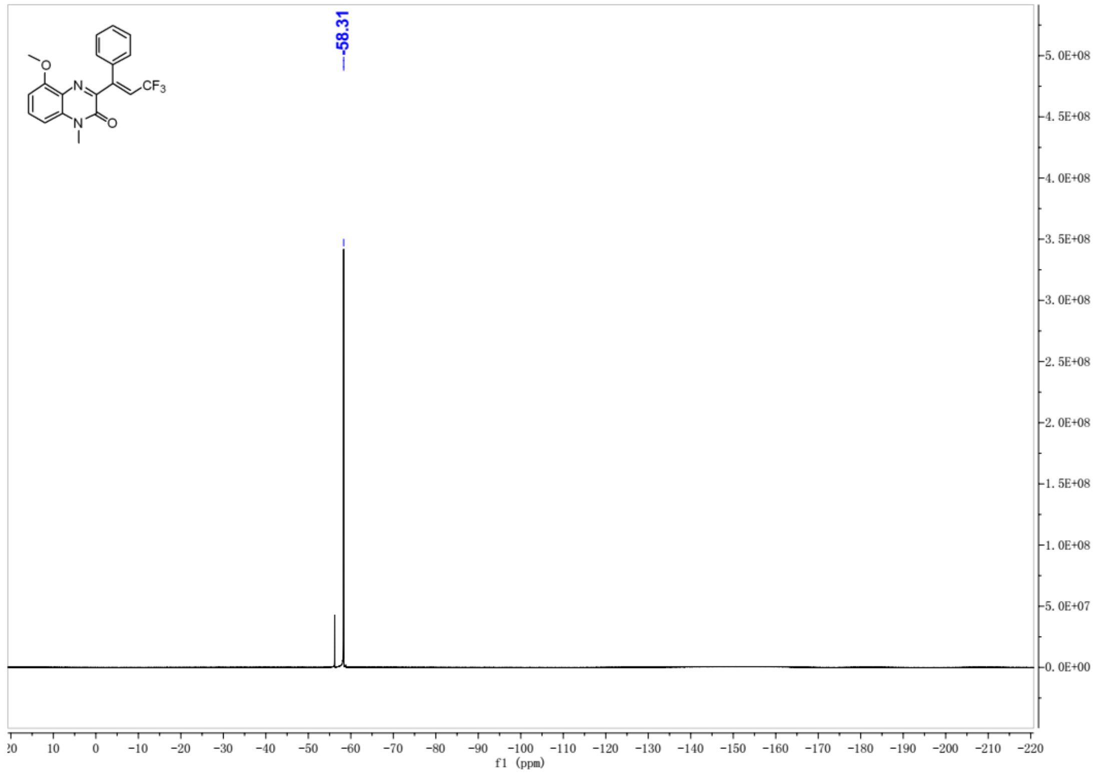

^{19}F NMR Spectra for **3e**

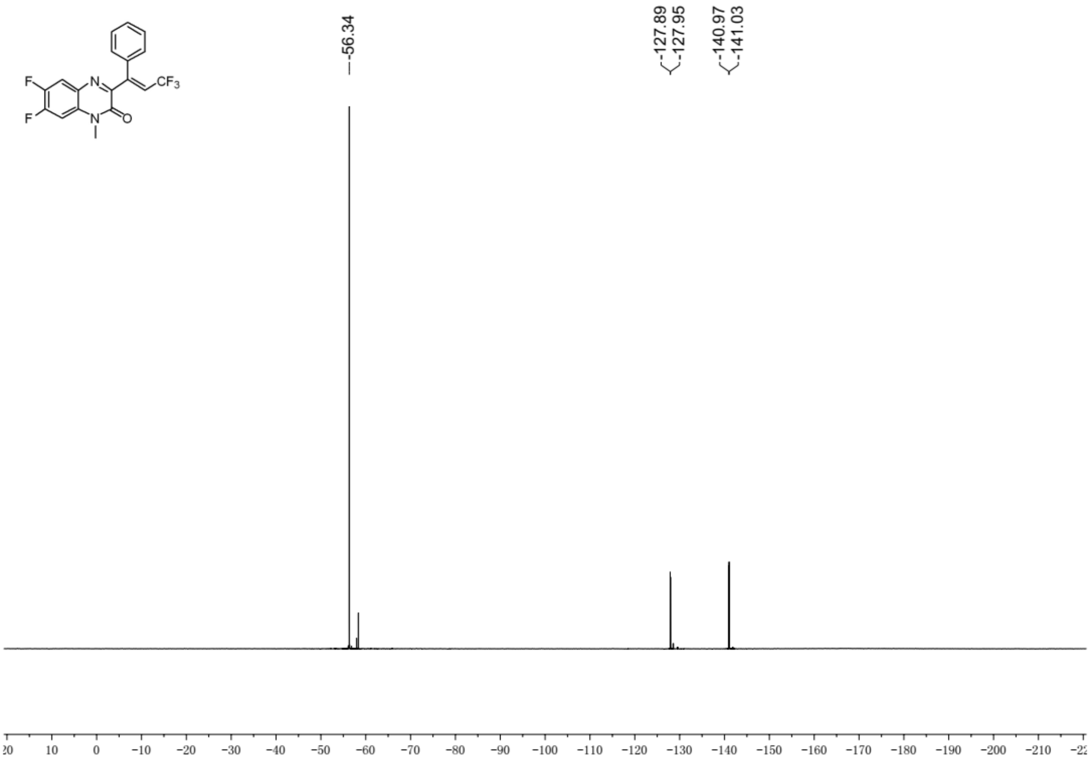

^{19}F NMR Spectra for **3f**

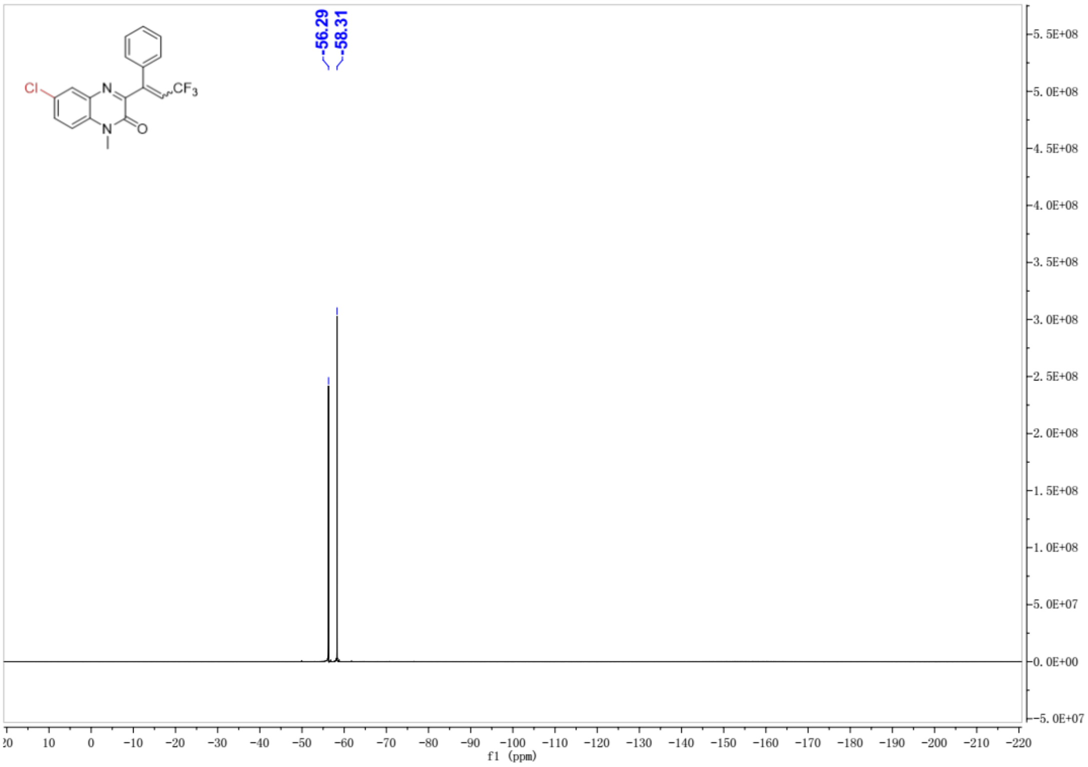

^{19}F NMR Spectra for **3g**

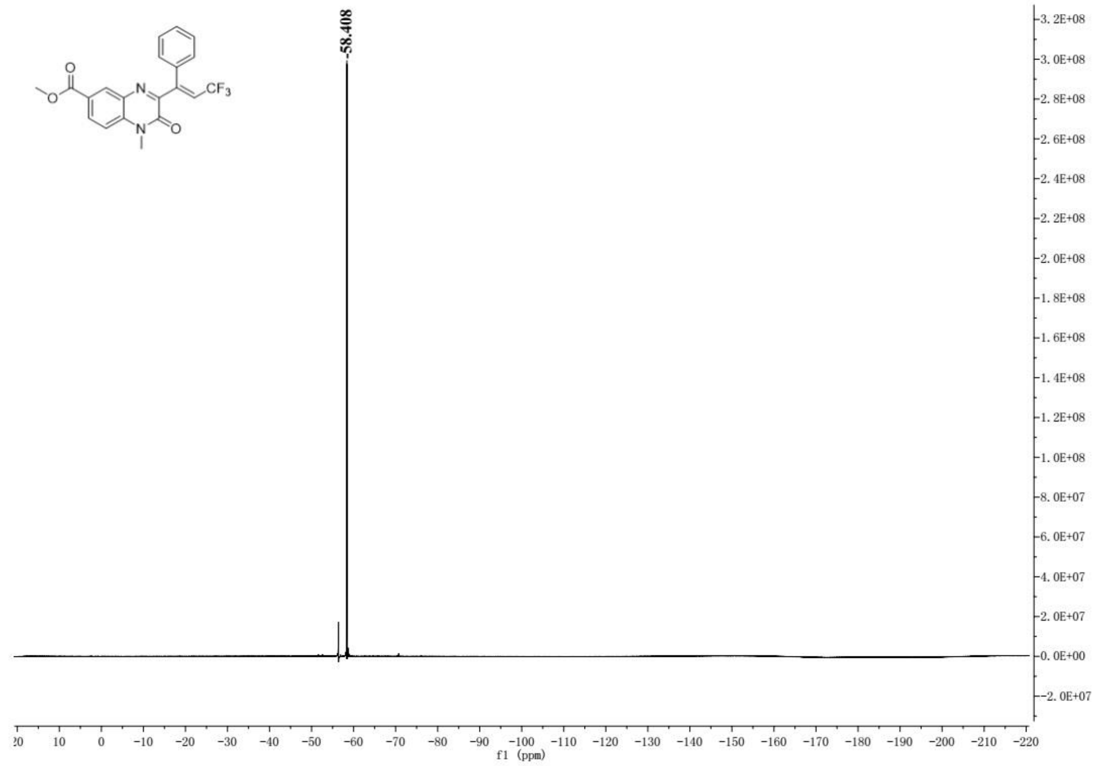

^{19}F NMR Spectra for **3h**

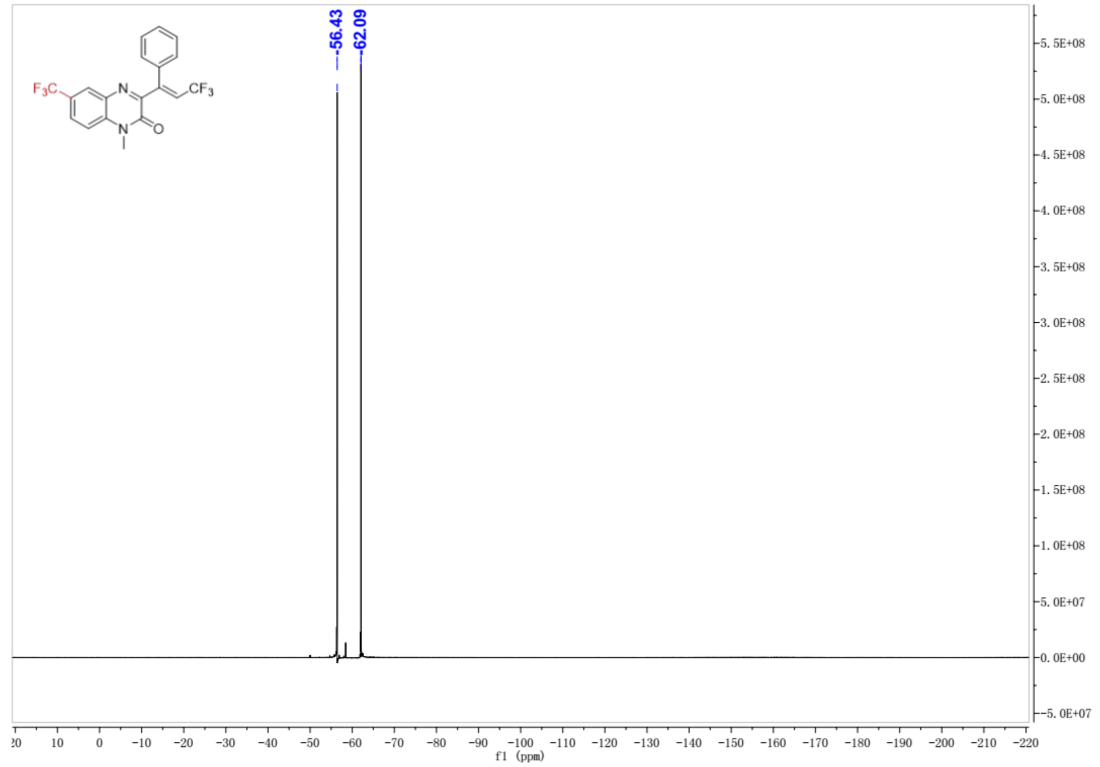

^{19}F NMR Spectra for **3i**

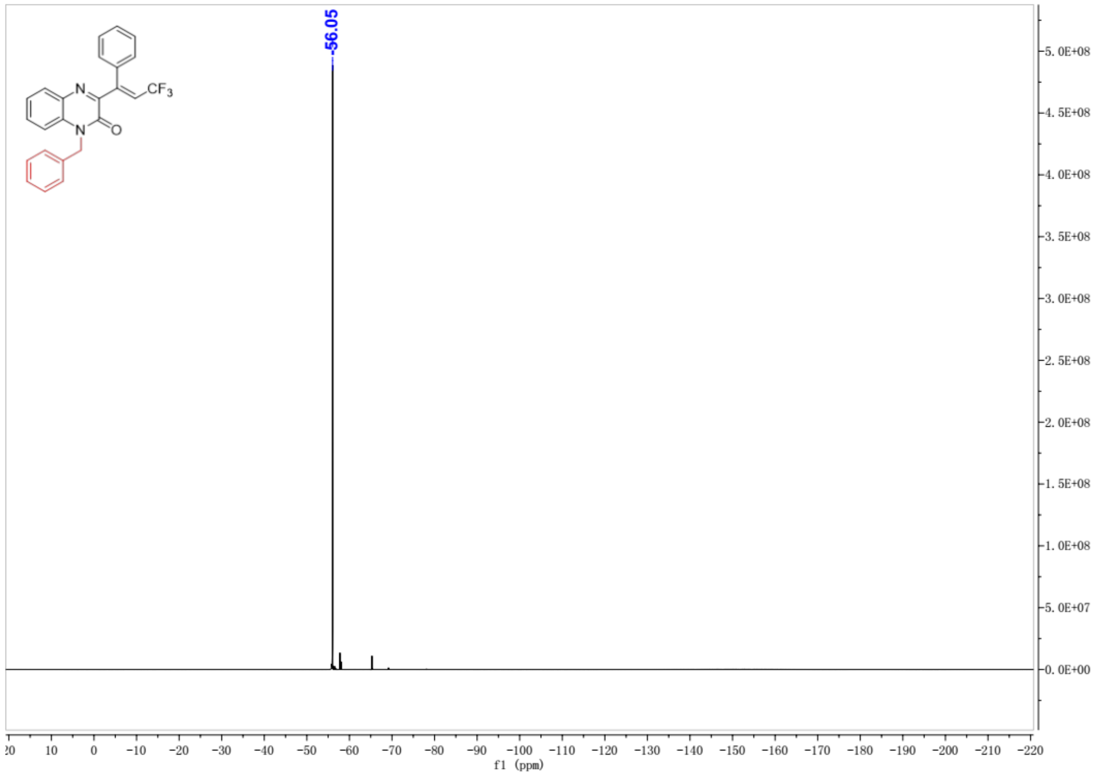

^{19}F NMR Spectra for **3j**

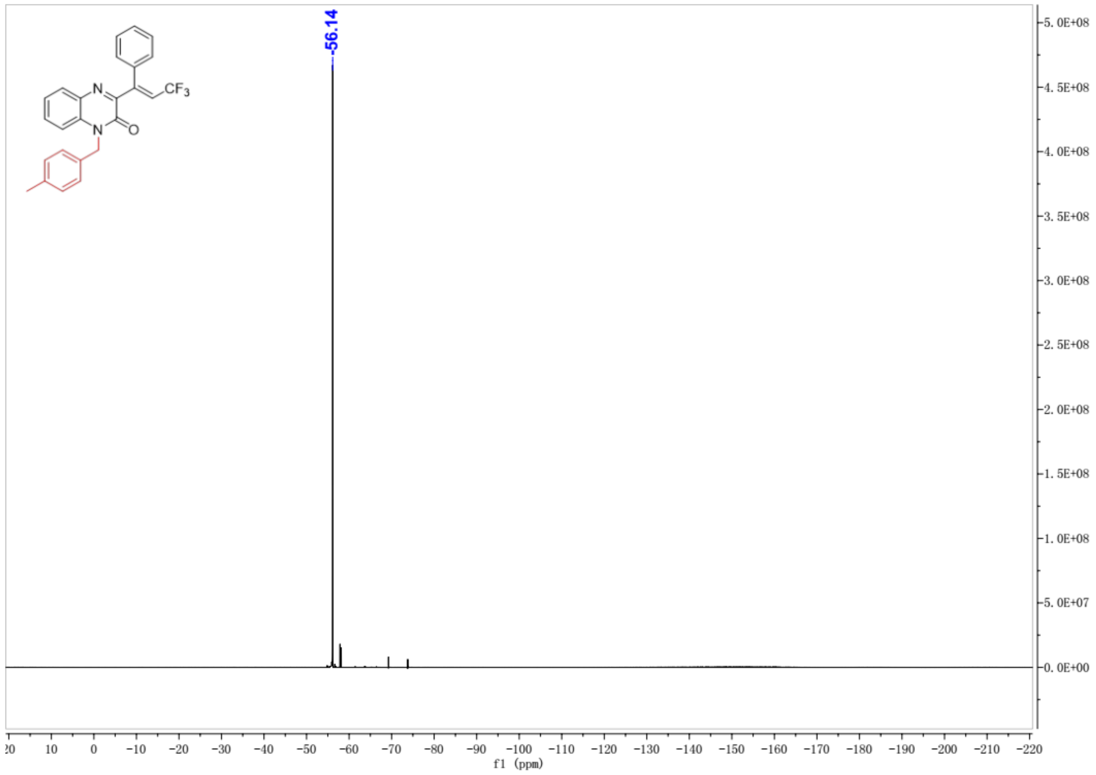

^{19}F NMR Spectra for **3k**

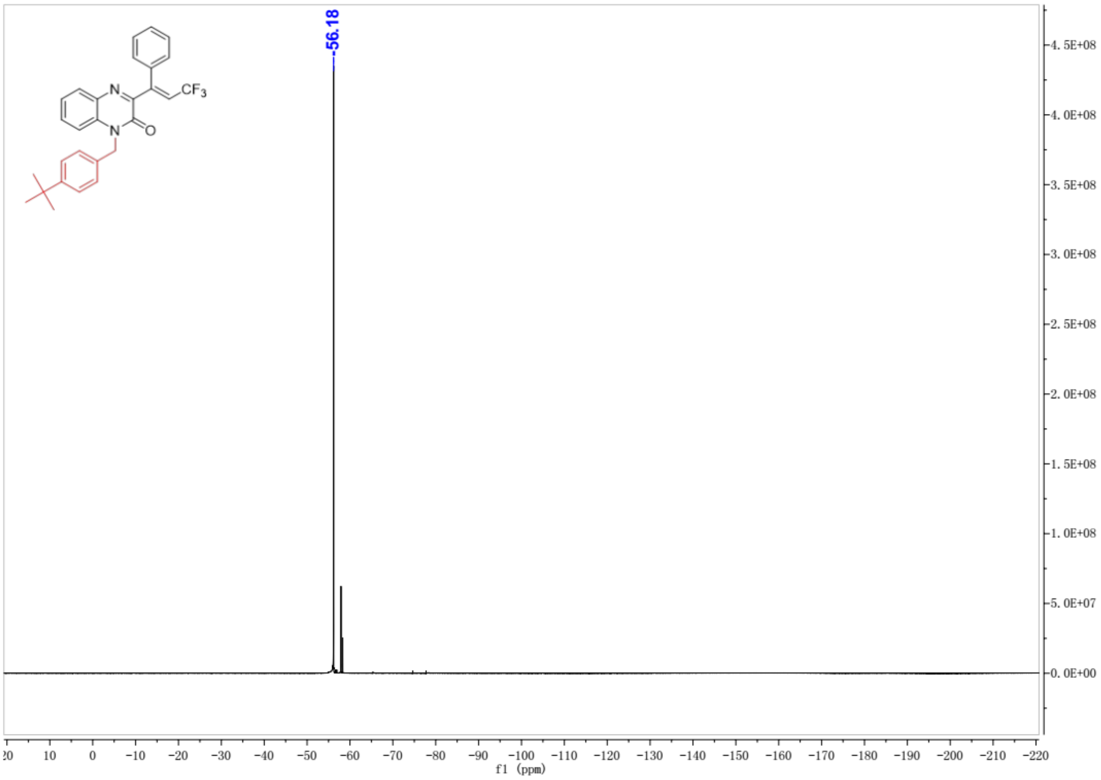

^{19}F NMR Spectra for **3l**

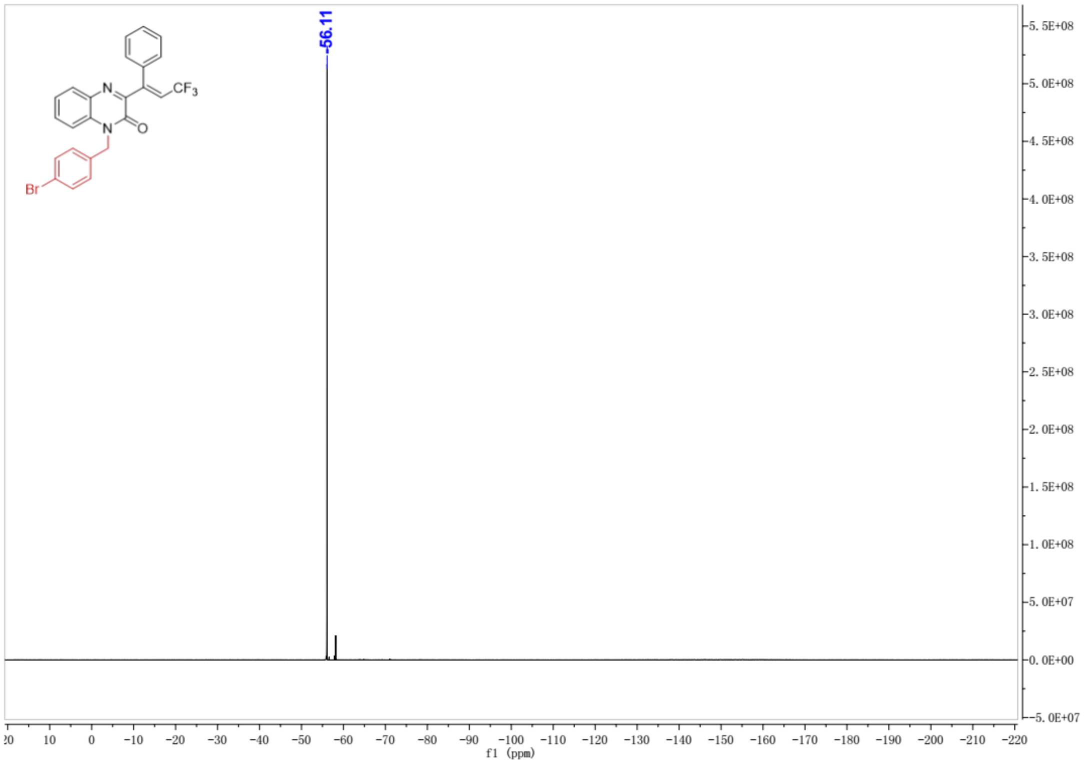

^{19}F NMR Spectra for **3m**

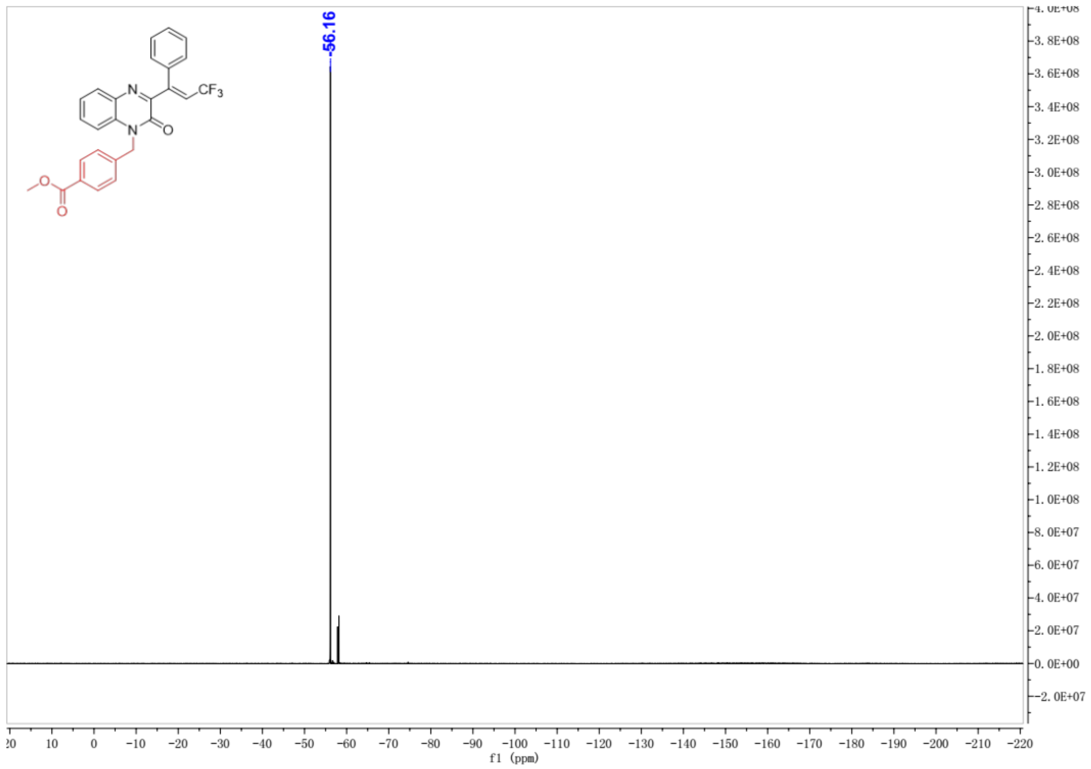

^{19}F NMR Spectra for **3n**

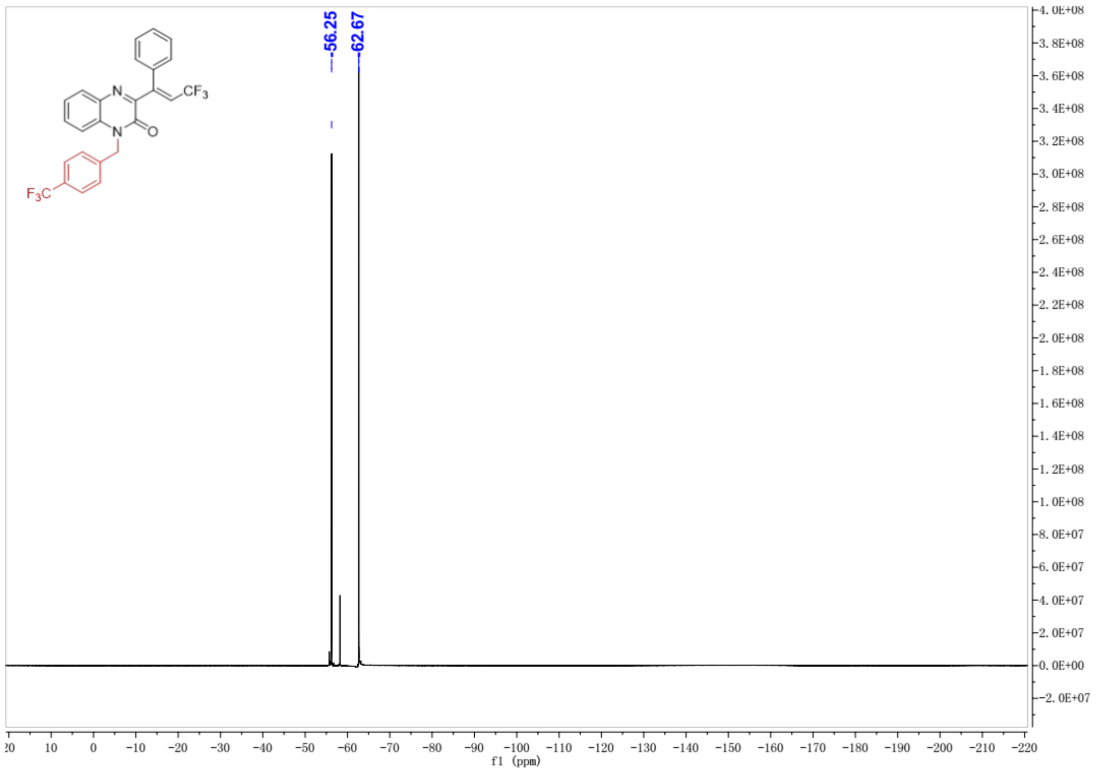

^{19}F NMR Spectra for **3o**

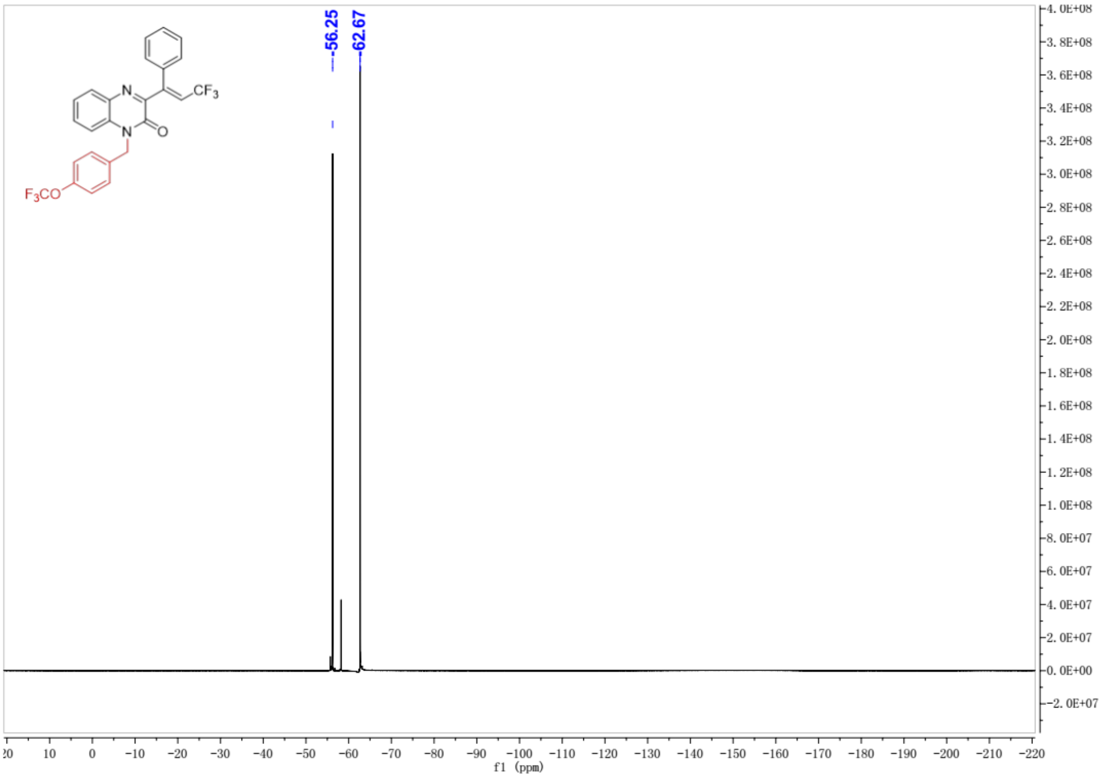

^{19}F NMR Spectra for **3p**

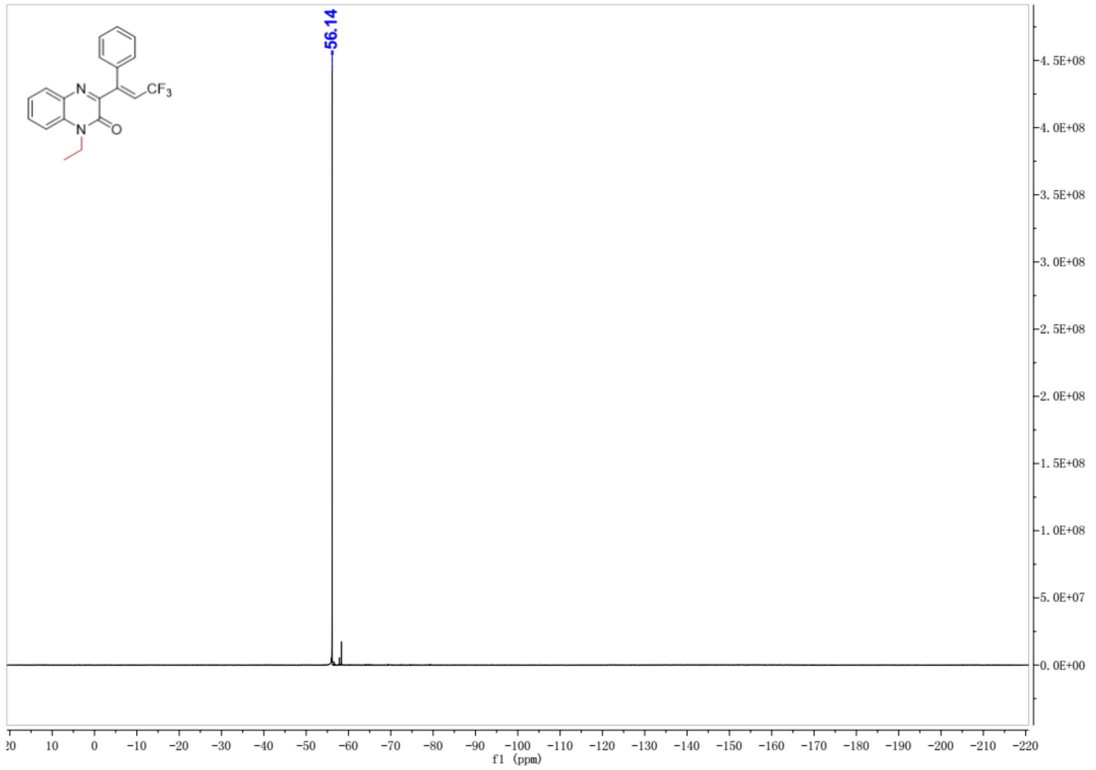

^{19}F NMR Spectra for **3q**

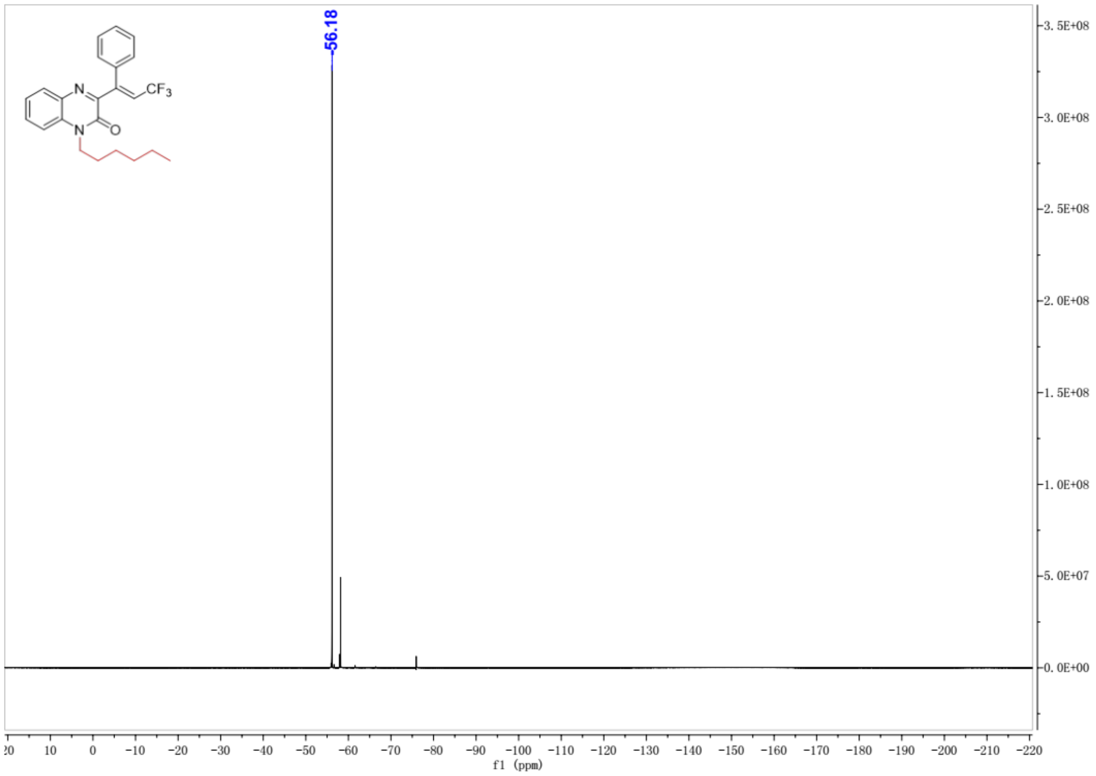

^{19}F NMR Spectra for **3r**

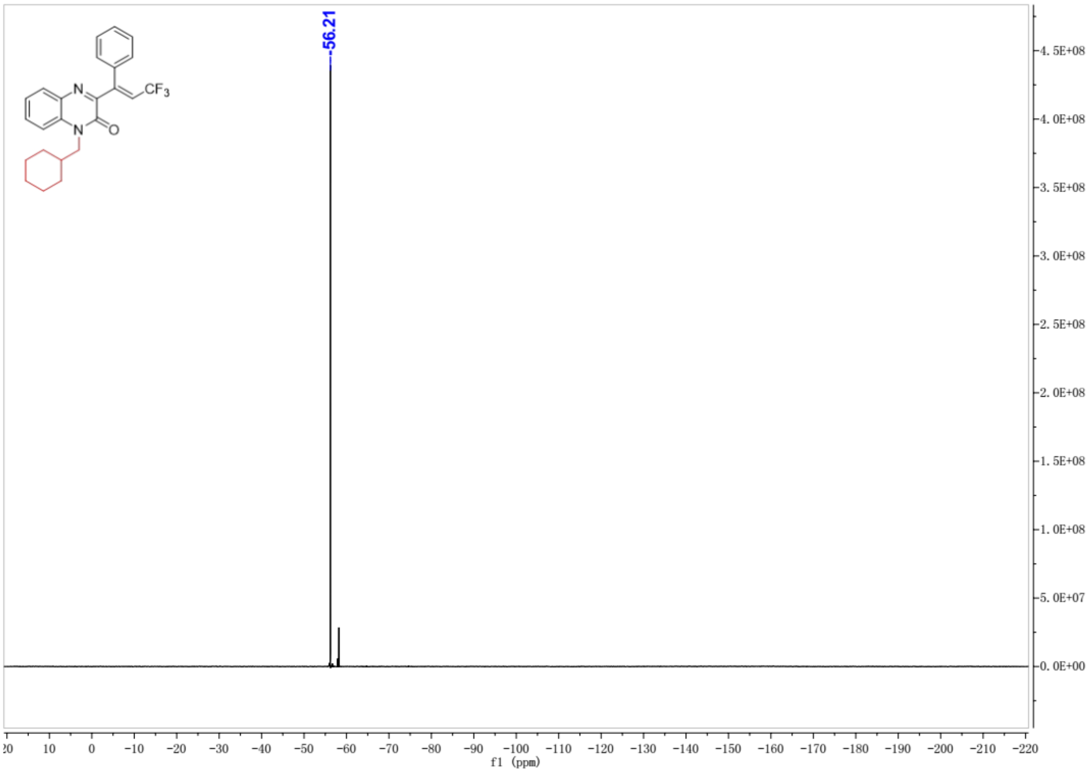

^{19}F NMR Spectra for **3s**

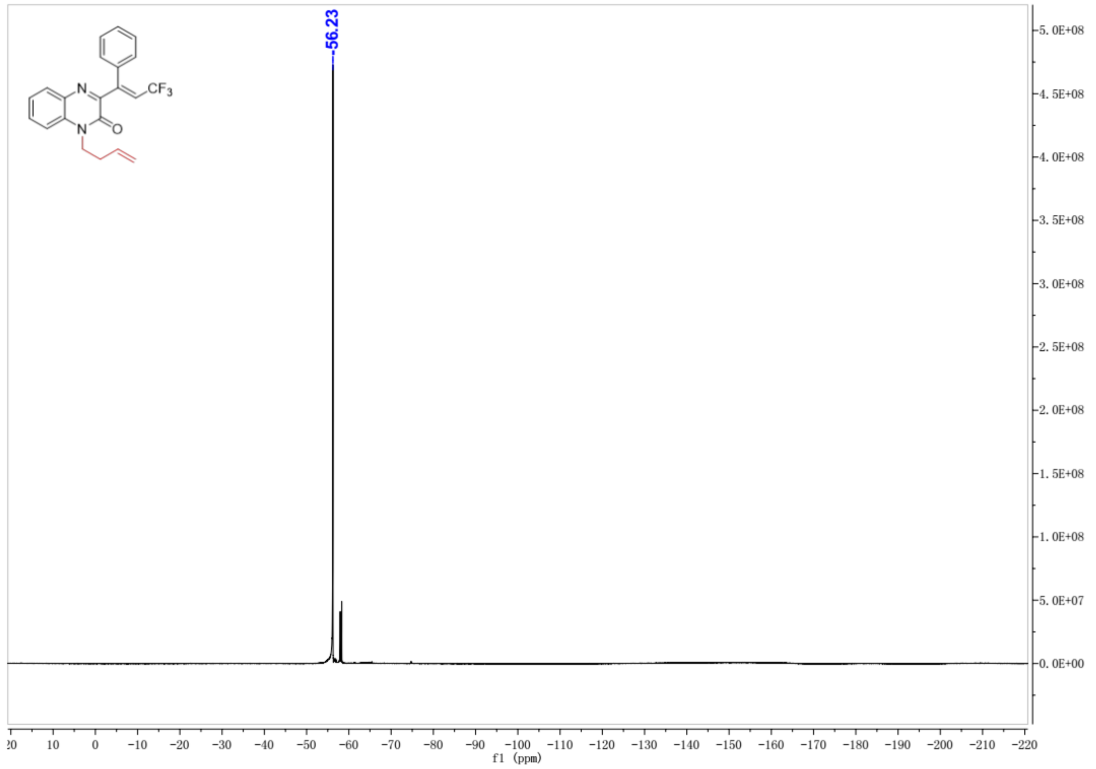

^{19}F NMR Spectra for **3t**

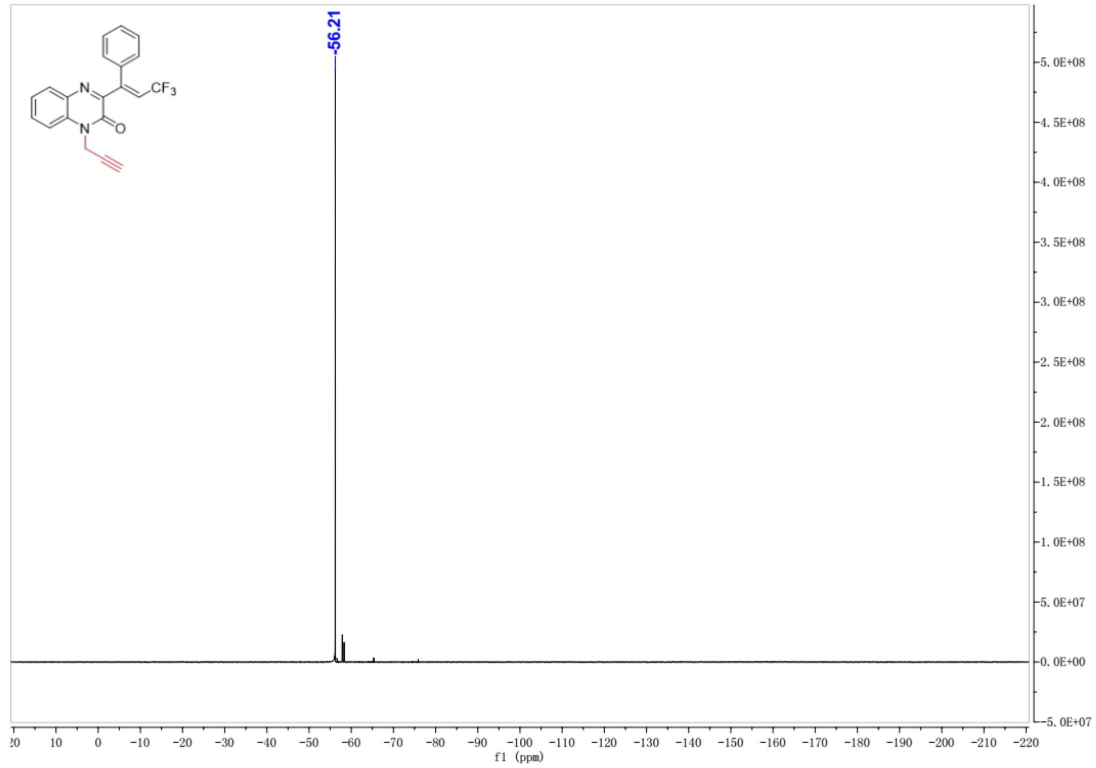

^{19}F NMR Spectra for **3u**

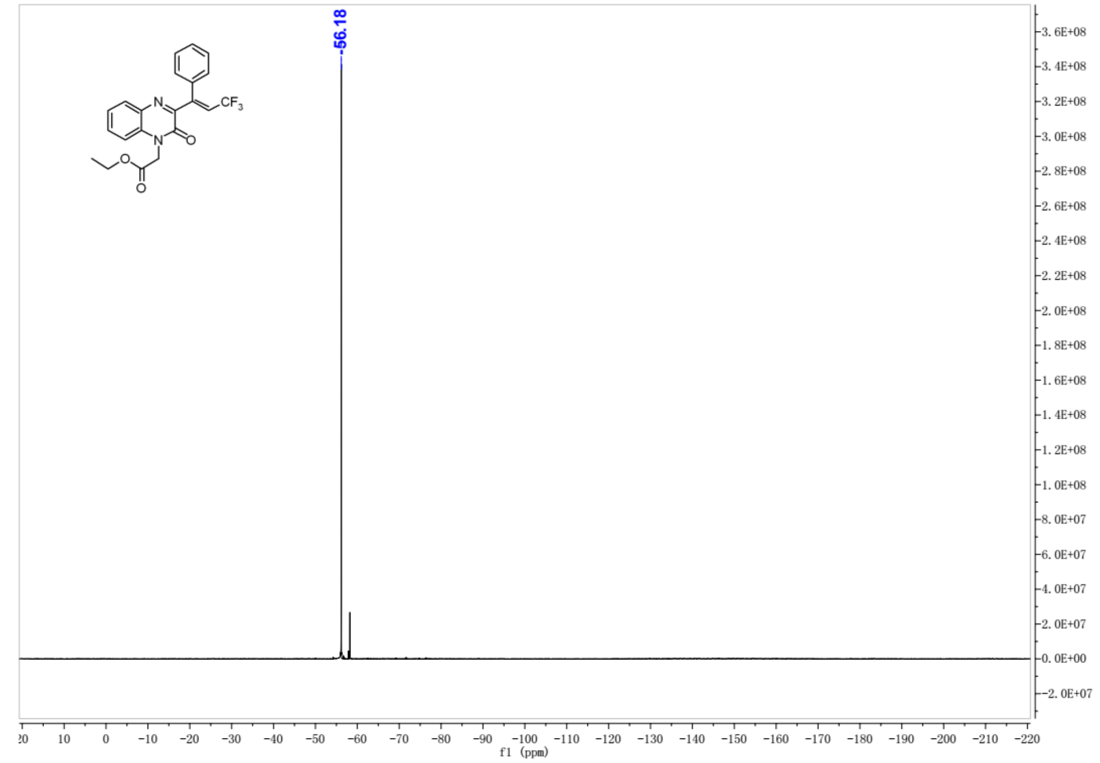

^{19}F NMR Spectra for **3v**

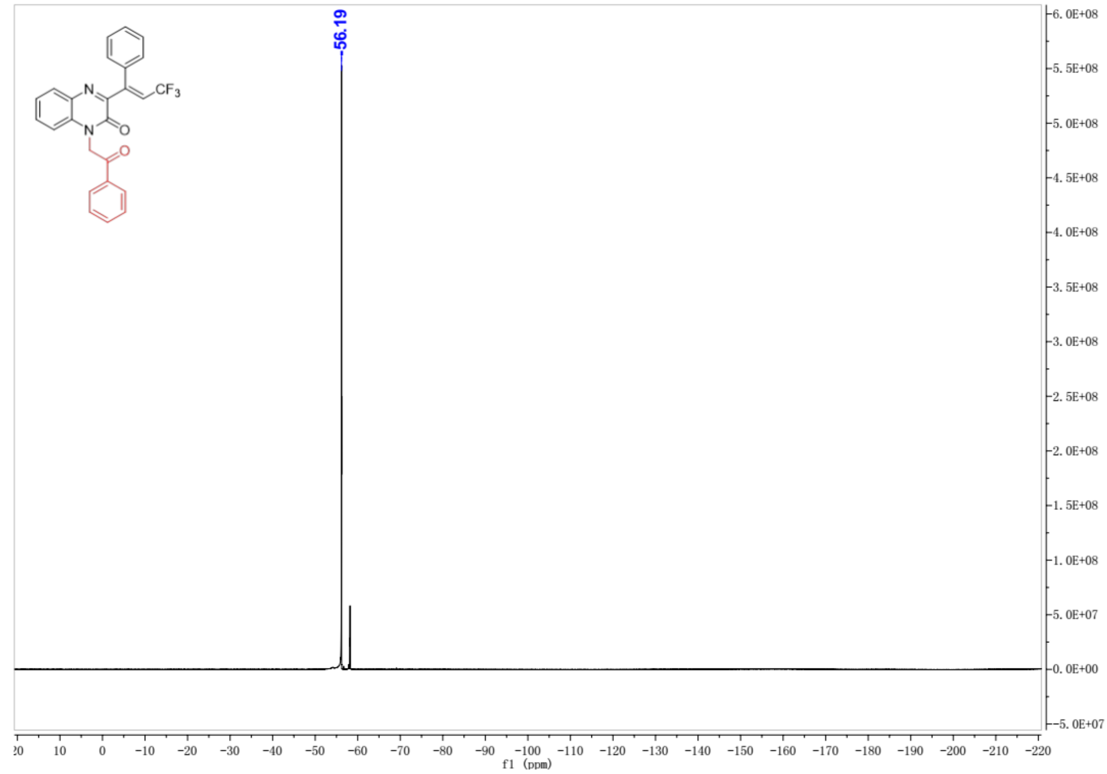

^{19}F NMR Spectra for **3w**

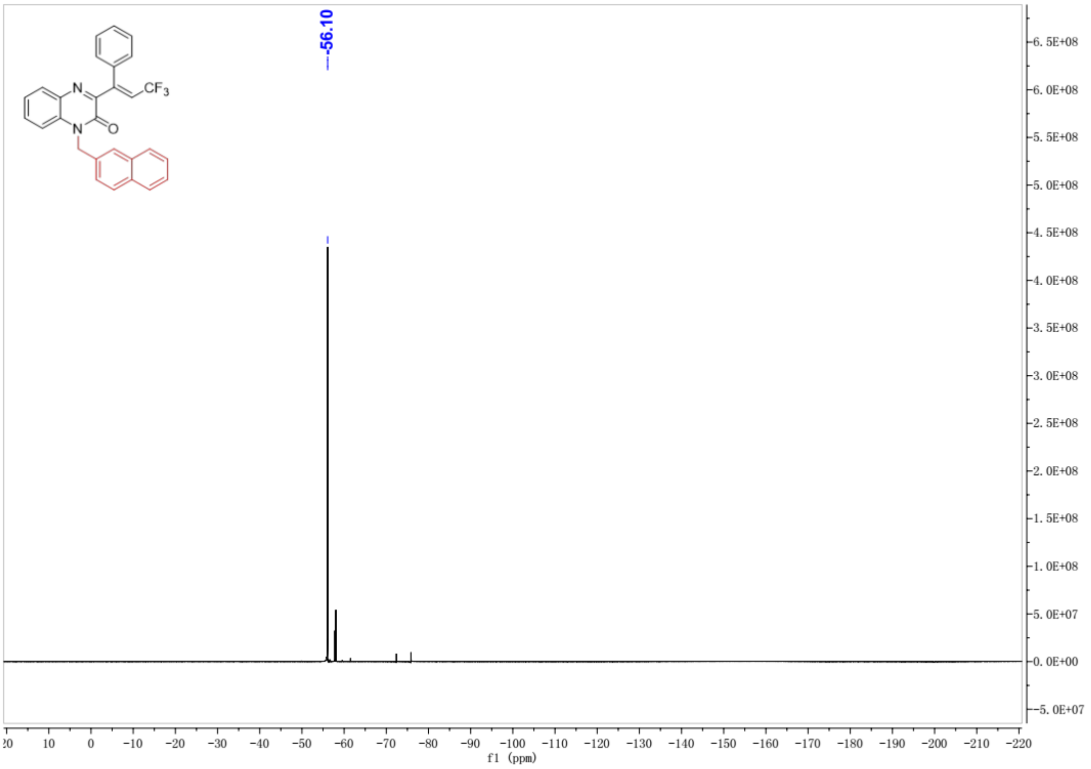

^{19}F NMR Spectra for **3x**

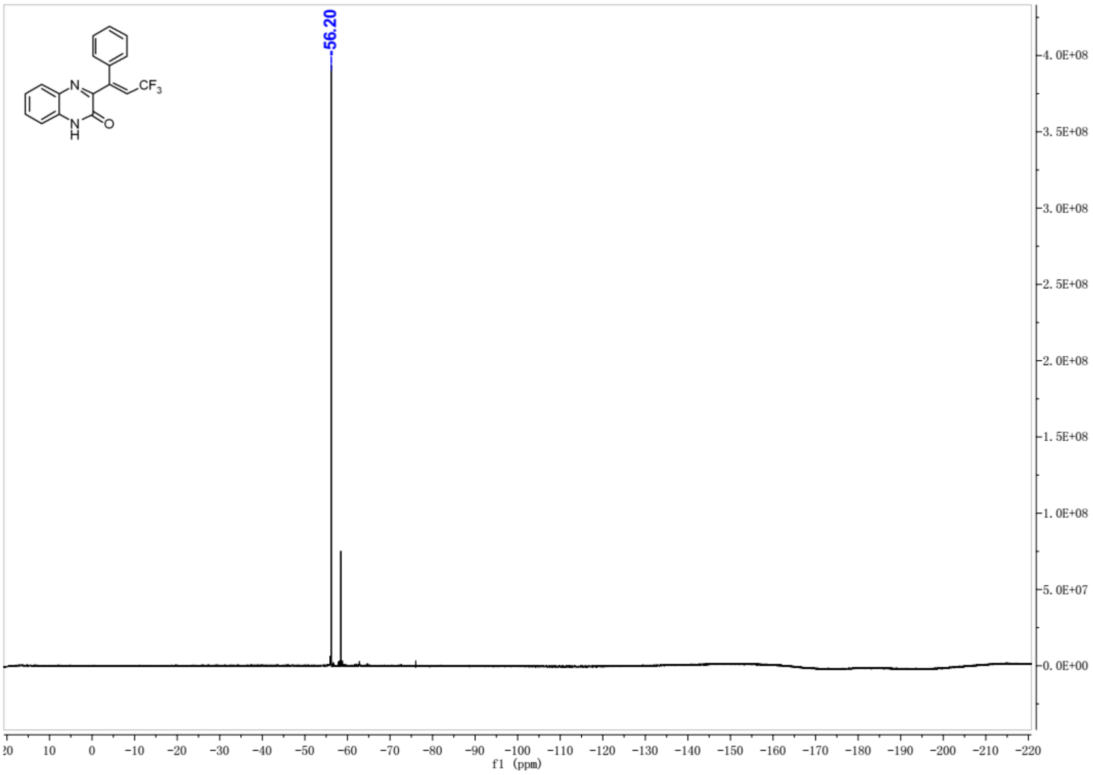

^{19}F NMR Spectra for **3y**

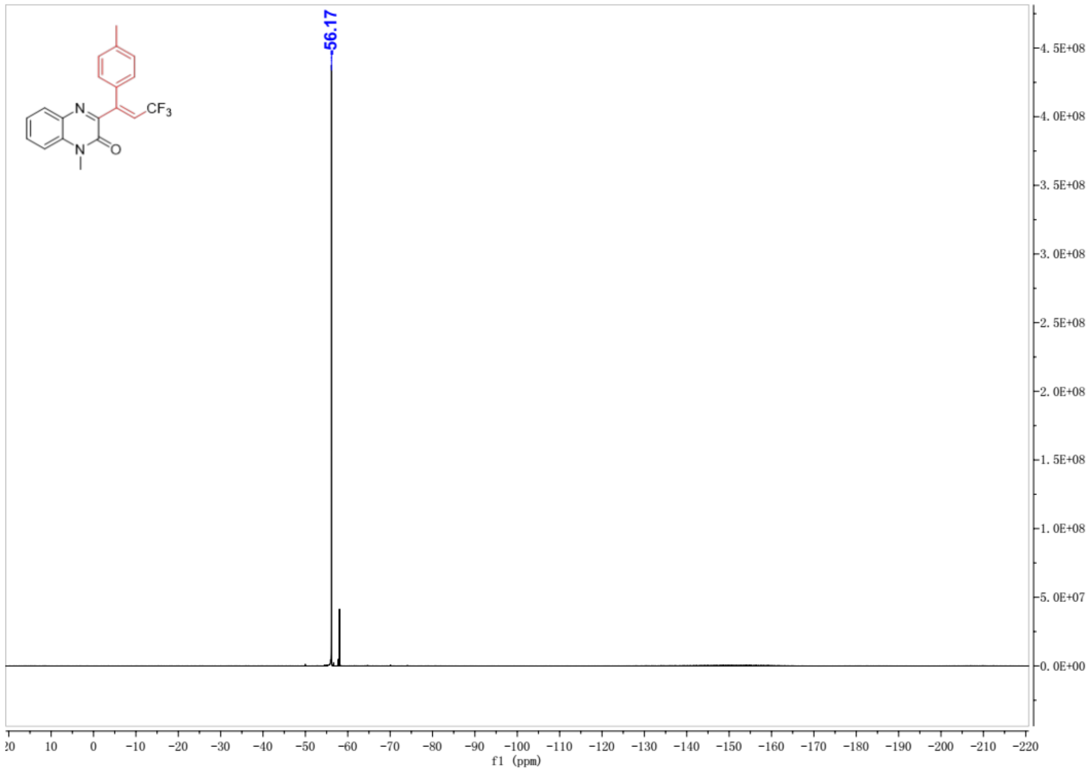

^{19}F NMR Spectra for **3z**

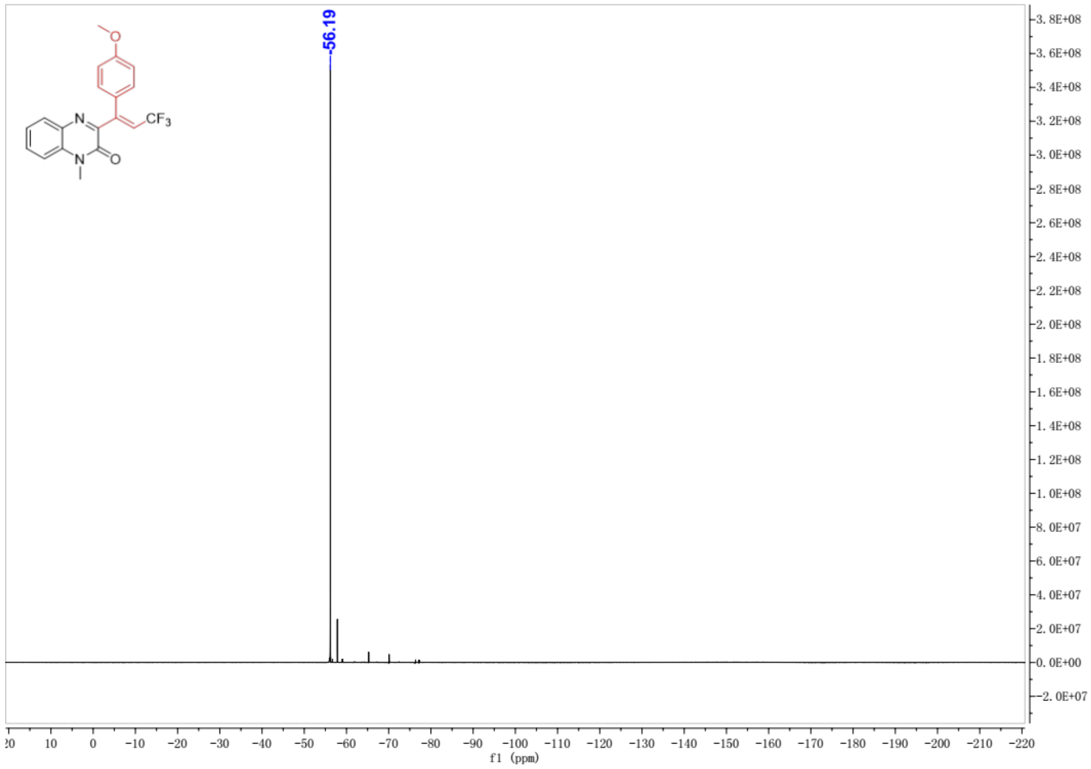

^{19}F NMR Spectra for **3aa**

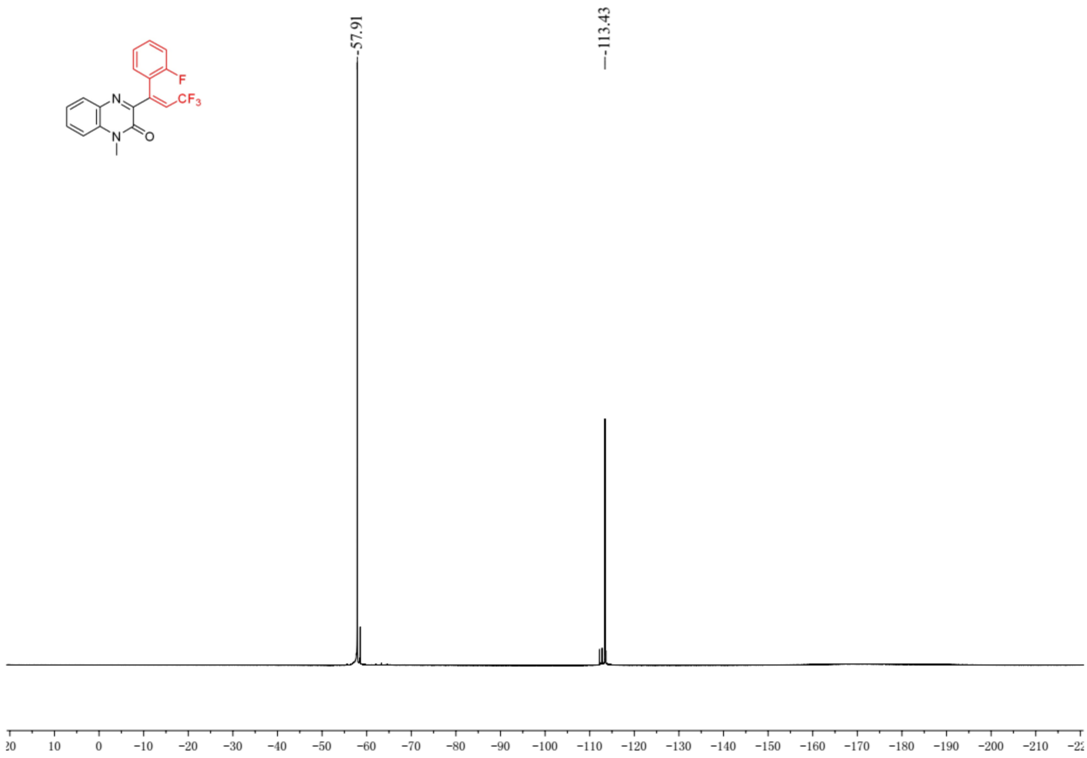

^{19}F NMR Spectra for **3ab**

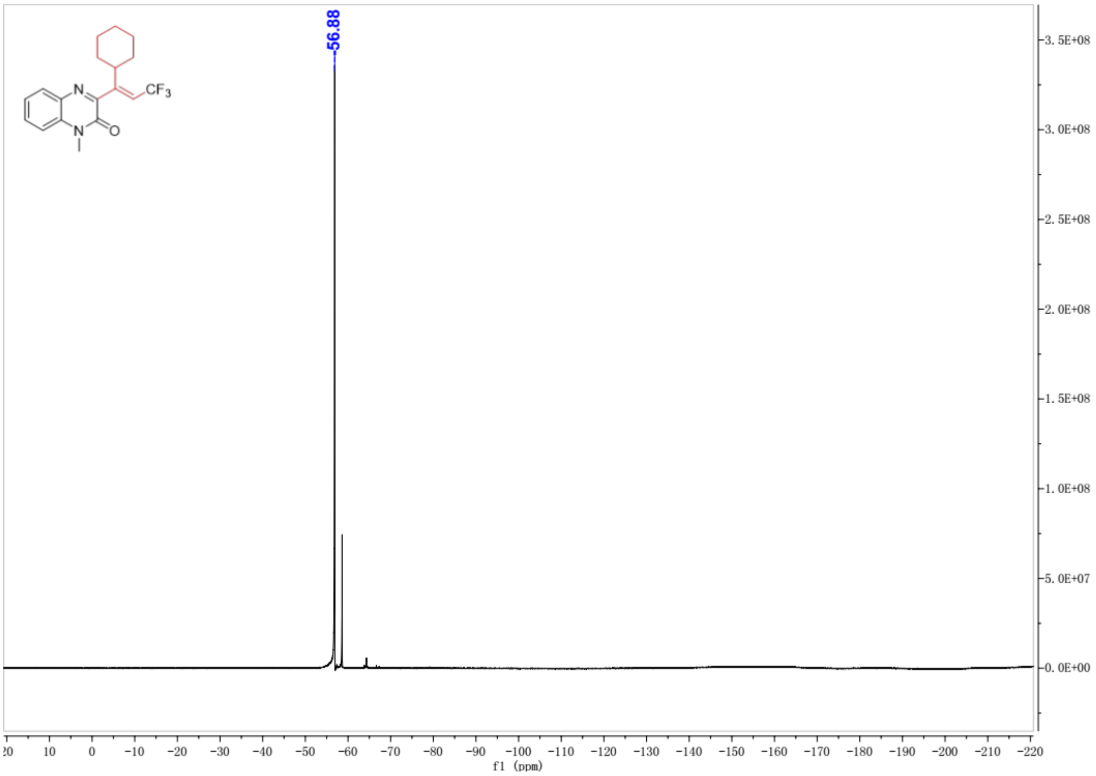

^{19}F NMR Spectra for **3ac**

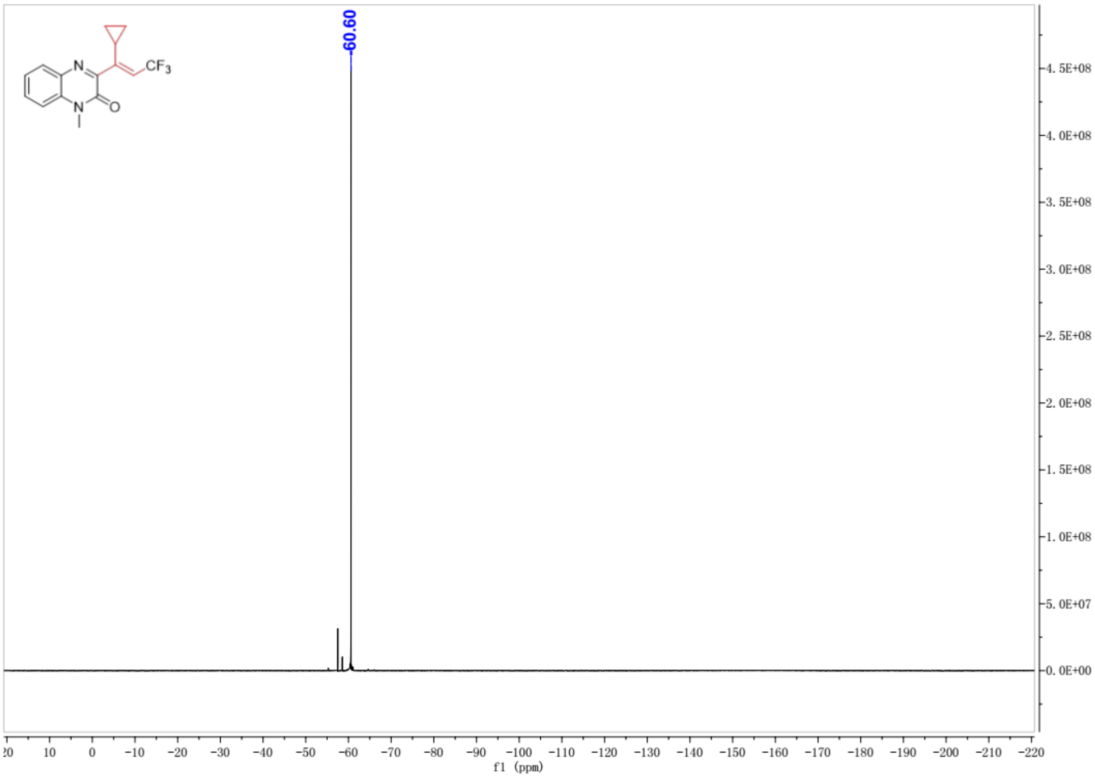

^{19}F NMR Spectra for **3ad**

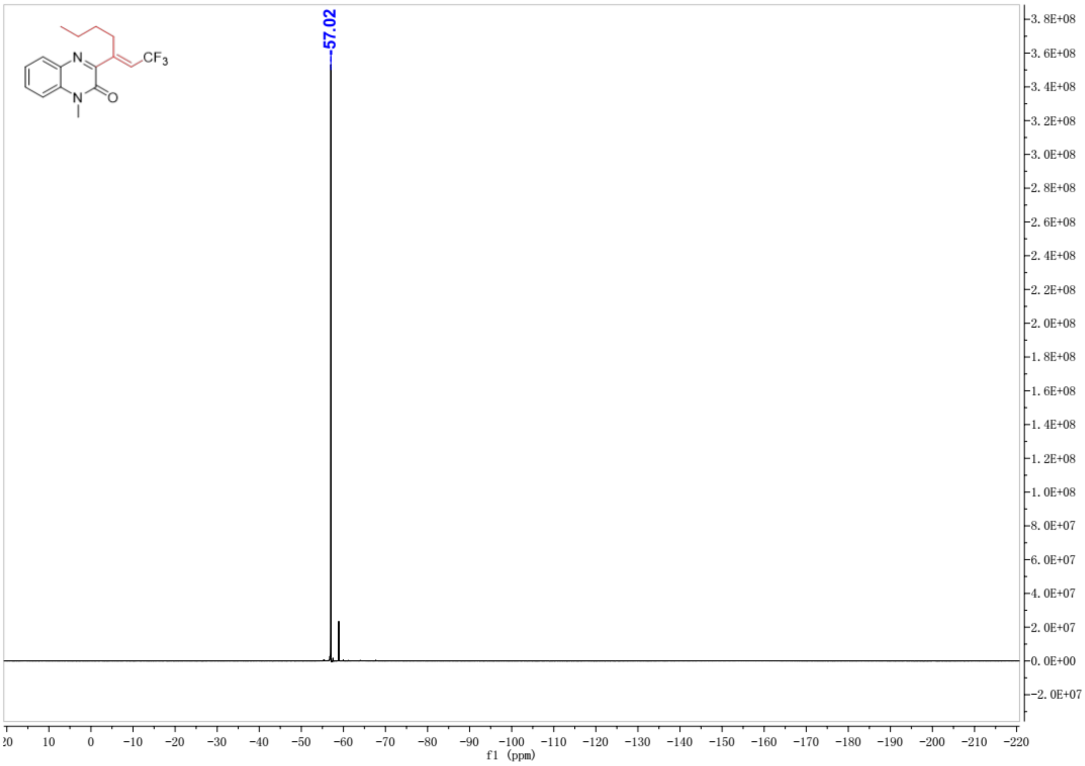

^{19}F NMR Spectra for **3ae**

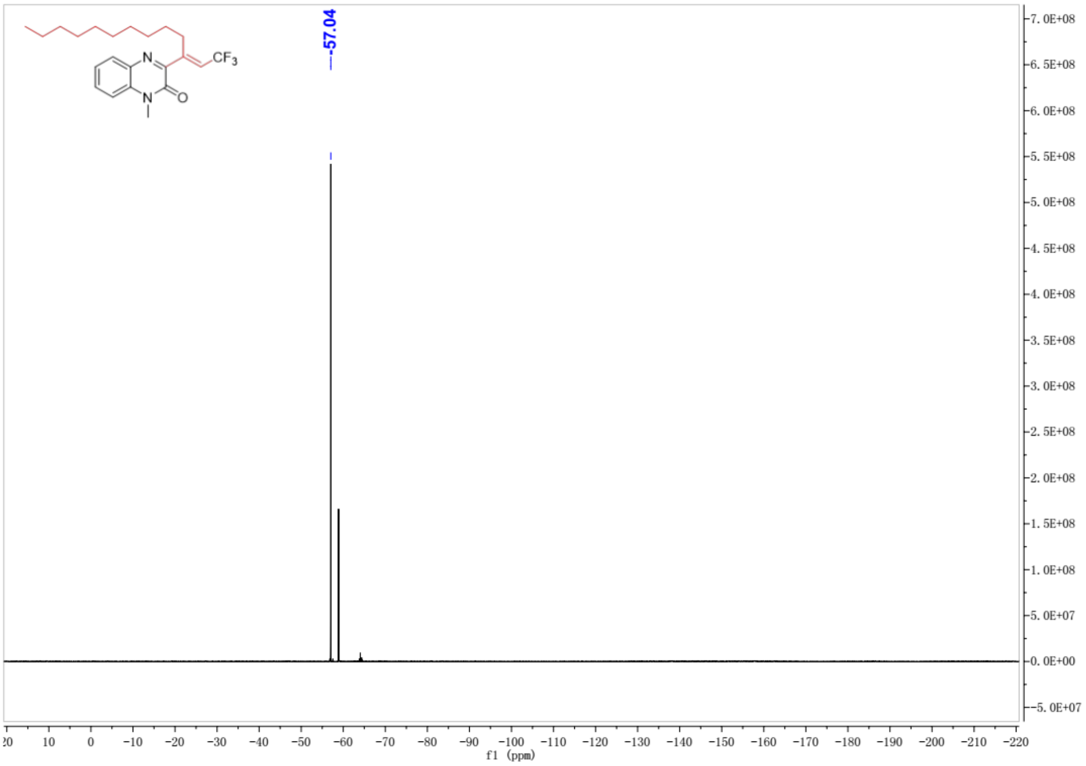

^{19}F NMR Spectra for **3af**

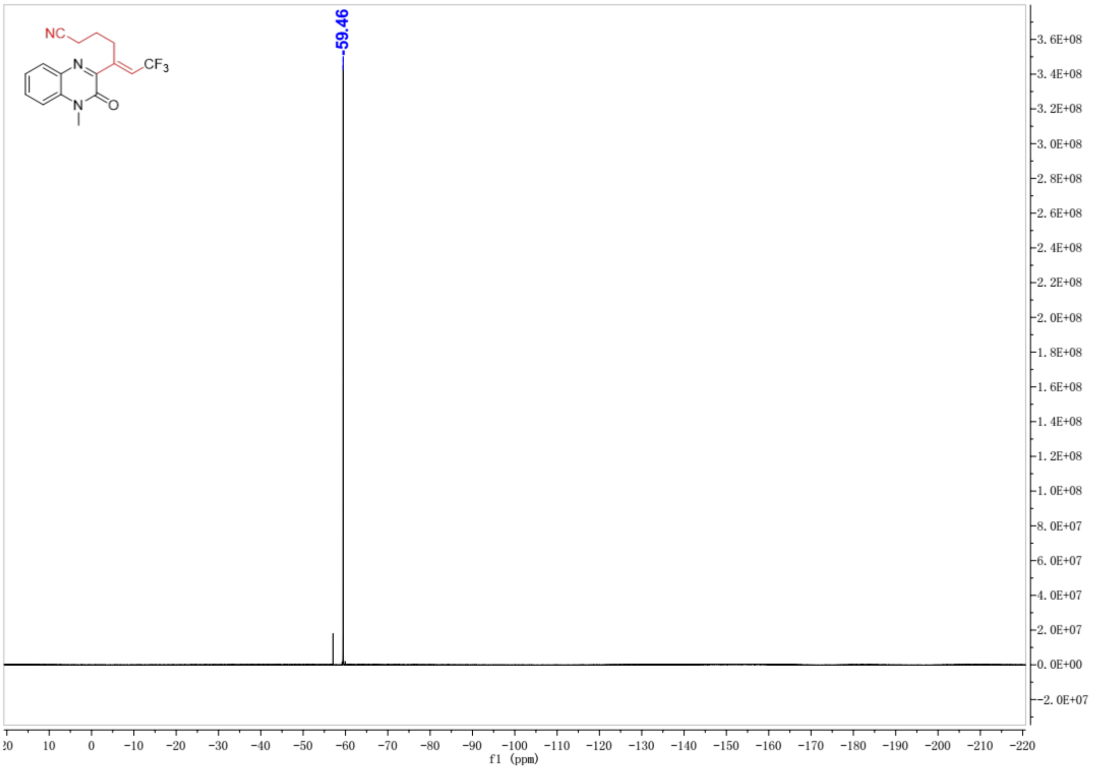

^{19}F NMR Spectra for **3ag**

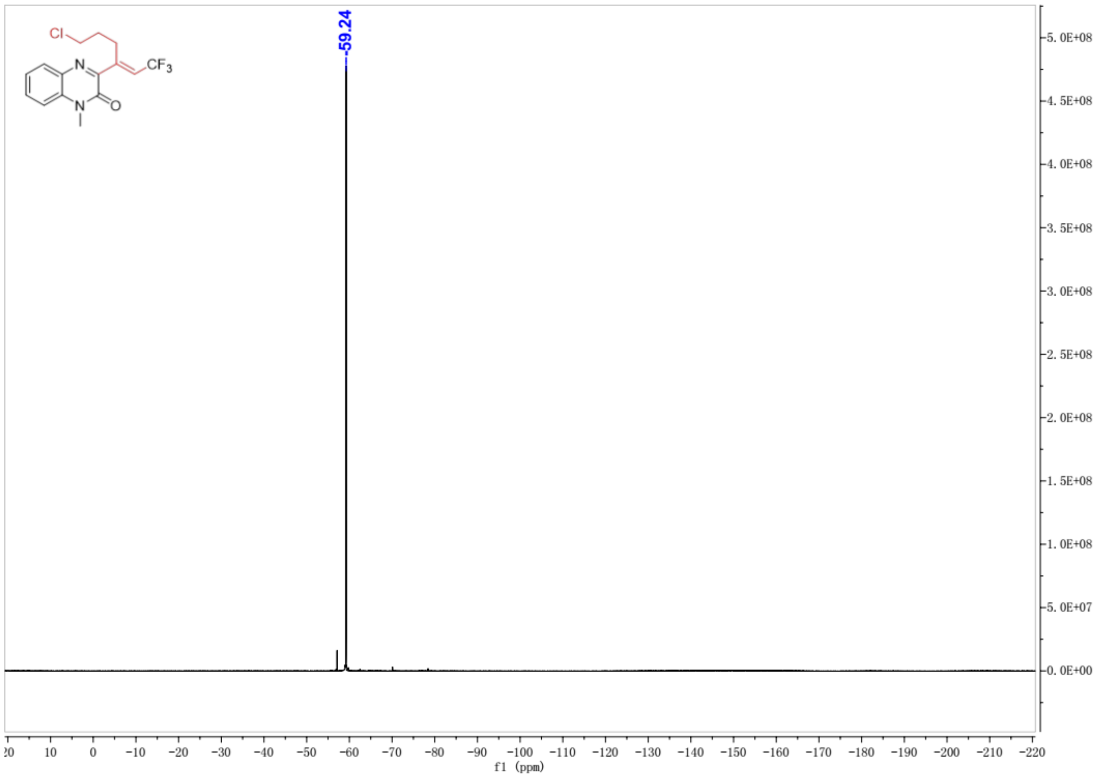

^{19}F NMR Spectra for **3ah**

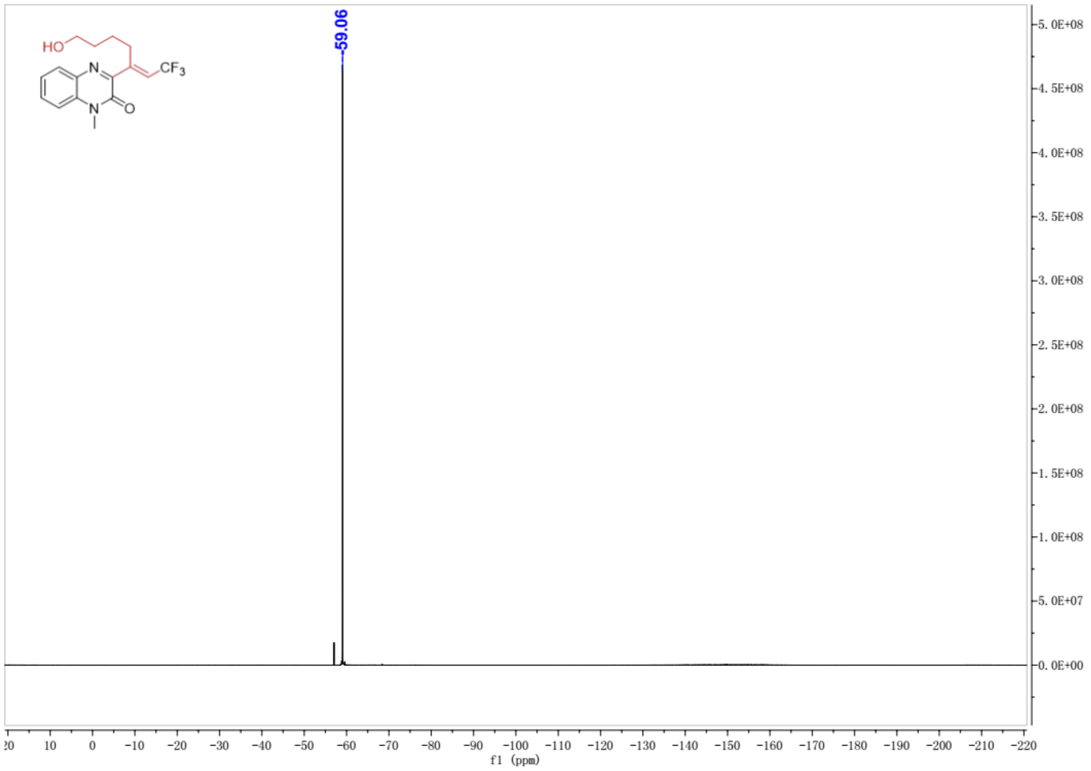

^{19}F NMR Spectra for **3ai**

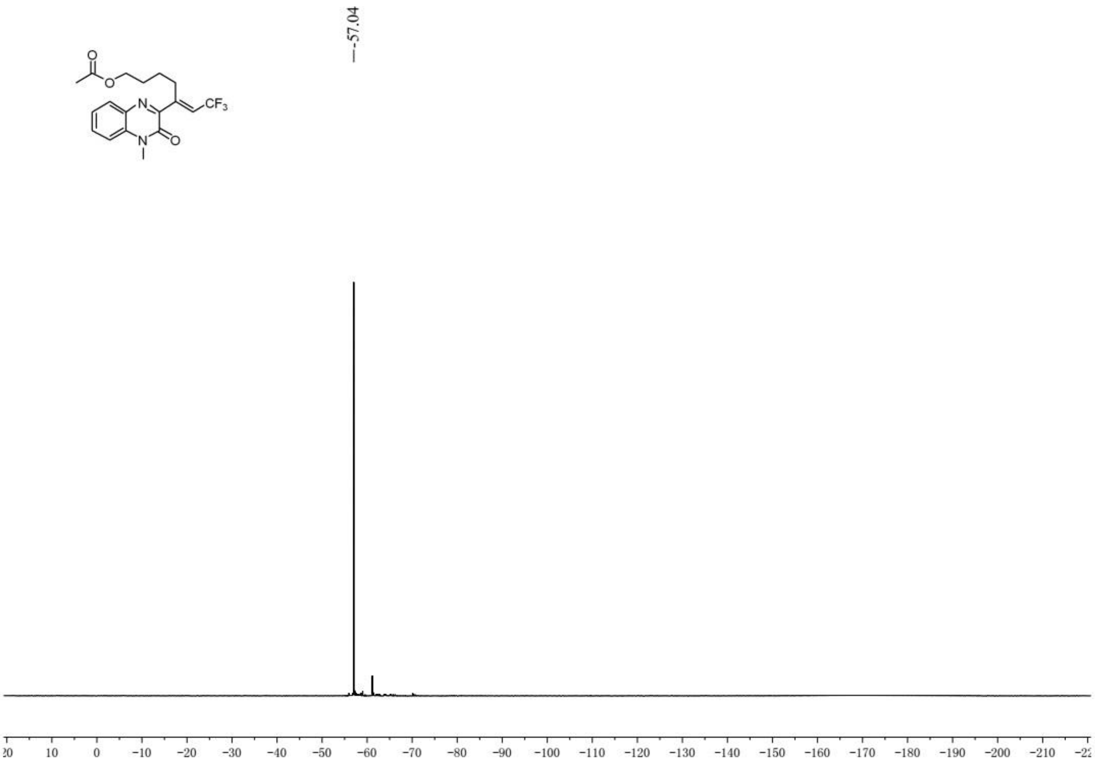

^{9}F NMR Spectra for **3aj**

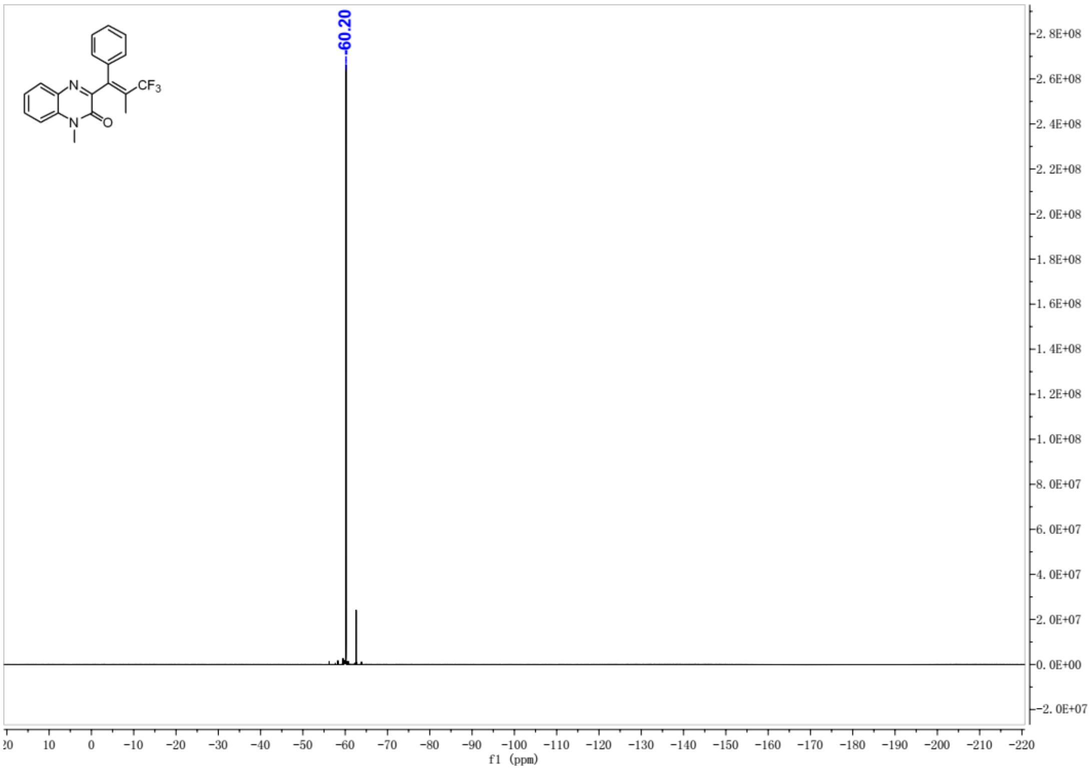

^{9}F NMR Spectra for **3ak**

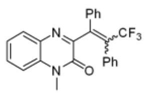

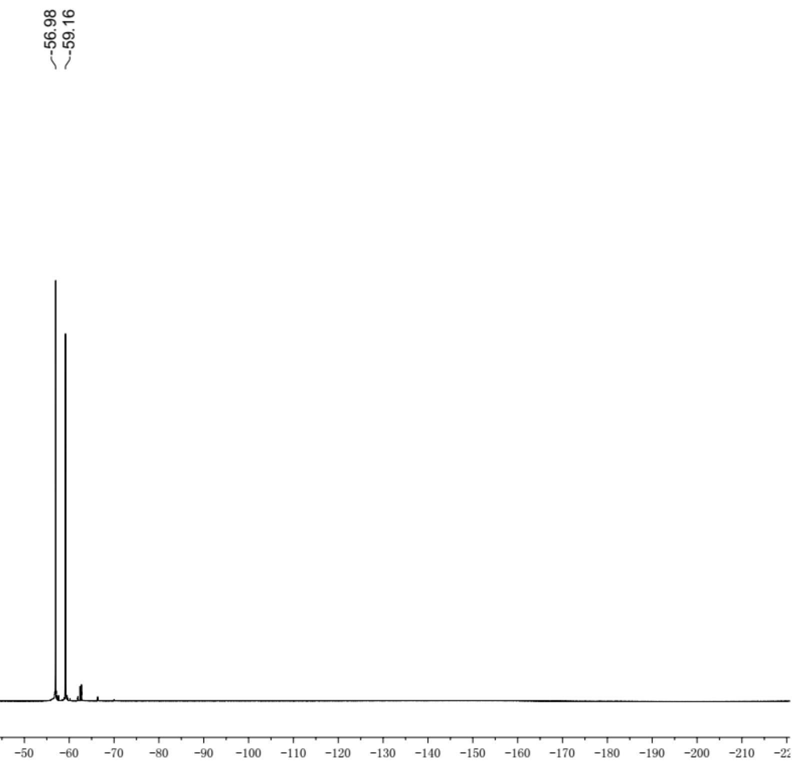

Supplement: RA-011-D1RA07014B-s001 [file RA-011-D1RA07014B-s001.pdf]
